# Tailored Phosphate Leaving Groups Direct Pathway-Dependent Self-Assembly

**DOI:** 10.1021/jacs.5c17237

**Published:** 2026-01-22

**Authors:** Arti Sharma, Kun Dai, Mahesh D. Pol, Anatoli Ioanna Katirtzidi Papadopoulou, Thejus Pramod, Ralf Thomann, Yi Thomann, Charalampos G. Pappas

**Affiliations:** † Freiburg Center for Interactive Materials and Bioinspired Technologies (FIT), 9174 University of Freiburg, Georges-Köhler-Allee 105, 79110 Freiburg, Germany; ‡ Institute of Organic Chemistry, University of Freiburg, Albertstrasse 21, 79104 Freiburg, Germany; § DFG Cluster of Excellence livMatS@FIT−Freiburg Center for Interactive Materials and Bioinspired Technologies, University of Freiburg, Georges-Köhler-Allee 105, 79110 Freiburg, Germany; ∥ Freiburg Materials Research Center (FMF), University of Freiburg, Stefan-Meier-Strasse 21, 79104 Freiburg, Germany

## Abstract

Phosphate esters and anhydrides are central to biology, storing and transferring chemical energy to sustain processes from metabolism to translation. Among them, acyl phosphates are highly reactive, yet biology channels their activation chemistry almost exclusively through aminoacyl adenylates. This conserved design leaves unexplored how alternative phosphate leaving groups might influence reactivity and structure. Here we show that aminoacyl phosphate esters with varied leaving groups (ethyl, phenyl, naphthyl, dodecyl) direct peptide bond formation and self-assembly through distinct pathways in water. Structural features of the leaving group guide preorganization into spherical aggregates before acyl transfer and influence coassembly with peptides after bond formation, imprinting outcomes that persist beyond activation. Consequently, the leaving group determines not only peptide yields, but also the supramolecular architectures and mechanical properties of assemblies arising from the same peptide sequences. In multicomponent mixtures, aminoacyl phosphates create recognition microenvironments in which aromaticity, hydrophobicity, or charge bias electrophile-nucleophile pairing, thereby transforming them from simple electrophilic reagents into active design elements capable of driving sequence selectivity. Moreover, soluble phosphates undergo phosphoryl exchange with orthophosphate, pyrophosphate, or adenosine monophosphate (AMP) to generate alternative intermediates that divert reactivity, whereas self-assembling phosphates resist exchange and favor amino acid oligomerization. These findings establish the leaving group as a tunable design element that governs reactivity, directs supramolecular organization and regulates pathway dynamics, transforming activation from a synthetic step into an active driver of recognition and assembly.

## Graphical abstract

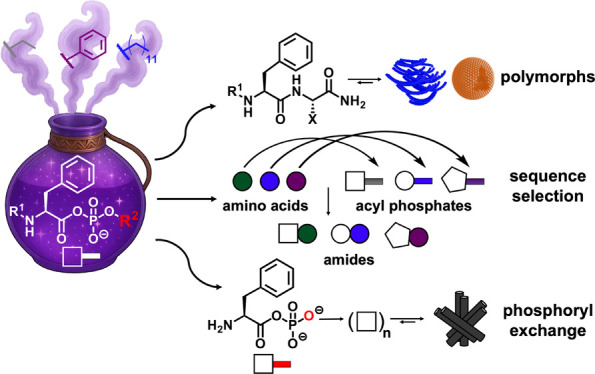

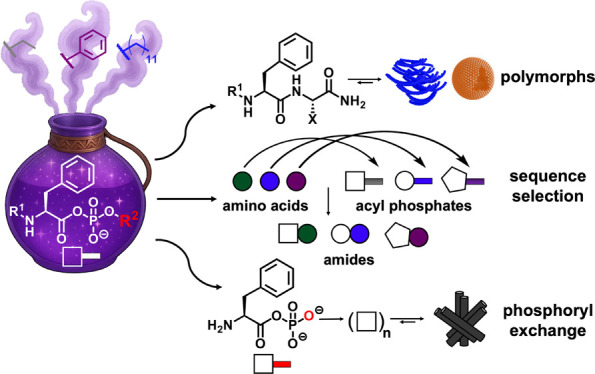

## Introduction

Phosphate esters and anhydrides^1,2^ are fundamental to life, exhibiting aqueous solubility and tunable reactivity, which in turn drives key biological processes with precision.^3^ Among them, acyl and phosphoryl transfer reactions play central roles in peptide bond formation,^4–6^ ubiquitin conjugation^7^ and lipid biosynthesis,^8,9^ processes that rely on activation of carboxylic acids. Acyl phosphates^10–12^ are particularly highly reactive species enabling rapid and selective transformations in aqueous environments.^13,14^ Notably, biology largely channels acyl phosphate chemistry through a single, conserved scaffold: the aminoacyl adenylate (aa-AMP).^15^ This molecule is central to translation,^16^ tRNA charging^17^ and biosynthetic activation,^18^ with its phosphate embedded in a nucleotide structure that ensures both kinetic control and enzymatic specificity. While acyl phosphate chemistry in biology is funnelled almost exclusively through aminoacyl adenylates, other classes of phosphate-containing molecules, which exhibit greater stability, reveal how small structural variations can direct different functions.^19^ From metabolic regulation by glucose phosphates to membrane organization^20^ by inositol phosphates, changes in phosphate linkage, or charge can dictate side chain recognition,^21^ selectivity^22^ and molecular interactions.^23^ These examples raise the question of what new functions might arise if acyl phosphates were endowed with similar structural diversity, allowing their leaving groups to act as recognition elements and impart new properties. Other activation strategies, such as thioesters,^24–26^ already demonstrate this potential. Thioesters are involved in a wide array of metabolic processes, from acetyl-CoA and succinyl-CoA in central metabolism to thioester-linked intermediates in fatty acid biosynthesis.^27^ These examples show how structural variation can be used to activate distinct biological functions.^28,29^ Such behavior highlights a broader principle, where activation chemistry is not only a way to form covalent bonds but can also embed recognition elements that bias subsequent interactions.^30^

In supramolecular chemistry,^31–34^ structure and activation pathways are tightly linked, where both molecular design and reaction environment impact the final architecture. Such dependence is often revealed by tuning external parameters, such as pH,^35^ ionic strength,^36^ solvent composition^37^ or by shifting between kinetic and thermodynamic regimes.^38,39^ Particularly, in peptide-based systems,^40–46^ differences in amino acid side chains^47,48^ or protecting groups^49^ are known to drive the formation of distinct structures by modulating noncovalent interactions.^50,51^ Yet in most studies, chemical activation is treated solely as a preparative step: peptides are synthesized under anhydrous conditions, and assembly is examined later in water, once reactive intermediates are no longer present. This distinction overlooks the scenario in which the structures of reactive intermediates formed in water could impact not only covalent bond formation but also supramolecular organization^52–56^ and material properties. Exceptions are found in nonequilibrium systems,^57–66^ where reaction sequence and the lifetime of transient species can critically determine which structures form and how long they persist. Yet, even in these dynamic systems, the activating agents are typically chosen for hydrolytic stability, while control is exerted primarily through tuning reaction kinetics.^67–70^ Far less attention has been directed toward the chemical structure of the fuels,^30^ and of the waste products they generate, even though these features strongly influence both activation and deactivation pathways.^71–75^ Therefore, if the activation site, such as the *C*-terminus of peptides, is equipped with leaving groups that also serve as structural or recognition elements, reactive intermediates could influence assembly beyond merely facilitating bond formation. These species may transiently engage in noncovalent interactions either before or after covalent bond formation, thereby guiding selectivity and self-assembly through different pathways. This is particularly relevant in oligomerization reactions involving N-terminus-free aminoacyl phosphates,^12,13,76,77^ where assembly and reactivity of the activated species determine chain growth and product distribution. In these systems, additional reactions, such as phosphoryl transfer^5^ can generate alternative activated intermediates and redirect acyl-transfer pathways. Similar behavior is also observed in other abiotic peptide-forming strategies,^78–80^ such as *N*-carboxyanhydrides (NCAs),^4,6,81^ thioesters,^82^ and wet–dry cycling approaches,^83^ which rely on activated monomers whose stability, solubility and microenvironment constrain the accessible oligomerization pathways. Despite different approaches, the use of leaving groups as structural elements that direct both reactivity and self-assembly remains largely unexplored and offers a promising design principle for impacting reaction pathways.

Herein, we investigate this potential by using aminoacyl phosphate esters as structural intermediates that couple abiotic peptide bond formation with supramolecular assembly. Through variation of the phosphate leaving group, from short aliphatic (ethyl), aromatic (phenyl and naphthyl) and long-chain aliphatic (dodecyl) structures, we uncover how these modifications drive distinct supramolecular behaviors. Each acyl phosphate reacts with amino acid nucleophiles to yield the same peptide product, yet the resulting assemblies differ markedly, ranging from soluble species to droplets, fibers and hydrogels. These differences arise not only from the ability of acyl phosphates to preorganize into distinct supramolecular arrangements prior to acyl transfer, but also from the influence of the cleaved leaving groups, which can coassemble with the reaction products (Scheme 1). As a result, the amphiphilic nature of the leaving group can exert a lasting impact on the assembly pathway and ultimately affect the supramolecular structures of the resulting peptides. In mixtures, aminoacyl phosphates pair selectively with amino acids, yielding sequence-specific amides, while suppressing alternative coupling products. This selectivity arises from the ability of certain phosphate esters to engage in noncovalent interactions prior to amide bond formation. Whereas soluble esters react randomly in solution, those prone to aggregation generate local microenvironments that facilitate selective coupling through structural complementarity. Moreover, in oligomerization reactions involving *N*-terminus free aminoacyl phosphates, phosphoryl exchange can redirect acyl transfer pathways. Readily exchangeable groups, such as ethyl or phenyl phosphate, undergo in situ exchange with orthophosphate, pyrophosphate, or AMP, generating alternative intermediates such as acyl diphosphates and acyl adenylates. While such species readily form, their high charge density make them hydrolytically labile. In contrast, dodecyl phosphate resists such exchange due to its propensity to self-assemble, shielding the activated species from hydrolysis and promoting oligomerization. Taken together, these findings establish the supramolecular properties of the leaving group as a design variable in acyl phosphate chemistry, allowing control over peptide bond formation, reaction pathways and supramolecular organization. In contrast to biology’s reliance on adenylates, diverse abiotic phosphate esters and their leaving groups can steer acyl transfer and phosphoryl exchange along distinct pathways.

**Scheme 1:**
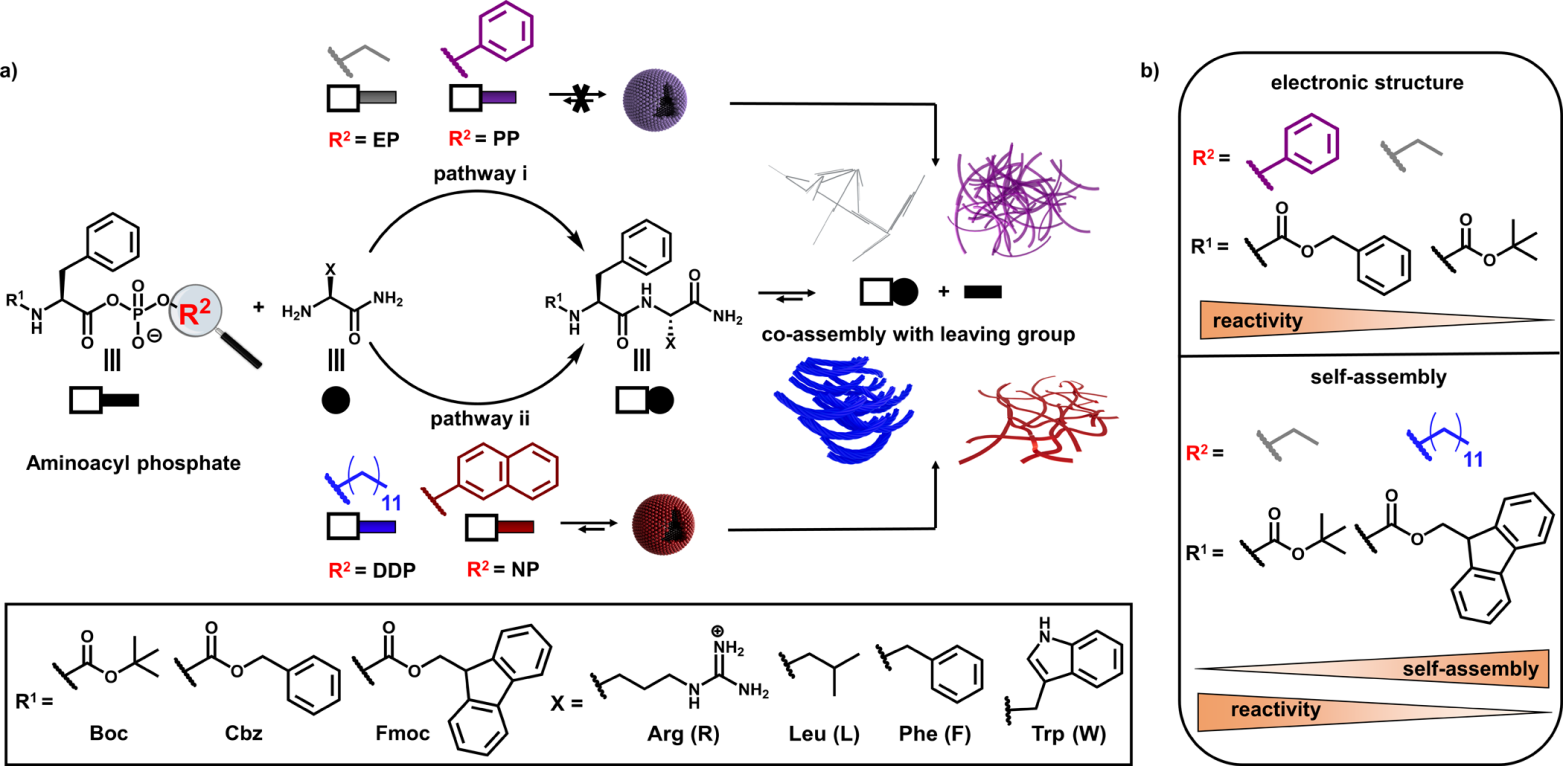
(a) Peptide Bond Formation from Aminoacyl Phosphate Esters, Highlighting How the Phosphate Leaving Group (R^2^) Shapes Both Acyl-Transfer Reactivity and the Supramolecular Structures Formed after Reaction. Although the Covalent Product is the Same, Different Phosphate Tails Direct Distinct Assembly Pathways through Preorganization (Prior to Acyl Transfer) and Co-Assembly with the Product and (b) Parameters Affecting Reactivity: (Top) Electronic Effects and (Bottom) the Self-Assembly Propensity of the Phosphate Leaving Group (*R* ^2^) and N-Terminal Protecting Group (R^1^)

## Results and Discussion

### Designing Phosphate Leaving Groups

We previously utilized aminoacyl phosphate esters to drive acyl transfer reactions leading to either stable amide bonds^12^ or transient (thio)-esters.^84^ In these systems, the primary structural variation resided in the amino acid side chain, which influenced both reactivity and self-assembly, thereby dictating the outcome of the acyl transfer reaction. More specifically, we demonstrated that amino acid side chains can direct peptide oligomerization, enabling assembly of homo-oligomers,^12^ covalent self-sorting^14^ and control over microenvironment through phase separation.^85^ Notably, transfer of aromatic side chains onto ester or thioester intermediates promoted self-assembly, which stabilized otherwise short-lived species and enabled sequential acylation in dynamic environments.^86^ Herein, we shift the focus from amino acid side chain modifications to the phosphate leaving group. We synthesized a series of *N*-terminally protected phenylalanine-derived aminoacyl phosphate esters bearing different leaving groups, including aliphatic (ethyl, dodecyl) and aromatic (phenyl, naphthyl) moieties. We reasoned that altering the “phosphate tail” could simultaneously influence the reactivity and the propensity for self-assembly, thus affecting the pathway of covalent bond formation and the strength of noncovalent interactions at different stages of the acyl transfer. We used *N*-terminally protected phenylalanine with either Boc (*tert*-butoxycarbonyl) or Z (benzyloxycarbonyl) group. Then, we modified the *C*-terminus by introducing phosphate groups with various substituents: ethyl (**FEP**), phenyl (**FPP**), naphthyl (**FNP**) and dodecyl (**FDDP)**, (Figure 1a). Confocal and cryo-transmission electron microscopy (cryo-TEM) showed that Boc- and Z-protected **FEP** and **FPP** were not capable of aggregation, whereas the **FNP** and **FDDP** derivatives formed aggregates (Figures 1b,c and S1). Although confocal microscopy did not reveal assemblies for **Boc-FNP**, cryo-TEM analysis confirmed the presence of aggregates. This observation is further supported by dynamic light scattering (DLS) measurements, which also indicated that **Boc-FNP** undergoes aggregation under the same conditions (Figure S2). To compare their reactivity, we determined the half-lives of the aminoacyl phosphates using Ultra-Performance Liquid Chromatography (UPLC). **Boc-FEP**, **Boc-FPP**, **Boc-FNP** and **Boc-FDDP** displayed hydrolysis half-lives of 130, 78, 86, and 474 min, respectively. The corresponding Z derivatives, (**Z-FEP**, **Z-FPP**, **Z-FNP**, and **Z-FDDP**) showed half-lives of 87 min, 46 min, 186 and 744 min (Figure S3). In both series, the long-chain dodecyl phosphate esters (**FDDP**) displayed the highest stability, likely due to aggregation that shielded reactive moieties from hydrolysis. To further assess whether the reduced reactivity of **Boc-FDDP** originates from its aggregation behavior, we measured its hydrolysis half-life in a 60% DMSO/water mixture, a condition where self-assembly should be disrupted. Under these non-assembling conditions, the half-life decreased to 173 min compared to 474 min in aqueous buffer (Figure S4). This significant reduction indicates that aggregation plays an important role in stabilizing **Boc-FDDP** against hydrolysis.

**Figure 1:**
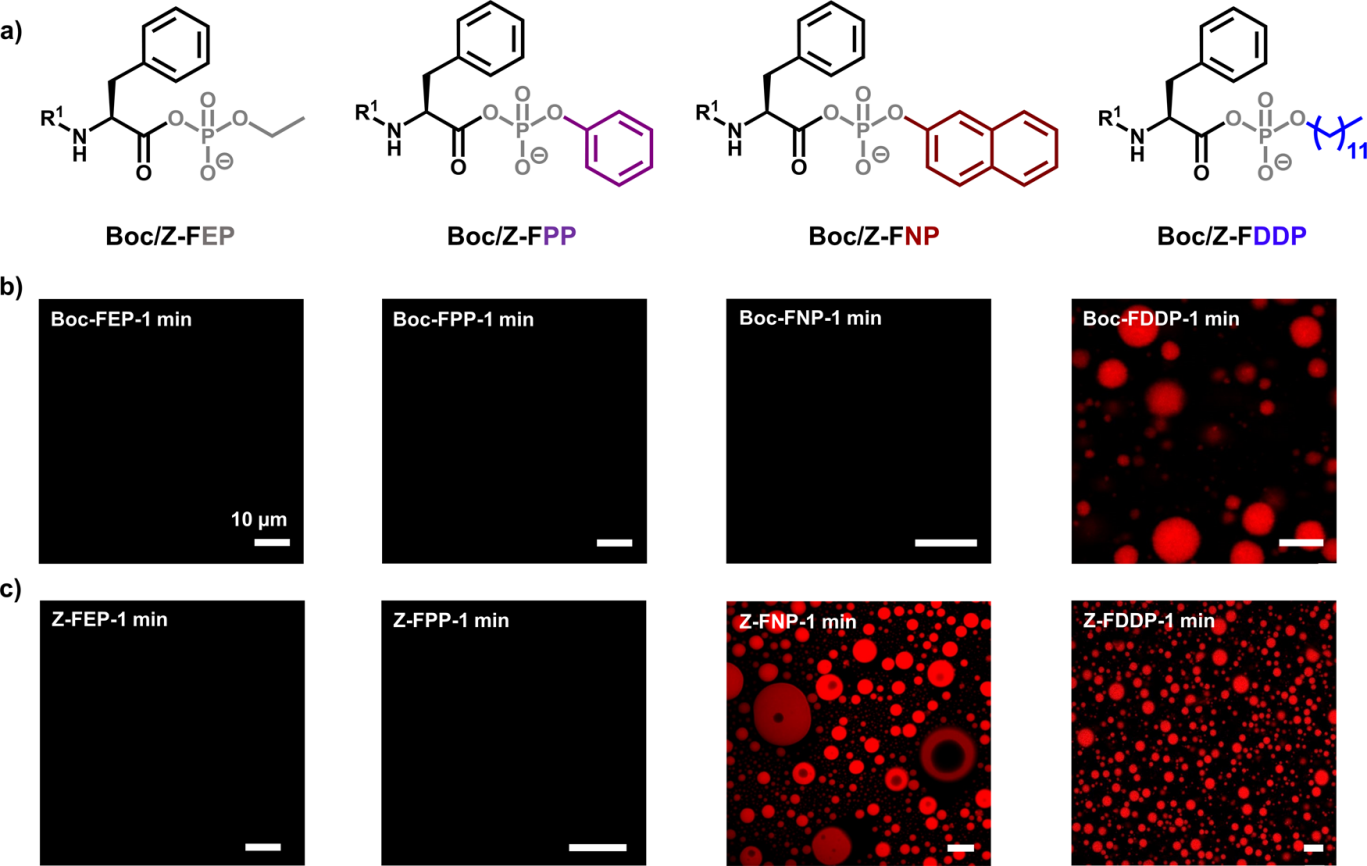
(a) Chemical structures of Boc/Z-protected series of aminoacyl phosphate esters. (b, c) Confocal microscopy images (nile red staining) of 10 mM **Boc/Z-FEP**, **Boc/Z-FPP**, **Boc/Z-FNP**, **Boc/Z-FDDP** (left to right). Images were acquired immediately after dissolving the acyl phosphates in 0.6 M borate buffer, pH 9.1. Scale bar for all images is 10 μm.

To further probe the nature of the assemblies formed by the phosphate esters, we conducted dye-partitioning experiments using Alexa Fluor 488 (AF488) and Nile Red. These dyes preferentially localize in hydrophilic and hydrophobic environments, respectively. These experiments allowed us to assess the internal polarity of the spherical aggregates. Assemblies formed by **Z-FNP** strongly accumulated AF488 at the core, indicating a hydrophilic interior surrounded by more hydrophobic domains. In contrast, compartments formed by **Boc-FDDP** and **Z-FDDP** showed intense Nile Red staining, suggesting a predominantly hydrophobic environment throughout the entire structure (Figure S5). To compare the influence of *C*-terminal phosphate tails with a strong N-terminal aggregation motif, we synthesized Fmoc-protected phenylalanine esters with ethyl and phenyl phosphate moieties. Due to the inherent aggregation propensity of the Fmoc group,^49^ both **Fmoc-FEP** and **Fmoc-FPP** formed supramolecular assemblies regardless of phosphate moiety (Figure S6). **Fmoc-FEP** and **Fmoc-FPP** exhibited hydrolysis half-lives of 1123 and 378 min, respectively, in line with the reactivity trends observed for the Boc- and Z-protected derivatives (Figure S7). These findings highlight that aromatic phosphate esters hydrolyze more rapidly than their aliphatic counterparts, potentially through inductive effects that influence the electrophilicity of the carbonyl around the phosphate moiety.^87^ The differences in reactivity and assembly demonstrate that the design of the phosphate ester can modulate both reaction kinetics and supramolecular behavior prior to acyl transfer.

### Pathway-Dependent Assembly Directed by the Leaving Group

Having demonstrated that the phosphate moiety strongly influences both the structure and reactivity of aminoacyl phosphate esters, we next investigated how these variations affect amide bond formation when reacting with amino acid nucleophiles. Specifically, we investigated how the self-assembly of acyl phosphates impacts not only reaction yields but also the supramolecular properties of the resulting dipeptides. In addition, we asked whether the cleaved phosphate ester, though acting as a leaving group, might continue to shape structural organization through coassembly during bond formation. Four amino acid amides were chosen as nucleophiles, including arginine (R-NH_2_), leucine (L-NH_2_), phenylalanine (F-NH_2_), and tryptophan (W-NH_2_). Amides were selected over carboxylates to minimize electrostatic repulsion with the negatively charged phosphate esters. Initially, four Boc-protected aminoacyl phosphate esters, **Boc-FEP**, **Boc-FPP**, **Boc-FNP** and **Boc-FDDP** reacted with arginine (R-NH_2_) to generate the corresponding Boc-FR-NH_2_ dipeptides. Each reaction was carried out using 10 mM of the phosphate ester and 10 mM of R-NH_2_ (1:1 ratio) in 0.6 M borate buffer at pH 9.1 (Figure 2a). UPLC analysis performed after 1 h of initiating the reactions revealed yields of 68%, 83%, 59%, and 90% for the ethyl (**EP**), phenyl (**PP**), naphthyl (**NP**), and dodecyl (**DDP**) derivatives, respectively (Figures 2b and S8–S9). The higher yields obtained from **Boc-FPP** and **Boc-FDDP** suggested that enhanced reactivity and potentially self-assembly increase acyl transfer efficiency. Notably, **Boc-FDDP** showed the highest conversion, minimizing hydrolysis through self-assembly. Interestingly, the structural behavior of the resulting Boc-FR-NH_2_ dipeptides varied depending on the phosphate ester used. Products from **Boc-FEP** and **Boc-FPP** remained soluble throughout the reaction, whereas those derived from **Boc-FNP** and **Boc-FDDP** underwent self-assembly (Figure 2a). These observations were supported through time-dependent turbidity experiments (Figure 2c). The dipeptide from **Boc-FDDP** formed a milky suspension, while the **Boc-FNP**-derived product precipitated as dense, insoluble aggregates. Time-dependent confocal microscopy revealed that Boc-FR-NH_2_ originated from **Boc-FDDP** evolved into spherical aggregates, (Figure 2d and Supporting video S1). TEM imaging confirmed the presence of these structures after reaction completion (Figure S10). In the case of the **Boc-FNP-**derived dipeptide, smaller droplets initially formed and gradually transitioned into larger, insoluble aggregates (Figure 2a,e). This behavior may be driven by ion-π interactions between the guanidinium group of arginine and the naphthyl moiety of the phosphate ester. Scanning Electron Microscopy (SEM) revealed that these aggregates exhibited a porous morphology, with observed pore sizes ranging from 0.9 to 2.5 μm. (Figure 2f). The porosity might arise from incomplete phase separation or hindered coalescence, driven by a combination of hydrophobic and electrostatic interactions. To assess the composition of the aggregated phase, we performed centrifugation experiments to isolate the solid material, followed by dissolution in acetonitrile and UPLC analysis. The isolated structure was found to contain Boc-F, Boc-FR-NH_2_, and cleaved naphthyl phosphate (**NP**), confirming that the leaving group remains associated with the product and contributes to self-assembly (Figures S11–S12).

**Figure 2:**
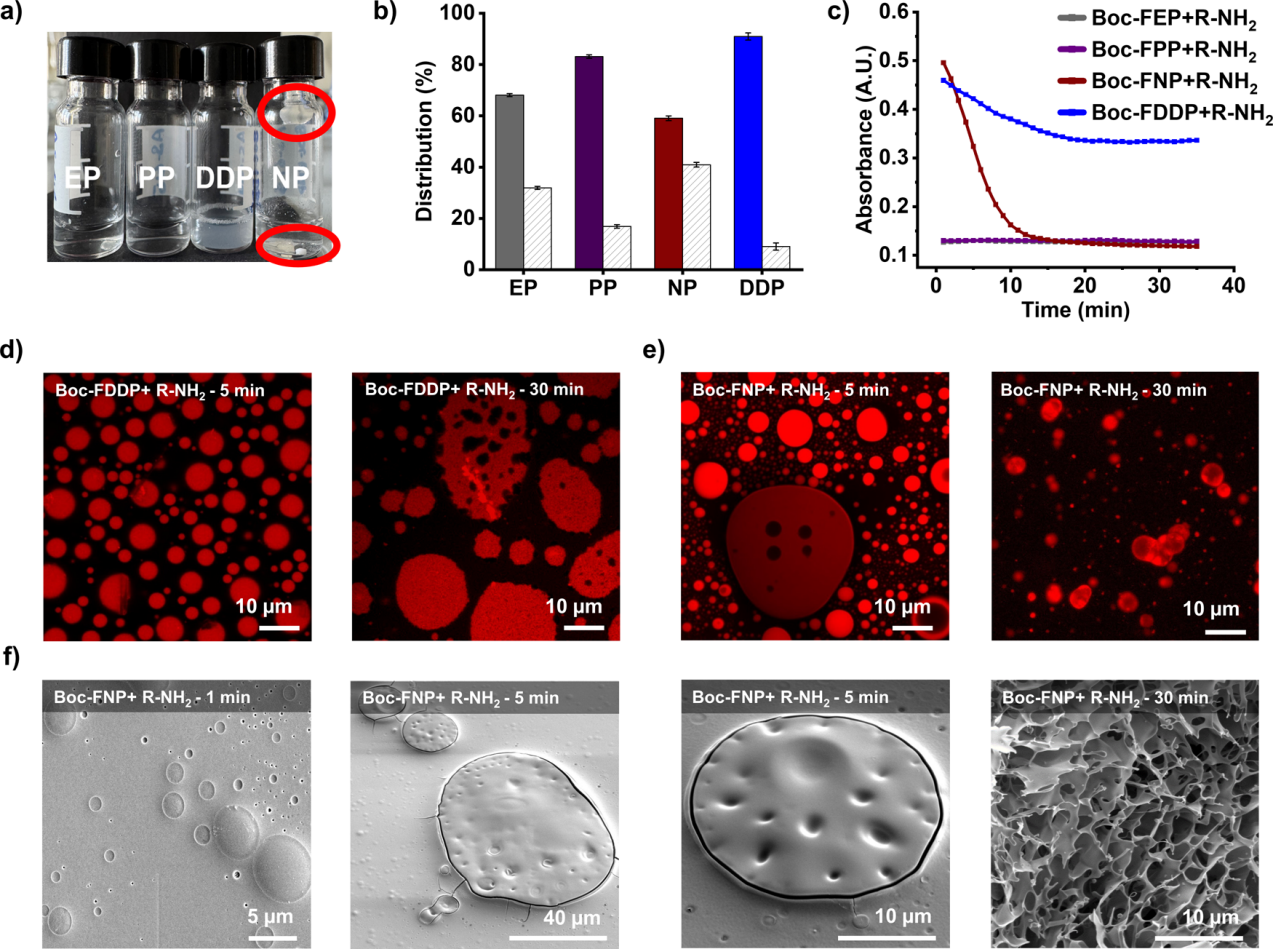
(a) Digital images of reaction vials from peptide coupling between 10 mM R-NH_2_ and Boc-protected aminoacyl phosphates (**EP**, **PP**, **NP**, and **DDP**). (b) Corresponding peptide products under the same conditions. Solid bars represent peptide coupling (Boc-FR-NH_2_), while striped bars indicate hydrolysis (**Boc-F-OH**). Distribution of peptide species was determined 1 h after initiating the reaction. Error bars represent the standard deviation of three independent experiments. (c) Time-dependent absorbance (turbidity) measurements for the same reactions. (d, e) Time-dependent confocal images (nile red staining) of reactions between 10 mM R-NH_2_ and 10 mM **Boc-FDDP** or 10 mM **Boc-FNP**, respectively. (f) Time-dependent SEM images of reaction between 10 mM R-NH_2_ and 10 mM **Boc-FNP**.

We next explored an aliphatic amide, (L-NH_2_), as a nucleophile. Following the same protocol, **Boc-FEP**, **Boc-FPP**, **Boc-FNP** and **Boc-FDDP** were reacted with L-NH_2_ to generate the corresponding Boc-FL-NH_2_ dipeptides, with yields of 69%, 83%, 76% and 73% respectively (Figures 3a,b and S13–S14). Unlike the R-NH_2_ series, which showed pronounced yield differences, coupling with L-NH_2_ gave consistently high conversions across all phosphate esters. Nevertheless, we observed distinct supramolecular structures of Boc-FL-NH_2_ depending on the phosphate ester used. The reaction with **Boc-FEP** led to amorphous aggregates and the reaction with **Boc-FPP** yielded a fibrillar network. In contrast, Boc-FL-NH_2_ from **Boc-FDDP** gave rise twisted fibers (Figure 3d), while the product from **Boc-FNP** assembled into ribbons (Figure 3f). Time-dependent confocal microscopy revealed a transition from spheres to fibers for the **Boc-FDDP** system, consistent with the samples produced from R-NH_2_ (Figure 3d, 3e and Supporting video S2).

**Figure 3:**
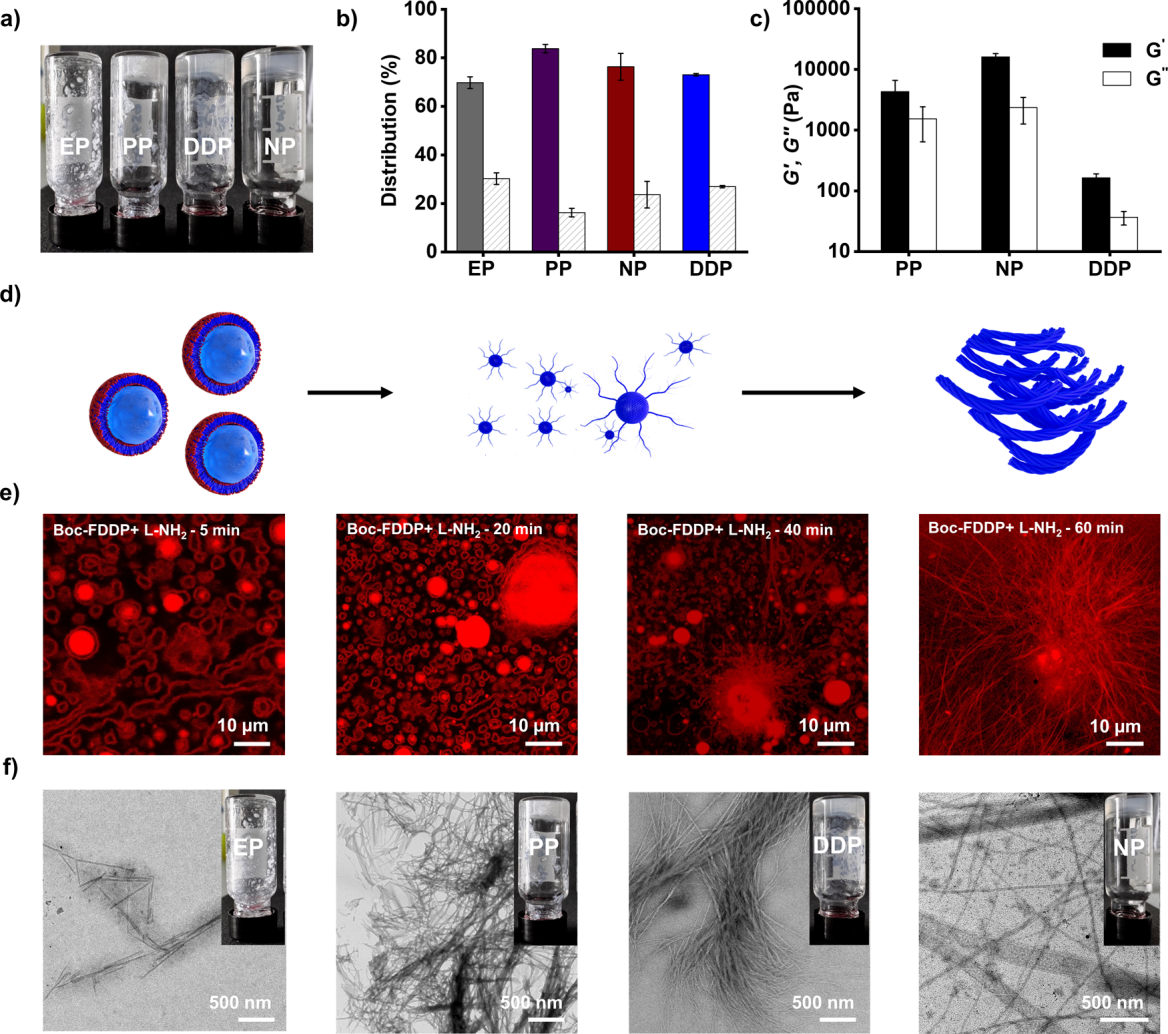
(a) Digital images of reaction vials from peptide coupling between 10 mM L-NH_2_ and Boc-protected aminoacyl phosphates (**EP**, **PP**, **NP**, and **DDP**). (b) Corresponding peptide products under the same conditions. Solid bars represent the peptide coupling (Boc-FL-NH_2_) while striped bars indicate hydrolysis (**Boc-F-OH**). Distribution of peptide species was determined 1 h after initiating the reaction. Error bars represent the standard deviation of three independent experiments. (c) Storage (*G*′) and loss modulus (*G*″) of samples formed from the reaction between 10 mM L-NH_2_ and Boc-protected aminoacyl phosphates (**PP**, **NP** and **DDP**) in equimolar concentrations. Error bars represent standard deviation from three independent experiments. (d) Schematic illustration of the self-assembly process, demonstrating the reconfiguration of spherical assemblies to fibrillar assemblies. (e) Time-dependent confocal images (nile red staining) of reaction between 10 mM L-NH_2_ and 10 mM **Boc-FDDP**. (f) TEM images showing assemblies obtained from reactions of Boc-protected aminoacyl phosphates with L-NH_2_, with inset images displaying the macroscopic behavior of the sample.

Oscillatory rheology revealed that all three gel-forming systems, (dipeptides from **Boc-FPP**, **Boc-FNP**, and **Boc-FDDP)** exhibited viscoelastic behavior with storage moduli (*G*′) exceeding loss moduli (*G*″) (Figure 3c). The gels originating from the **Boc-FNP** and **Boc-FPP** reactions displayed higher *G*′ values, correlating with the more entangled fiber networks observed via confocal (Figure 3e) and TEM (Figure 3f) microscopy. The enhanced mechanical properties likely arise from aromatic stacking interactions between the naphthyl or phenyl phosphate esters with the dipeptides, promoting robust fibrillar self-assembly. In order to directly test this, we synthesized Boc-FL-NH_2_ and mix it with the corresponding phosphate salts (**EP**, **PP**, **NP** and **DDP**) to evaluate whether the cleaved phosphates coassemble with the dipeptide. The dipeptide showed limited solubility in buffer. Mixtures with **EP** salt led to precipitation, whereas **PP**, **NP**, and **DDP** salts gave rise to hydrogel formation. The gel morphology varied with the salt type, showing a ribbon-like fibrillar network with **NP**, thin fibers with **PP** and a dense twisted fibrillar architecture with **DDP** salt (Figure S15). Centrifugation experiments further confirmed that the final fibrillar architectures arise from coassembly of the dipeptide Boc-FL-NH_2_ with the respective phosphate salts. For instance, in the samples where **Boc-FPP** and **Boc-FNP** were reacted with L-NH_2_, the aggregated phase was separated by centrifugation and subsequently dissolved in THF/H_2_O (1:1). UPLC analysis confirmed the coexistence of **PP** and **NP** salts with the dipeptide aggregates (Figures S16–S17). These results demonstrate that the cleaved phosphate esters contribute to the formation and stabilization of the supramolecular structures during acyl transfer.

Furthermore, we tested aromatic amino acids as nucleophiles. Reactions with phenylalanine (F-NH_2_) yielded Boc-FF-NH_2_ in moderate to high conversions (64–87%) across all phosphate esters, but unlike previous systems, the resulting aggregates were morphologically similar (Figures S18–S21). Similarly, reactions with tryptophan (W-NH_2_) proceeded in high yields (88–93%), with gel formation observed only for the **Boc-FDDP**-derived product (Figures S22–S25). To evaluate whether the assembly behavior observed in the Boc series also applies to other protecting groups, we next investigated Z-protected aminoacyl phosphate esters. As mentioned earlier, **EP** and **PP** derivatives remained soluble, while the naphthyl and dodecyl variants self-assembled into spherical structures. Consistent with the Boc-series, we observed clear differences in self-assembly depending on the phosphate ester and nucleophile used (R-NH_2_, L-NH_2_, F-NH_2_ and W-NH_2_), further supporting the link between phosphate esters and supramolecular structures (Figures S26–S42). In contrast, Fmoc protection led both **FEP** and **FPP** derivatives to form spherical aggregates, driven by the strong aggregation propensity of the Fmoc group. Reactions with amino acid amides produced assemblies that were morphologically similar (Figures S43–S53).

The distinct assemblies observed from different phosphate esters suggested that in addition to chemical inputs, physical forces might also affect assembly pathways. To probe this, we examined the influence of mechanical forces on peptide self-assembly, which have been previously demonstrated to trigger gelation^88^ and drive nonequilibrium functions.^89^ Specifically, 10 mM **Boc-FDDP** was reacted with L-NH_2_ under equimolar conditions in 0.6 M borate buffer (pH 9.1), while varying the mechanical conditions: magnetic stirring at 300 or 800 rpm, or sonication for 1 h. Product yields determined by UPLC, were comparable across all conditions (66–68%) and to those obtained in the absence of mechanical force. However, the resulting assemblies diverged, where stirring gave rise to less-defined assemblies and sonication preserved gelation, as confirmed by TEM (Figures S54–S56).

### Sequence Selection from Acyl Phosphate-Nucleophile Matching

Having established that the structure of the phosphate ester influences both self-assembly and the properties of peptide products, we next investigated whether these effects could extend to selective amide bond formation in mixtures. We reasoned that phosphate esters could also act as recognition elements guiding preferential electrophile-nucleophile pairing through noncovalent interactions. Thus, we designed a series of experiments using a mixture of aminoacyl phosphate esters bearing diverse R^2^ side chains and N-terminal protecting groups (Figure 4a). These esters were classified into two categories: assembling phosphates, which feature structural motifs that promote supramolecular organization, and nonassembling, soluble controls (Figure 4b). Each phosphate ester mixture was reacted with an equimolar mixture of three amino acid amides: aspartic acid (D-NH_2_), arginine (R-NH_2_), and tryptophan (W-NH_2_). In the first set of experiments (Figure 4c), a mixture of **Boc-FEP**, **Z-FDDP** and **Fmoc-FPP** was exposed to the nucleophile pool. The resulting products showed clear preferences: **Boc-FEP** predominantly formed the Boc-FD-NH_2_ dipeptide, **Z-FDDP** reacted preferentially with R-NH_2_ to give Z-FR-NH_2_ and **Fmoc-FPP** selectively coupled with W-NH_2_ to yield Fmoc-FW-NH_2_. In case of **Fmoc-FPP**, π–π stacking likely underlies the preference for W-NH_2_, while the long dodecyl chain of **Z-FDDP** may promote hydrophobic clustering with R-NH_2_ (Figures S57–S58). This interaction is potentially stabilized by electrostatic complementarity between the positively charged guanidinium group of arginine and the negatively charged phosphate. Notably, **Boc-FEP**, being highly soluble and lacking aggregation, favored coupling with D-NH_2_. The absence of FD-NH_2_ products in the reactions involving **Z-FDDP** and **Fmoc-FPP** points that electrostatic repulsion between the negatively charged amino acid and the phosphate ester excludes aspartic acid from assembling compartments. As a result, coupling proceeds with nucleophiles that match with phosphate esters featuring long aliphatic or aromatic residues. These findings illustrate how self-assembly of aminoacyl phosphates govern electrophile-nucleophile pairing in multicomponent mixtures. To further confirm that the selection mechanism depends on assembly, we repeated the mixture experiment in the presence of a co- solvent to disrupt aggregation. Under these conditions, the product distribution became consistent with nonselective (statistical) coupling, with the exception that **Fmoc-FPP** still did not couple with D-NH_2_ (Figures S59–S60). This observation prompted us to investigate electrophile-nucleophile pairing more specifically by performing individual reactions of each assembling and non-assembling phosphate ester from the mixture with D-NH_2_ alone. We found that the assembling phosphates, **Boc-FDDP**, **Z-FDDP**, **Fmoc-FEP** and **Fmoc-FPP** showed significantly lower coupling yields under equimolar conditions (Figures S61–S66). In contrast, the non-assembling phosphates **Boc-FEP** and **Z-FEP** exhibited substantially higher yields. Interestingly, **Z-FNP** and **Boc-FNP**, although capable of aggregation, afforded coupling efficiencies comparable to the non-assembling **Z-FEP**. To further probe the role of the phosphate ester, we swapped the ester backbones (Figure 4d) and tested a new mixture: **Boc-FDDP**, **Z-FEP** and **Fmoc-FPP**. Notably, selectivity persisted, as **Fmoc-FPP** still coupled with W-NH_2_, **Boc-FDDP** with R-NH_2_ and **Z-FEP** with D-NH_2_, highlighting that recognition is stored within the phosphate ester, and is not dominated by the N-terminal protecting group (Figures S67 and S68). Another mixture contained **Boc-FEP**, **Z-FNP**, and **Fmoc-FEP** in order to test whether replacing the phenyl phosphate of **Fmoc-FPP** with an ethyl group (**Fmoc-FEP**) could shift selectivity by removing the π- system from the phosphate ester which was required for interaction with W-NH_2_ (Figure 4e).

**Figure 4:**
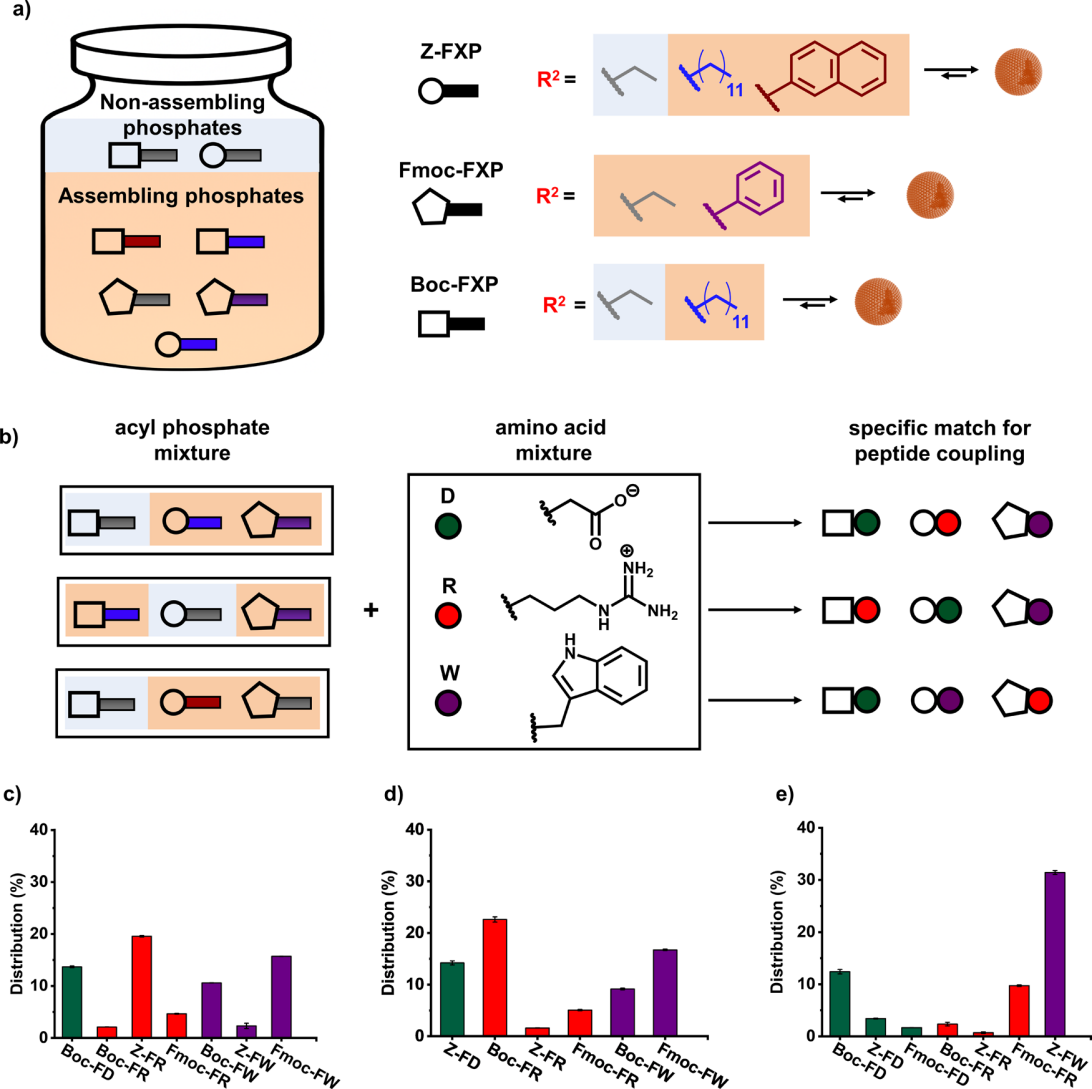
Selective peptide coupling in multicomponent mixtures directed by assembling versus non-assembling aminoacyl phosphates. (a) Schematic representation of assembling and non-assembling aminoacyl phosphate derivatives. (b) Experimental design for the three mixture studies, showing how noncovalent interactions guide selective peptide bond formation between the nucleophiles (D, R, W) and the different acyl phosphates. (c–e) Product distributions from reactions of nucleophile mixtures (D, R, W; 10 mM each) with phosphate ester mixtures composed of (c) **Boc-FEP**, **Z-FDDP**, **Fmoc-FPP**; (d) **Boc-FDDP**, **Z-FEP**, **Fmoc-FPP**; (e) **Boc-FEP**, **Z-FNP**, **Fmoc-FEP**. All reactions were carried out in 0.6 M borate buffer at pH 9.1. Product distribution was determined 1 h after initiating the reaction. Error bars represent the standard deviation of three independent experiments.

Notably in this system, W-NH_2_ coupled with **Z-FNP**, where the naphthyl group enables π–π stacking, while **Fmoc-FEP** favored reaction with R-NH_2_ (Figures S69 and S70). Having observed that W-NH_2_, R-NH_2_, and D-NH_2_ display distinct preferences in the three-component mixture experiments, we next examined whether the selectivity associated specifically with W-NH_2_ is preserved when it is the only nucleophile present. This step allowed us to disentangle intrinsic electrophile-nucleophile matching from competitive effects arising in the full ternary mixtures.

Consistent with the behavior observed in the three-nucleophile system, W-NH_2_ continued to preferentially react with aromatic phosphate esters. For example, when **Boc-FEP**, **Z-FDDP** and **Fmoc-FPP** were combined with 10 mM W, the nucleophile showed a strong preference for **Fmoc-FPP** over **Boc-FEP** and **Z-FDDP** (Figures S71–S72). A second mixture containing **Boc-FDDP**, **Z-FEP**, and **Fmoc-FPP** exhibited the same trend, with W-NH_2_ again reacting most efficiently with **Fmoc-FPP** (Figures S73–S74). We next examined the mixture containing **Boc-FEP**, **Z-FNP**, and **Fmoc-FEP**. In this case, W-NH_2_ was incorporated preferentially into **Z-FNP** rather than **Fmoc-FEP** (Figures S75 and S76).

Together these experiments reveal that selective peptide coupling can be directed by tuning structural features of the phosphate ester, which modulate supramolecular recognition. Embedding structural elements within the leaving group transforms the acyl phosphate from an electrophile into an active design element that guides selective amide bond formation in multicomponent mixtures.

### Elongation and Phosphoryl Exchange Pathways of *N*-Terminus Free Aminoacyl Phosphates

Having established how the phosphate tail influences self-assembly and reactivity in the *N*-terminus-protected systems, we next turned to the behavior of *N*-terminus-free aminoacyl phosphates to address two distinct questions. First, we assessed whether different phosphate leaving groups affect peptide elongation when the amino group is unprotected. Second, we investigated whether the phosphate moiety can undergo in situ reconfiguration through phosphoryl-exchange processes that generate new acyl phosphates. The latter question is motivated by biological reactive intermediates, such as aminoacyl adenylates which participate in peptide-bond formation and various metabolic pathways. Inspired by these processes, we examined whether aminoacyl phosphate esters can undergo exchange reactions with orthophosphate, pyrophosphate, or nucleotide phosphates under aqueous conditions. Thus, we studied phosphoryl exchange in a series of aminoacyl phosphate esters that share a phenylalanine backbone but contain different C-terminal phosphate esters­(ethyl, phenyl, and dodecyl), as shown in Scheme 2. **FEP** and **FPP** remained soluble in aqueous buffer, while **FDDP** formed turbid solutions. Transmission electron microscopy and confocal imaging confirmed the formation of spherical aggregates in **FDDP** samples (Figure 5a). To address our first question: how different C-terminal substituents influence peptide oligomerization, we prepared samples using 10 mM **FEP**, **FPP**, and **FDDP** in MOPS buffer (pH 8.0). In all cases, we observed no significant differences in their oligomerization behavior (Figures S77–S82).^12^ We then investigated whether in situ phosphoryl exchange could influence the reaction outcome. Thus, we tested phosphoryl exchange with orthophosphoric acid. 10 mM **FEP** or **FDDP** was incubated with varying concentrations of orthophosphoric acid in PBS buffer (0.2–1.2 M at pH 8.0). Time-dependent UPLC analysis revealed that **FEP** readily underwent phosphoryl exchange to form phenylalanine orthophosphate (**FPi**), observed as a distinct peak at 2.5 min in the chromatogram (Figures 5b and S83–S85). The extent of **FPi** formation increased with buffer strength, reaching a maximum conversion of 39.5% in 1.2 M PBS (Figure S86). This transformation directly altered the amino acid oligomerization pathway. As **FEP** was converted to **FPi**, oligomer yields decreased while hydrolysis became dominant (Figure 5c). This behavior likely arises from the reduced electrophilicity of **FPi**, as its extra negative charge makes it less reactive toward nucleophiles. While **FEP** was fully consumed within 15 min, **FPi** reacted only slowly and a residual peak remained over the same period (Figure 5b), highlighting its greater kinetic stability.

**Figure 5:**
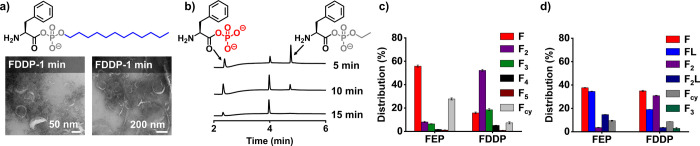
(a) TEM images of **FDDP** (10 mM) performed immediately after dissolution in 0.2 M PBS buffer (pH 8.0). (b) Time-dependent UPLC chromatograms showing phosphoryl exchange of **FEP** (10 mM) into **FPi**, monitored at 5, 10, and 15 min in 0.2 M PBS buffer (pH-8.0). (c) Oligomer distribution from 10 mM **FEP** and 10 mM **FDDP** in 0.2 M PBS buffer (pH 8.0). (d) Oligomer distribution from reactions of 10 mM **FEP** or 10 mM **FDDP** with 10 mM L-NH_2_ in 0.6 M PBS buffer (pH 8.0). Error bars represent the standard deviation of three independent experiments. FEP oligomers were measured after 1 h and FDDP oligomers were measured after 24 h.

**Scheme 2:**
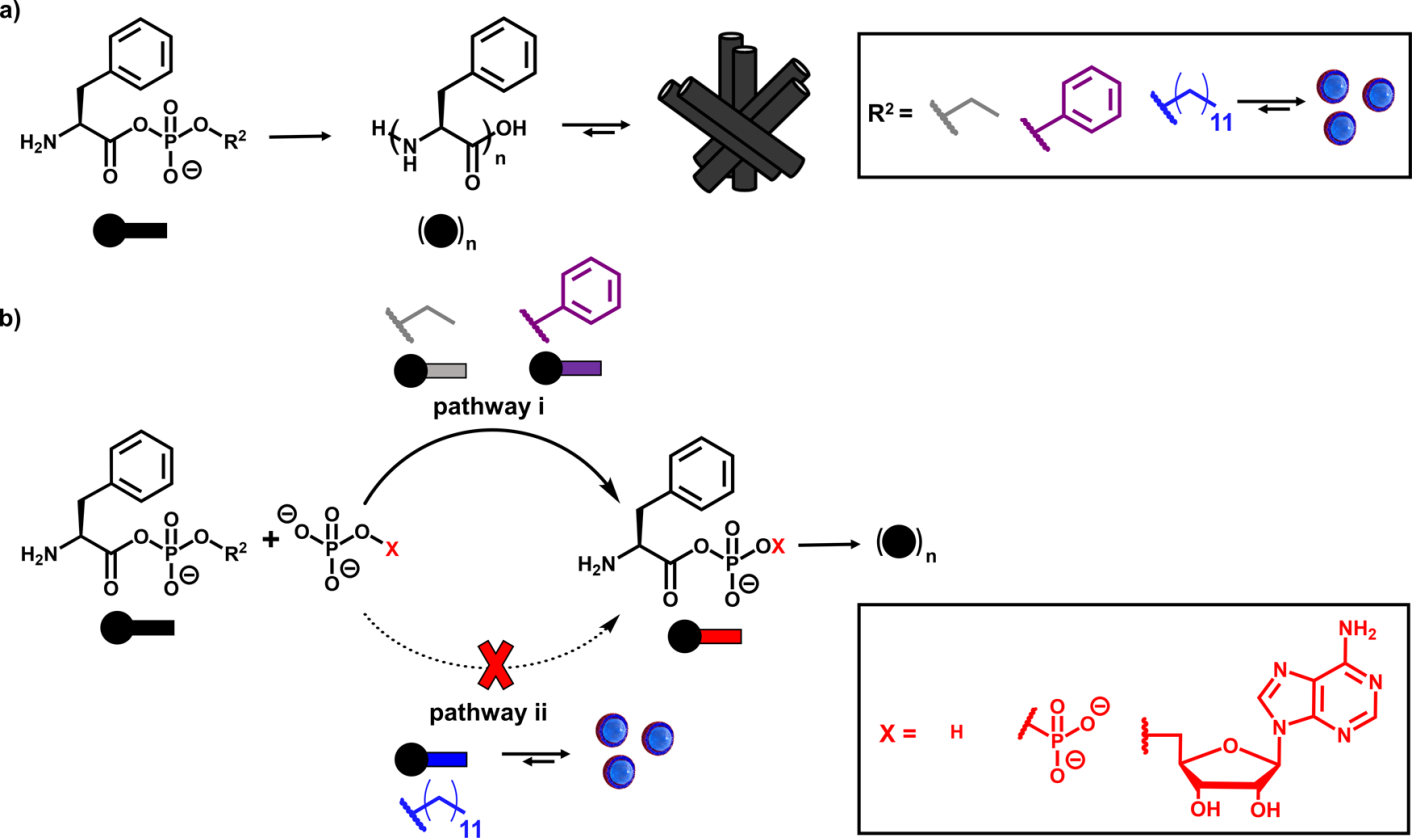
(a) Peptide Elongation from *N*-Terminus-Free Aminoacyl Phosphates Bearing Different Phosphate Leaving Groups (R^2^) and (b) Competing Pathways with *N*-Terminus-Free Aminoacyl Phosphates. Pathway i: the Acyl Phosphate Undergoes Phosphoryl Exchange with External Phosphate Species (Orthophosphate, Pyrophosphate, AMP), Generating a New Aminoacyl Phosphate with a Reconfigured Leaving Group. Pathway ii: When the Original Acyl Phosphate Aggregates, the Phosphate Moiety Becomes Shielded, Suppressing Exchange. Structural Variations in R^2^ and in the Exchanging Phosphate (X) Dictate Which Pathway Dominates

We then shifted our focus to the assembling species, **FDDP**, for which no **FPi** formation was detected, even at high PBS concentrations (Figure S87). Aggregation likely sequesters **FDDP** from the aqueous phase and restricts access to the phosphates (Figure 5a). This preservation of reactivity favors oligomerization over hydrolysis, resulting in a product distribution distinct from **FEP** (Figure 5c). These findings illustrate how aggregation can shield reactive intermediates from side reactions, maintaining the reaction pathway. We further extended this approach to N-protected aminoacyl phosphate esters. Phosphoryl exchange experiments with **Boc-FEP** and **Boc-FDDP** revealed that **Boc-FEP** undergoes only minimal exchange in 1.2 M PBS (Figures S88–S90). In contrast, no exchange was observed for **Boc-FDDP** (Figure S91), indicating that its assembled state effectively suppresses phosphoryl exchange. To test whether phosphoryl exchange extends to biologically relevant phosphates, we performed experiments using adenosine monophosphate (AMP) and phenylalanine ethyl phosphate. Incubation of **FEP** (10 mM) with AMP (100 mM) in HEPES buffer (pH 8.0) led to formation of **F-AMP**, **F**_**2**_ **-AMP**, and **F**_**3**_ **-AMP** species, confirmed by UPLC-MS (Figures S92 and S93). Moreover, incubation of **FEP** (10 mM) with pyrophosphoric acid (100 mM) in HEPES buffer (pH 8.0) produced **FPPi** (Figures S94–S96), albeit to a lesser extent compared to monophosphate. In contrast, **FDDP** showed no evidence of phosphoryl exchange, confirming that assembling acyl phosphates resisted transformation by both inorganic and nucleotide-based phosphates. In addition to **FEP**, **FPP** also underwent phosphoryl exchange (Figures S97–S99).

To probe how this process influences product distribution, we compared homo and heterocoupling in mixtures of **FEP** or **FDDP** with L-NH_2_ in 0.6 M PBS buffer (Figures S100–S101). For **FEP**, heterocoupling was favored since nucleophiles remained accessible in solution, whereas for **FDDP**, which resisted exchange, homo-oligomers dominated, as assembly biased the system toward self-coupling (Figure 5d).

Together, these results show that in situ reconfiguration of the phosphate group provides an alternative to synthetic modifications for tuning reactivity. In solution, acyl phosphates undergo phosphoryl exchange that lowers electrophilicity and promotes hydrolysis, whereas in self-assembled systems, acyl phosphates are shielded from exchange, stabilizing the reactive intermediate and favoring oligomerization.

## Conclusions

In this work, we focused on the role of the phosphate leaving group in regulating peptide bond formation and supramolecular assembly from aminoacyl phosphate esters. We demonstrated that structural variations in the leaving group, ranging from ethyl and phenyl to naphthyl and dodecyl phosphate profoundly influence the reactivity and the self-assembly propensity of the activated species. In general, aminoacyl phosphates bearing aromatic or hydrophobic aliphatic groups in the “phosphate tail”self-assembled into compartments that controlled reactivity and redirected covalent transformation pathways. While peptide products were the same, the pathways that produced them differed, driven by aminoacyl phosphate self-assembly and leaving group coassembly. These differences persisted beyond activation, highlighting how subtle changes in reactive intermediates can steer supramolecular organization and material properties. We showed that specific combinations of aminoacyl phosphates and amino acid nucleophiles produce sequence-selective amides, with selectivity arising from microenvironments created by certain phosphates that promote noncovalent interactions. In addition, we showed that soluble aminoacyl phosphates undergo exchange with orthophosphate, pyrophosphate, and AMP to form acyl monophosphates, diphosphates and acyl adenylates, intermediates that divert reactivity and activate new pathways. By contrast, self-assembling phosphates resist exchange, maintaining reactivity and supporting amino acid oligomerization. Together, these findings highlight phosphoryl exchange as a control process that determines the outcome of activated intermediates according to the leaving group’s structure and assembly. More broadly, our study establishes the leaving group in acyl phosphate esters as a tunable design element that channels reactivity and assembly dynamics. Embedding structural features directly within the activating group offers a previously overlooked strategy to control aqueous amide bond formation and supramolecular assembly in peptide systems chemistry. Looking ahead, we will explore whether recognition elements embedded in activating groups can be harnessed to mimic aspects of biological peptide synthesis, where activation, assembly and sequence orthogonality are intricately coupled.

## Supplementary Material







## Data Availability

The data that support the findings of this study are available from the corresponding author upon reasonable request.

## Notes

Boc-FDDP with L-NH_2_ (AVI)
Materials and Methods, NMR spectra, additional UPLC chromatograms, LC-MS analyses, coupling yields, confocal and transmission electron microscopy images and references (PDF)
Boc-FDDP with R-NH_2_ (AVI)
