# Supplementary material for: Tailored Phosphate Leaving Groups Direct Pathway-Dependent Self-Assembly
Source: J Am Chem Soc. 2026 Jan 22;148(8):8200–12. doi: 10.1021/jacs.5c17237 (PMC12964403; doi:10.1021/jacs.5c17237)
Supplement: Supplementary file 2 [file ja5c17237_si_002.pdf]

Supporting Information for:

Tailored Phosphate Leaving Groups Direct Pathway-Dependent Self-Assembly

Arti Sharma[1,2], Kun Dai[3], Mahesh D. Pol[2,3], Anatoli Ioanna Katirtzidi Papadopoulou[3], Thejus Pramod[2,3], Ralf Thomann[1,4], Yi Thomann[1,4], and Charalampos G. Pappas[1,2,3*]

[1]Freiburg Center for Interactive Materials and Bioinspired Technologies (FIT), University of Freiburg, Georges-Köhler-Allee 105, 79110, Freiburg, Germany. [2]Institute of Organic Chemistry, University of Freiburg, Albertstrasse 21, 79104, Freiburg, Germany. [3]DFG Cluster of Excellence *liv*MatS @FIT – Freiburg Center for Interactive Materials and Bioinspired Technologies, University of Freiburg, Georges-Köhler-Allee 105, 79110, Freiburg, Germany. [4]Freiburg Materials Research Center (FMF), University of Freiburg, Stefan-Meier-Strasse 21, 79104, Freiburg, Germany.

Contents

1. Material and methods 3

2. Synthesis and characterization of aminoacyl phosphate esters (Boc-FEP, Boc-FPP, Boc-FNP, Boc-FDDP, Cbz-FEP, Cbz-FPP, Cbz-FNP, Cbz-FDDP, Fmoc-FEP, Fmoc-FPP, FPP, FEP, FDDP) 8

2.1 General synthesis 8

3. Supporting figures 10

4 Characterization of compounds 80

4.1 Characterization of Boc/Cbz/Fmoc-aminoacyl phosphate esters by NMR 80

4.2 Characterization of Boc/Cbz/Fmoc-aminoacyl phosphate esters by mass 102

5 References 106

1. Material and methods

Materials:
All reagents were purchased from Sigma-Aldrich and Carl Roth and used without any further purification unless otherwise indicated. N-terminus protected and free amino acids were purchased from Carbolution ABCR and VWR. Aluminium metal plates precoated with silica gel 60 matrix, 0.25 mm or 0.5 mm were utilised for thin-layer chromatography (TLC). Visualisation of the developed TLC plate was performed by irradiation with UV light. Preparative RP-MPLC was performed using an automated Interchim- puriFlash® system.

Sample preparation. Amides were dissolved in 0.6 M borate buffer at pH 9.1 and transferred to a new vial containing N-terminus protected and N-terminus free aminoacyl phosphate esters followed by vortexing and sonication. The concentration of the phosphate esters used in all samples was 10 mM. For phosphoryl exchange experiments, aminoacyl phosphate esters were dissolved in 0.2M, 0.6M and 1.2 M PBS buffer at pH 8.0. Control experiments of oligomerization reactions of aminoacyl phosphate esters were performed in 0.2M, 0.6M and 1.2 M MOPS buffer at pH 8.0.

Methods:
UPLC analysis. UPLC analyses were performed on a Waters Acquity UPLC H-Class Bio system, equipped with a photodiode array detector at a detection wavelength of 214 nm. Samples were injected on an Acquity UPLC CSH-C18 column, 130Å,1.7μm (150 × 2.1 mm), and Kinetex LC column 100Å, 2.6μm (150 × 2.1 mm), and using UPLC-MS grade water eluent (A) and UPLC-MS grade acetonitrile eluent (B), which contained 0.1% trifluoroacetic acid as the modifier. A flow rate of 0.3 ml min^{-1} and a column temperature of 35 °C were applied. For all samples, vortex (15 seconds) and sonication (15 seconds) were performed prior to UPLC injection to ensure a homogeneous phase. Samples were prepared by taking 10 μl from the reaction vial and diluting (100 times) into H_2O or H_2O:ACN (1:1) and H_2O:THF mixture (1:1). Vortex and sonication (for 15 seconds) was furthermore applied after dilution and prior to UPLC injection. The libraries which formed precipitation or insoluble aggregates were first dissolved in 100% organic solvent (ACN) in order to solubilize all the components before injecting into UPLC.

UPLC methods:

A)

Time (min)	Eluent-A (Water) %	Eluent-B (ACN) %
0	95	5
15	30	70
16	10	90
17	10	90
17.5	95	5
20	95	5

B)

Time (min)	Eluent-A (Water) %	Eluent-B (ACN) %
0	95	5
3	50	50
15	10	90
16	10	90
17	10	90
17.5	95	5
20	95	5

C)

Time (min)	Eluent-A (Water) %	Eluent-B (ACN) %
0	95	5
15	30	70
20	30	70
21	10	90
22	10	90
22.5	95	5
25	95	5

D)

Time (min)	Eluent-A (Water) %	Eluent-B (ACN) %
0	95	5
15	10	90
16	10	90
17	10	90
17.5	95	5
20	95	5

Method A) was used for the libraries of Boc/Z-protected aminoacyl phosphate esters with all amides and for **FEP**, **FPP** and **FDDP**.

Method B) was used for the libraries of Fmoc-protected aminoacyl phosphate esters with all amides.

Method C) was used for the individual and mixed libraries of N-protected aminoacyl phosphate esters (Boc/Z/Fmoc).

For the mixing experiments, multiple coupling products can form, each with distinct absorbance, making peak area comparisons incorrect. To enable quantitative analysis, we calibrated the N-protected acyl phosphates used in the mixtures of Boc-F, Z-F, and Fmoc-F. Aromatic amino acid used in the mixture experiments, for example Trp (W) amide is also used for the calibration. These calibration data were then used to derive correction factors that normalize the peak areas across all coupling products.

For example, the peak areas of Boc-F and Z-F-derived species were corrected by multiplying them with factors obtained from the ratio of the corresponding Fmoc-FW derived peak area to that of the Boc-F or Z-F species. These corrections allow us to compare the areas on a consistent and quantitative basis.

The following method we used to calculate the correction factor:

Correction Factor (X):
X = (Area of Fmoc-F species) / (Area of Boc-F/ Z-F species)

Adjusting the area of Boc-F/ Z-F species:

Adjusted Area of Boc-F/ Z-F = X * (Area of Boc-F/ Z-F species)

The resulted calibrated graph from Z-F-OH, Fmoc-F-OH, Boc-F-OH, W-NH_2 are shown below:

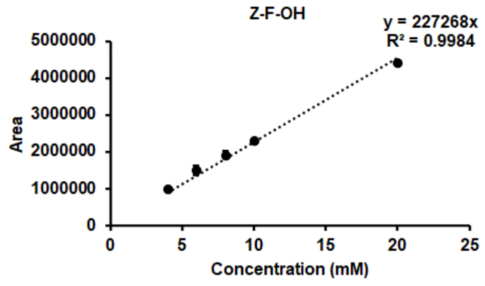

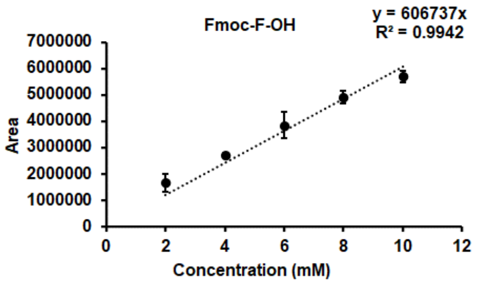

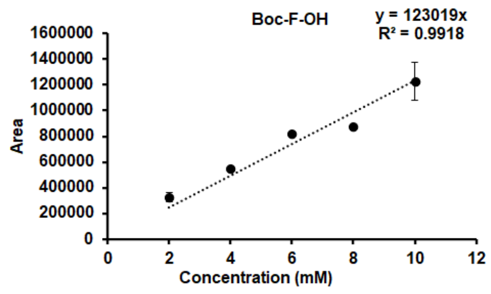

UPLC-MS

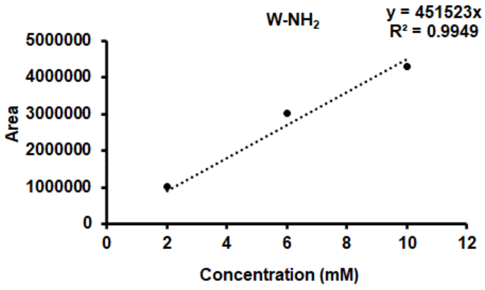

analysis. Ultra-Performance Liquid Chromatography-Mass Spectrometry (UPLC-MS) experiments were performed on an Agilent 6546 LC/Q-TOF equipped with an infinity 1290 II in the LC section. We used the same UPLC column, as described in the UPLC analysis section above. The Q-TOF was equipped with a dual AJS ESI source. The experiments were conducted at a VCap voltage of 4000 V, a sheath gas temperature of 300°C and a fragmentor voltage of 120 V. An internal reference was used.

NMR. The ^{1}H NMR spectra were recorded on a Bruker Avance Neo 400 MHz and Bruker Avance 300 MHz with broadband cryoprobe Prodigy. The ^{31}P NMR spectra were recorded on Bruker 122 MHz and 162 MHz spectrometers using ^{1}H-broad band decoupling in the indicated deuterated solvent. Chemical shifts were reports as delta values from standard peaks.

Rheology. Rheological measurements were carried out with an Anton Paar MCR 302 rheometer at 25 °C, using a 25 mm cone-plate geometry (CP25-1, Anton Paar) and a measuring gap of 0.047 mm. Samples were prepared as previously mentioned (sample preparation section) and placed on the bottom plate. For all the peptide samples, storage moduli (G′) and loss moduli (G″) were measured at a strain amplitude of γ = 5.0% and a frequency of ω = 10.0 rad/s. A solvent trap was placed around the sample holder to avoid evaporation.

Turbidity. Turbidity measurements were carried out at 25°C on a Microplate Spectrophotometer (Tecan Safire). Measurements were performed in a 96-well plate. Absorbance was measured at 600 nm.

Cryo-transmission electron microscopy (Cryo-TEM)

Cryo-EM was used to visualize the morphology of Boc- and Z- protected amino acyl phosphate esters in their native state. A C-flat holey carbon grid with standard 20 nm carbon thickness, 2.0 µm hole diameter, and 300 mesh Cu grid (CF-2/4-3Cu from Protochips Inc., North Carolina, USA) was hydrophilized by 2 min of glow discharge using air and a remote source at 15 W in a Tergeo EM Plasma Cleaner (PIE Scientific LLC, California, USA). Sample at 10 mM concentration, was first dissolved in the 0.6 M borate buffer, pH 9.1, and then applied to the carbon grid at 22 °C. The sample was vitrified by automated blotting and plunge freezing with an FEI Vitrobot Mark IV (Thermo Fisher Scientific Inc.) using liquid ethane as the cryogen. Cryo-EM image acquisition: The vitrified specimen was transferred to an FEI TALOS L120C electron microscope (Thermo Fisher Scientific Inc.) with an acceleration voltage of 120 kV using a 626 single tilt liquid nitrogen cryo-transfer holder (Gatan Inc.). Images were captured using a CETA camera.

Confocal Microscopy. Imaging was performed on a Zeiss LSM 710 confocal microscope system using a 63x oil immersion objective. Samples were prepared as described above and transferred into micro-well plates (ibidi, µ-Slide 8 well Bioinert). 1-3 µm Nile red and Alexa Fluor (AF488) was added as fluorescent dye. Samples were excited with 561 nm and were imaged at 575-630 nm.

Scanning Electron Microscopy (SEM). Peptide assemblies were deposited onto p-type silicon wafers and sputter-coated with a 2-3 nm Pt layer prior to imaging. Scanning electron microscopy (SEM) was carried out on a FEI Quanta FEG 250 DualBeam (FIB-SEM) equipped with an Everhart-Thornley detector (ETD). Images were acquired in secondary electron (SE) mode at an accelerating voltage of 5 kV, a beam current of 0.20 nA, a working distance of 7.2 mm, and 0° tilt. Horizontal field widths (HFW) ranged from 10-50 µm depending on magnification. Dwell time per pixel was 100 ns.

Dynamic light scattering (DLS)

DLS measurements were recorded using a Malvern Zetasizer Nano ZSP instrument. 10 mM **Boc-FNP** was dissolved in 0.6 M borate buffer, pH 9.1, and was immediately measured after dissolving the acyl phosphate at 25 °C.

2. Synthesis and characterization of aminoacyl phosphate esters (Boc-FEP, Boc-FPP, Boc-FNP, Boc-FDDP, Cbz-FEP, Cbz-FPP, Cbz-FNP, Cbz-FDDP, Fmoc-FEP, Fmoc-FPP, FPP, FEP, FDDP)

2.1 General synthesis

Synthesis of aminoacyl phosphate esters was modified from a previously reported procedure.[1]

I. General procedure for the synthesis of Bis(tetraethylammonium salt) ethyl/phenyl/napthyl/dodecyl phosphate:

2-Naphthol and dodecanol (1 eq.) were dissolved in dry diethyl ether (20 vol) under N_2 condition. $POCl_3$ (1 eq.) was added at room temperature. The mixture was cooled to –78°C, and triethylamine (1 eq.) was added dropwise. The reaction was kept at –78°C for 5 min, then warmed to room temperature and stirred for 2 h. The mixture was filtered under nitrogen, and the filtrate was concentrated by rotary evaporation.

Ethyl/phenyl/napthyl/dodecyl phosphorodichloridate was added dropwise to a ten-fold excess of distilled water in an ice-cooled round-bottom flask. After stirring the reaction mixture for one-hour, hydrochloric acid was generated as a by-product and was removed by rotary evaporation. This yielded ethyl phosphoric acid as a clear oil. The resulting ethyl phosphoric acid was neutralized with tetraethylammonium hydroxide (35% w/w solution, 2.0 eq.), resulting in a white paste. The paste was obtained by freeze-drying and used for coupling reactions without further purification.

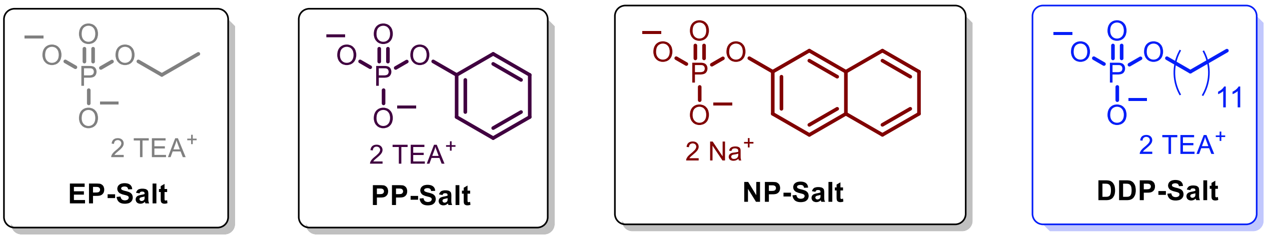

EP Salt: ^{1}H NMR (300 MHz, D_2O) δ (ppm) 3.86 (p, *J* = 7.1 Hz, 2H), 3.23 (q, *J* = 7.3 Hz, 16H), 1.34 – 1.14 (m, 27H); **^{31}P NMR (122 MHz, D_2O)** *δ* (ppm) 1.07.

PP Salt: ^{1}H NMR (300 MHz, D_2O) δ (ppm) 7.49 – 7.27 (m, 2H), 7.26 – 7.02 (m, 3H), 3.21 (q, *J* = 7.3 Hz, 16H), 1.29 – 1.18 (m, 24H); **^{31}P NMR (122 MHz, D_2O)** δ (ppm) -1.78.

NP Salt: **^{1}H NMR (300 MHz, D_2O)** δ (ppm) 8.44 – 8.31 (m, 1H), 7.98 – 7.90 (m, 1H), 7.69 – 7.48 (m, 5H); **^{31}P NMR (122 MHz, D_2O)** *δ* (ppm) 0.50.

DDP Salt: ^{1}H NMR (300 MHz, $CDCl_3$) δ (ppm) 3.79 (q, *J* = 6.7 Hz, 2H), 3.43 (q, *J* = 7.3 Hz, 16H), 1.53 (t, *J* = 7.1 Hz, 2H), 1.38 – 1.30 (m, 24H), 1.29 – 1.11 (m, 16H), 0.84 (t, *J* = 6.6 Hz, 3H); **^{31}P NMR (122 MHz, D_2O)** *δ* (ppm) 1.36.

II. General procedure for the synthesis of Boc/Cbz/Fmoc-aminoacyl phosphate esters:

N-Boc-L-amino acid (3.75 mmol, 1.0 eq) was dissolved in dry DCM (30 mL). DCC (3.75 mmol, 1.0 eq) was added to this solution and then stirred for 3-5 minutes which resulted in a white precipitate. Bis(tetraethylammonium salt) ethyl phosphate (3.75 mmol, 1.2 eq), pre-dissolved in dry DCM (10 mL), was added, and stirred for 1 hour at room temperature. The progress of the reaction was monitored using TLC analysis [MeOH:DCM (10:90)]. The reaction mixture was then filtered to remove the white DCU precipitate. The filtrate was evaporated under reduced pressure and the residue was purified by automated RP flash column chromatography (C18-AQ, H_2O/ACN). Fractions containing product were collected and lyophilised to obtain the aminoacyl phosphate esters as TEA salt.

III. General procedure for ion exchange:

The TEA salt of Boc/Cbz/Fmoc-aminoacyl phosphate esters was dissolved in a minimum amount of water and then passed through a column which contained Dowex®50WX8 ion exchange resin loaded with the Na^+ counter ion. Collected water solution was then frozen in liquid nitrogen and lyophilized to dryness. This process yields sodium salt of Boc/Cbz/Fmoc-aminoacyl phosphate esters as a white hygroscopic solid.

IV. General procedure for the Boc deprotection:

The TEA salt of N-t-Boc aminoacyl ethyl phosphate was dissolved in a minimum amount of trifluoroacetic acid (TFA). After 10-15 minutes, the TFA was removed by rotary evaporation (30°C) and vacuum pumping. The resulting oil was dissolved in cold acetone and diethyl ether and the formed precipitate was collected by vacuum filtration and washed several times with acetone and diethyl ether. After drying under nitrogen for 10 minutes, the aminoacyl ethyl phosphate was isolated as a solid.

3. Supporting figures

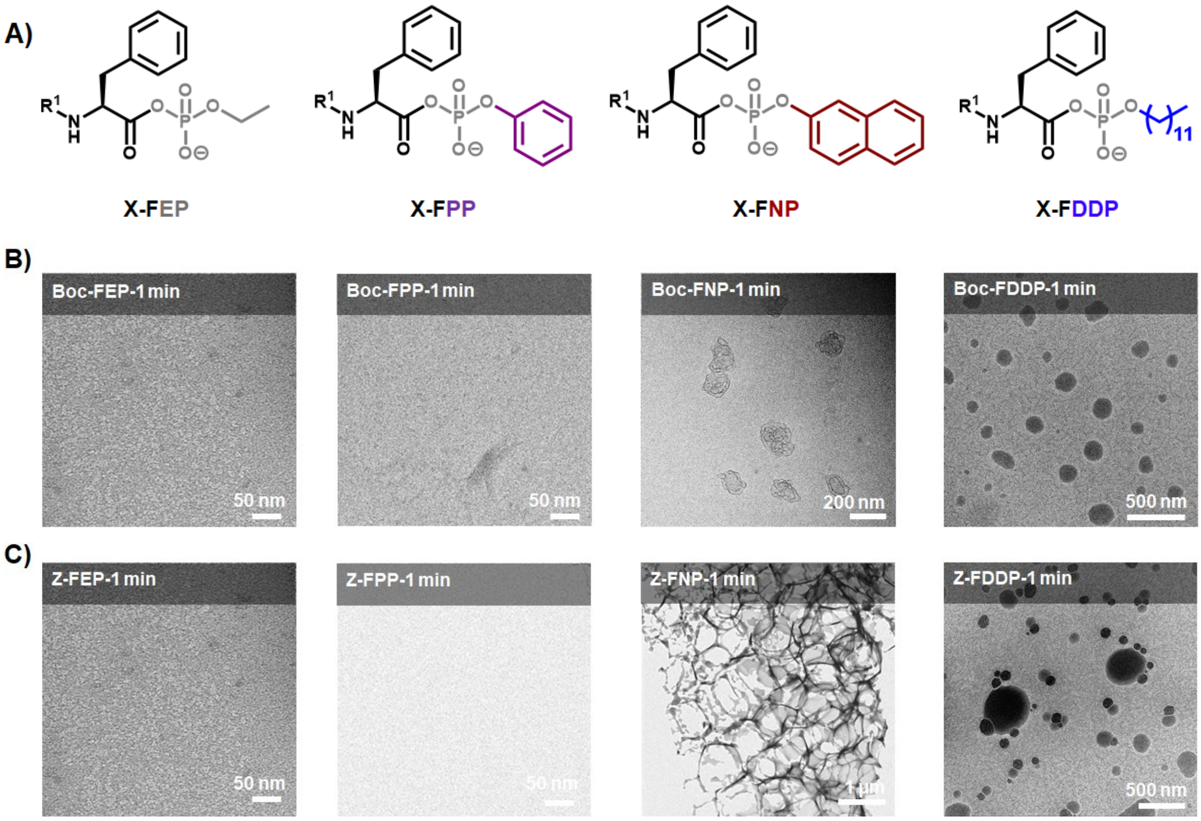

Figure S1. A) Chemical structures of Boc/Z- protected amino acyl phosphate esters, **B)** and **C)** Cryo-transmission electron microscopy images (left to right) of 10 mM **Boc/Z-FEP**, **Boc/Z-FPP**, **Boc/Z-FNP**, **Boc/Z-FDDP**. Images were acquired immediately after dissolving the acyl phosphates in 0.6 M borate buffer, pH 9.1.

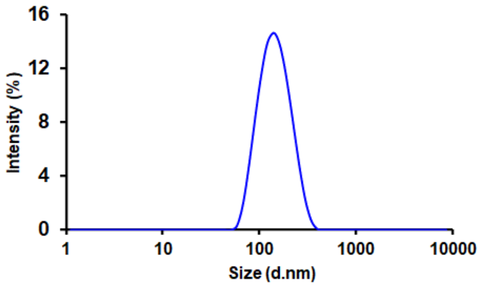

Figure S2. Size distribution of 10 mM **Boc-FNP** measured by dynamic light scattering (DLS), performed immediately after dissolution in 0.6 M borate buffer, pH 9.1.

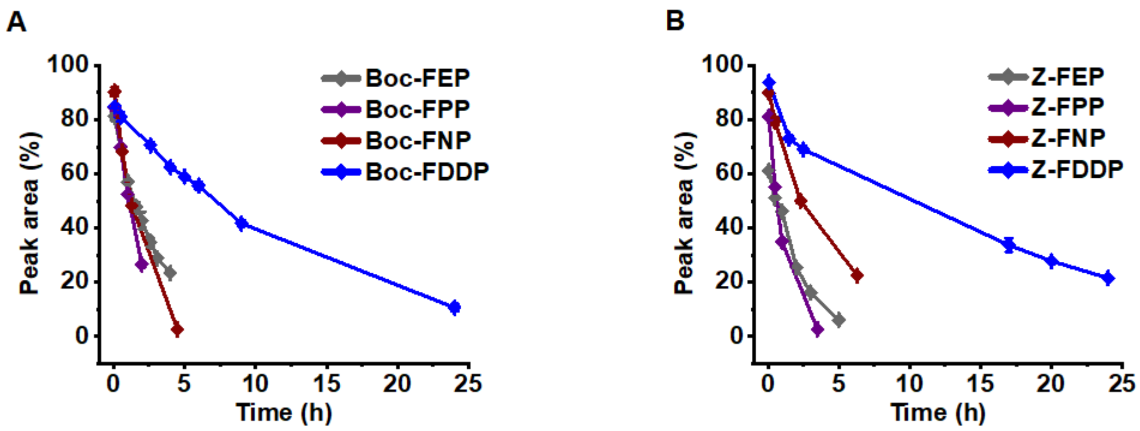

Figure S3. Hydrolysis profiles of 10 mM **A)** Boc-protected, **B)** Z-protected aminoacyl phosphate esters in 0.6 M borate buffer, pH 9.1. The half-lives were calculated by using the equation: $t_{1/2}$ = 0.693/k, assuming first-order kinetics. Error bars represent the standard deviation of three independent experiments. The half-life for Boc-FEP and Z-FEP is consistent with our previous report.[2,3]

Phosphate ester	$t_{1/2}$ (min)
Boc-FEP	130
Boc-FPP	78
Boc-FNP	86
Boc-FDDP	474

Phosphate ester	$t_{1/2}$ (min)
Z-FEP	87
Z-FPP	46
Z-FNP	186
Z-FDDP	744

Hydrolysis profile of Boc-protected amino acyl phosphate esters:

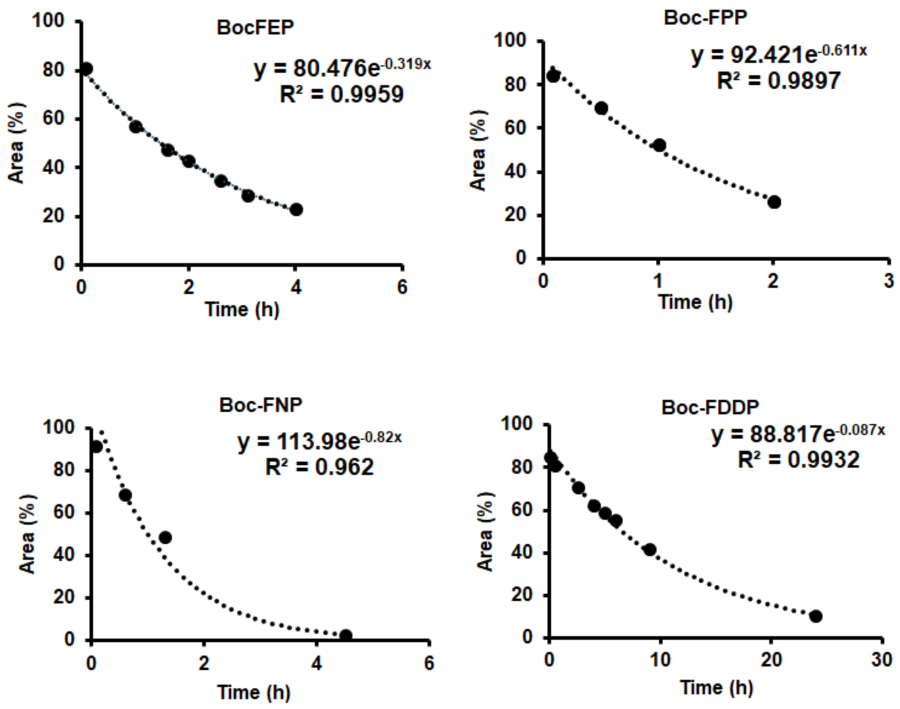

Hydrolysis profile of Z-protected amino acyl phosphate esters:

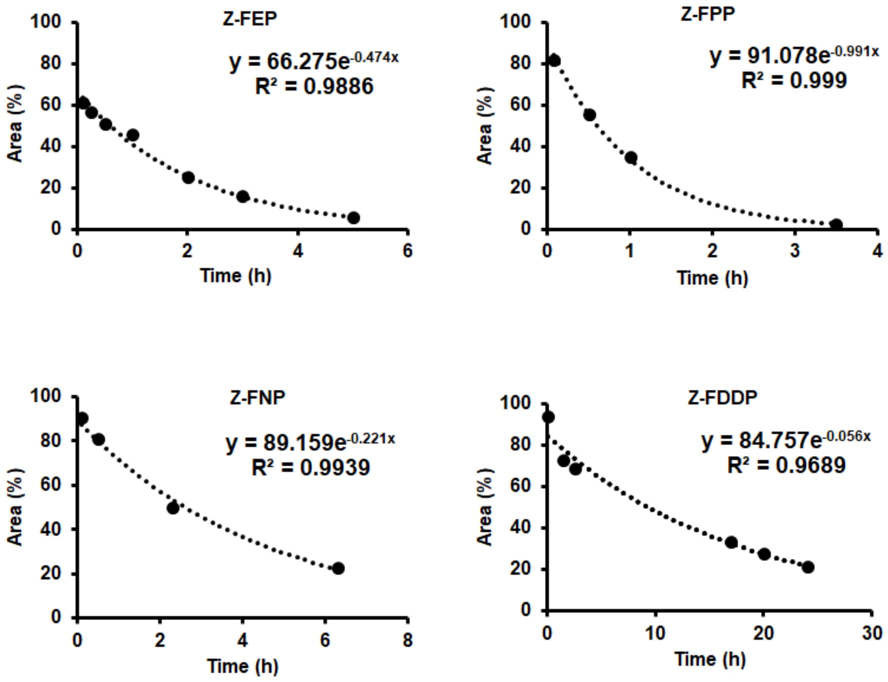

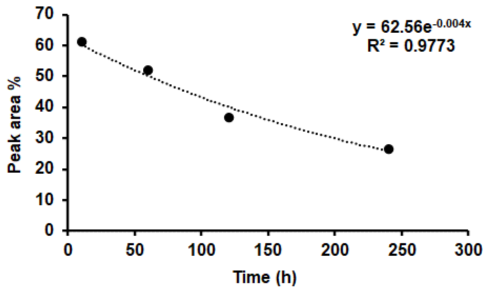

Figure S4. Hydrolysis of 10 mM **Boc-FDDP** in a co-solvent mixture (60% DMSO in 0.6 M borate buffer, pH 9.1). The half-life is calculated by using the equation: $t_{1/2} = 0.693/k$, assuming first-order kinetics. The calculated half-life is for 10 mM **Boc-FDDP** is 173 min.

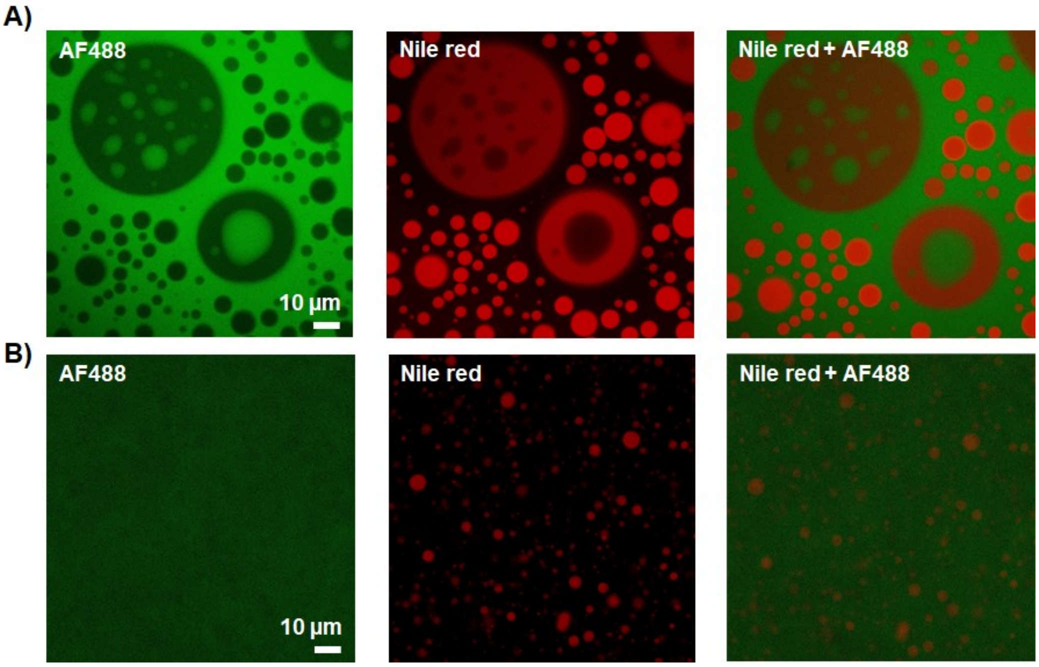

Figure S5. Confocal microscopy images of 10 mM **A) Z-FNP** and **B) Z-FDDP** incubated with AF488 (green) and Nile red (red) in 0.6 M borate buffer, pH 9.1. Scale bars are 10 µm.

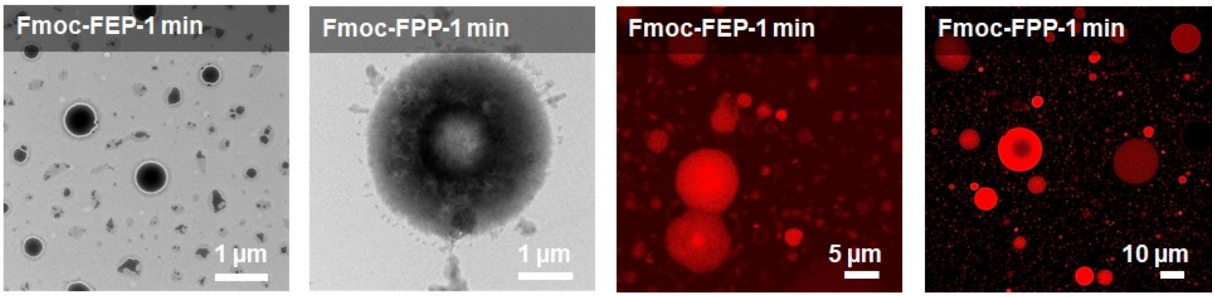

Figure S6. Transmission and confocal microscopy images (nile red staining) of 10 mM **Fmoc-FEP** and 10 mM **Fmoc-FPP**. Images were acquired immediately after dissolving the acyl phosphates in 0.6 M borate buffer, pH 9.1.

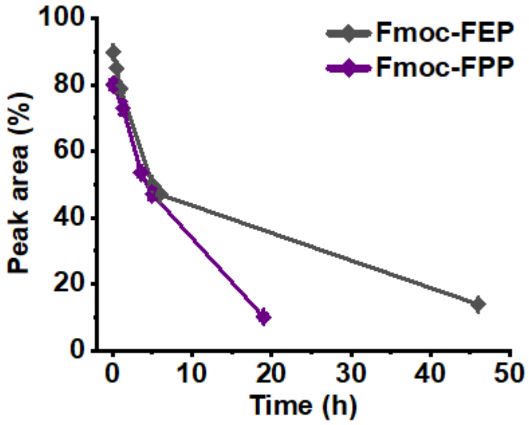

Figure S7. Hydrolysis profiles of 10 mM **Fmoc-FEP** and 10 mM **Fmoc-FPP** in 0.6 M borate buffer, pH 9.1. The half-life of **Fmoc-FEP** and **Fmoc-FPP** is 1123 min and 378 min, respectively. The half-lives were calculated by using the equation: $t_{1/2} = 0.693/k$, assuming first-order kinetics. The reduced quality of the fit for **Fmoc-FEP** may be attributed to the onset of self-assembly. Error bars represent the standard deviation of three independent experiments.

Hydrolysis profiles of Fmoc-protected amino acyl phosphate esters:

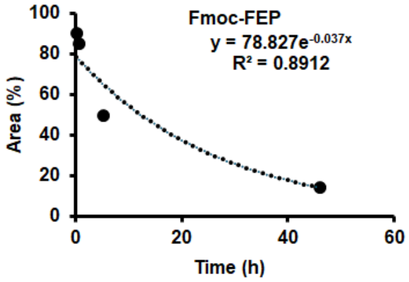

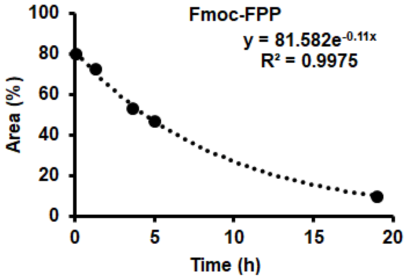

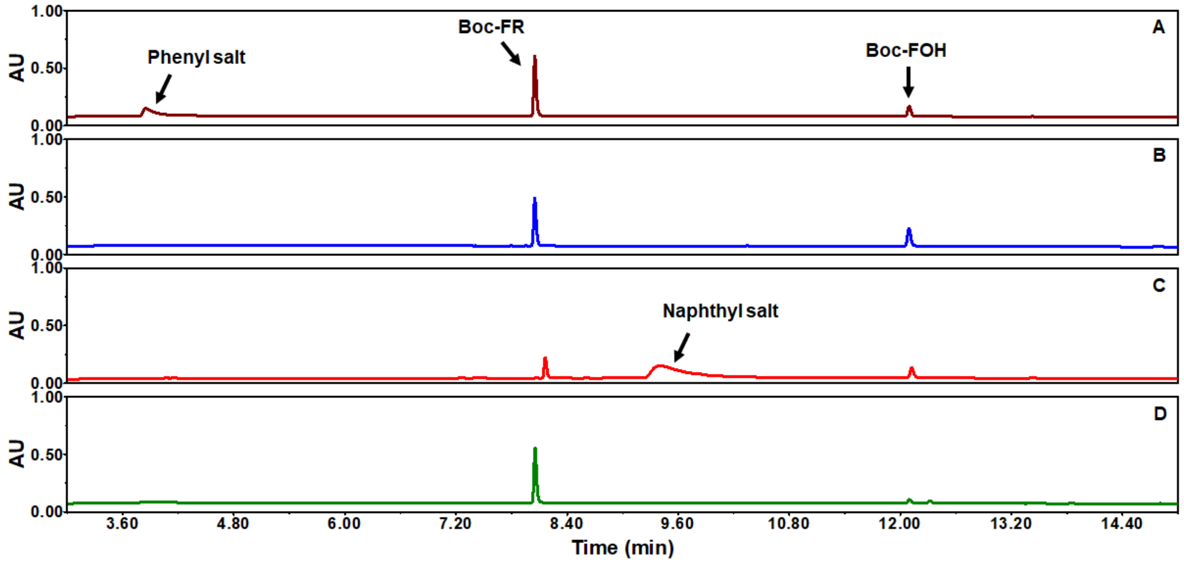

Figure S8. UPLC chromatograms of reactions between 10 mM R-NH_2 with **A)** 10 mM **Boc-FPP**, **B)** 10 mM **Boc-FEP**, **C)** 10 mM **Boc-FNP** and **D)** 10 mM **Boc-FDDP**, in 0.6 M borate buffer, pH 9.1. Measurements were taken after 1 hour.

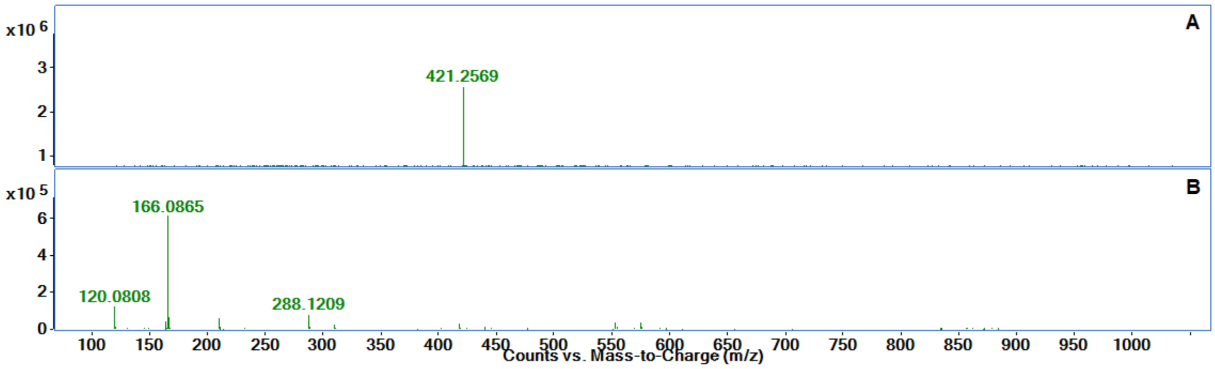

Figure S9. Mass spectra of peptide formed between 10 mM R-NH_2 and 10 mM Boc-protected amino acyl phosphate esters (**Boc-FEP**, **Boc-FPP**, **Boc-FNP** and **Boc-FDDP**) shown for: **A)** Boc-FR (retention time 8.11 min), **B)** Boc-F-OH (retention time 12.12 min), in Supporting Figure S8, obtained from the LC-MS analysis.

Calculated m/z $[M+H]^+$: **Boc-FR** 421.2528, **Boc-F-OH** 266.1387.

Observed m/z $[M+H]^+$: **Boc-FR** 421.2569, $[M+Na]^+$: **Boc-F-OH** 288.1209.

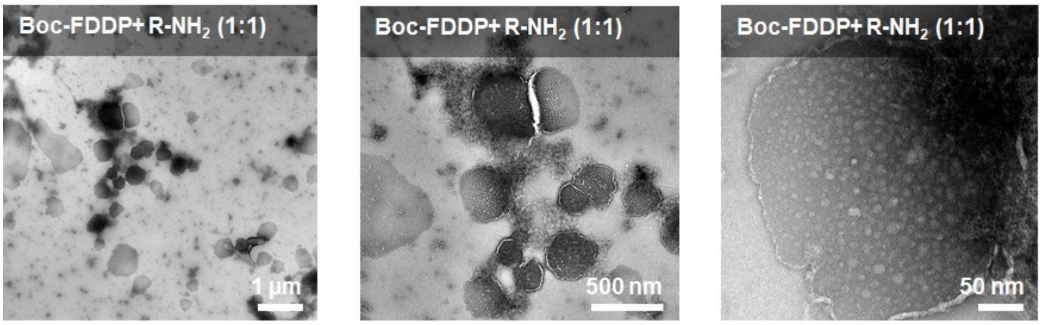

Figure S10. TEM images showing spherical aggregates obtained from reactions of 10 mM **Boc-FDDP** with 10 mM R–NH_2, in 0.6 M borate buffer, pH 9.1. Measurements were taken after 1 hour.

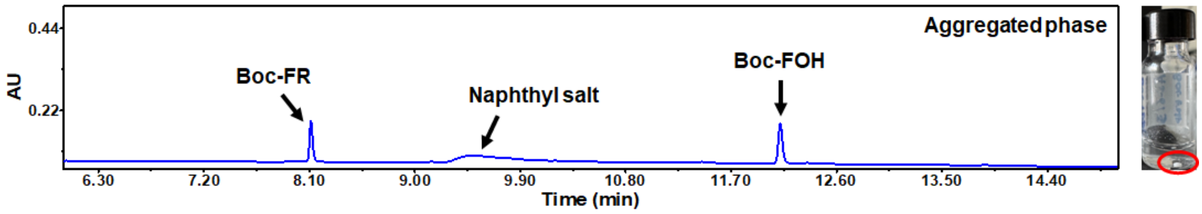

Figure S11. UPLC chromatogram of the aggregated phase obtained from the reaction of 10 mM R-NH_2 with 10 mM **Boc-FNP**, in 0.6 M borate buffer, pH 9.1.

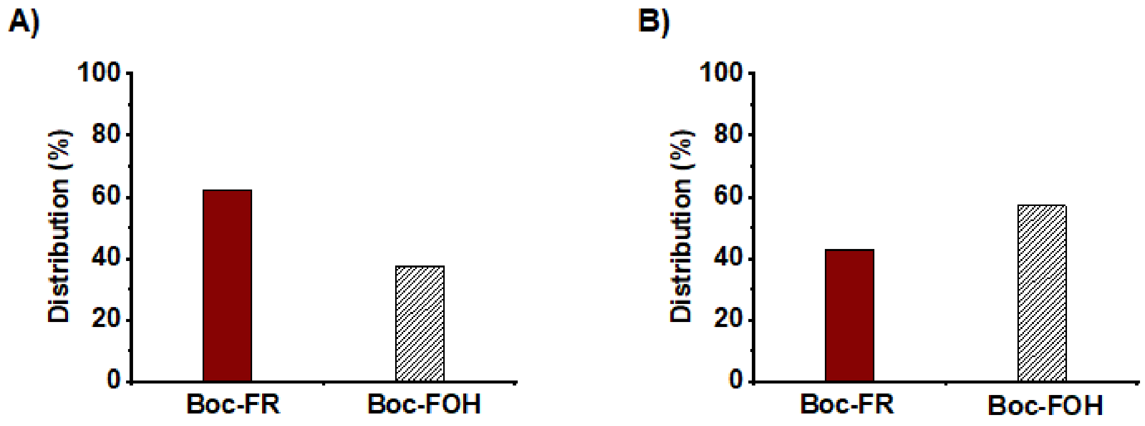

Figure S12. Bar graph showing distribution of peptide coupling in **A)** the aqueous phase and **B)** the aggregated phase (after centrifugation) from the reaction of 10 mM R-NH_2 with 10 mM **Boc-FNP**, in 0.6 M borate buffer, pH 9.1.

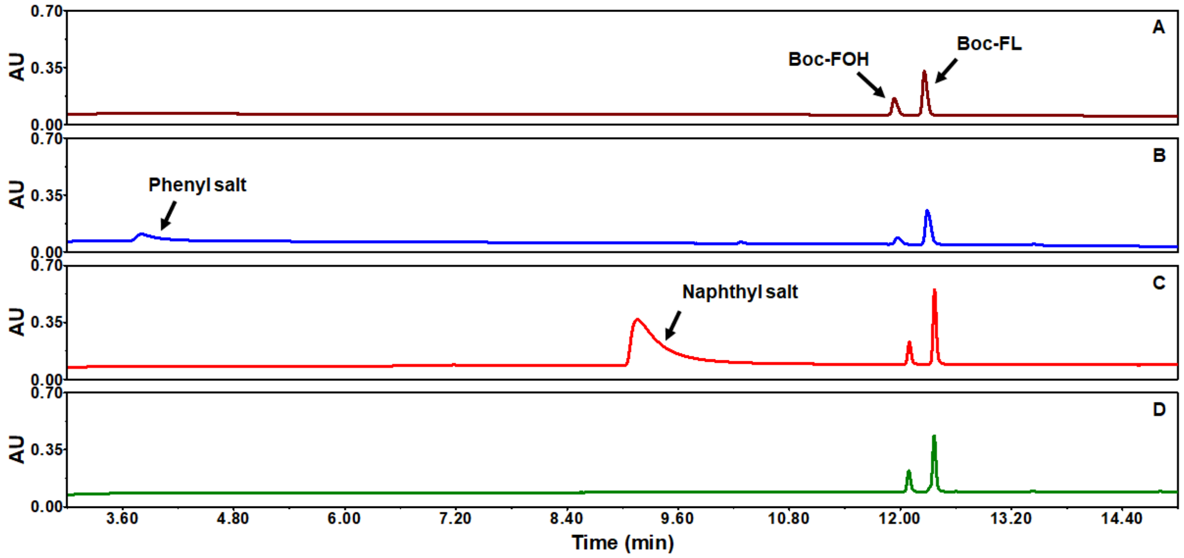

Figure S13. UPLC chromatograms of reactions between 10 mM L-NH_2 with **A)** 10 mM **Boc-FEP**, **B)** 10 mM **Boc-FPP**, **C)** 10 mM **Boc-FNP** and **D)** 10 mM **Boc-FDDP**, in 0.6 M borate buffer, pH 9.1. Measurements were taken after 1 hour.

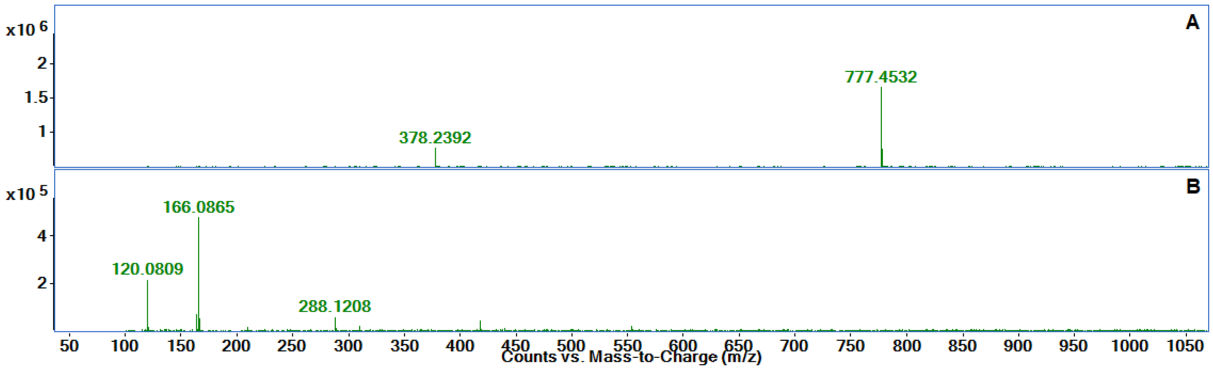

Figure S14. Mass spectra of peptide formed between 10 mM L-NH_2 and 10 mM Boc-protected amino acyl phosphate ester (**Boc-FEP**, **Boc-FPP**, **Boc-FNP** and **Boc-FDDP**) shown for: **A)** Boc-FL-NH_2 (retention time 12.36 min), **B)** Boc-F-OH (retention time 12.09 min), in Supporting Figure S13, obtained from the LC-MS analysis.
Calculated m/z $[M+H]^+$: **Boc-FL-NH_2** 378.2387, **Boc-F-OH** 266.1387.
Observed m/z $[M+H]^+$: **Boc-FL-NH_2** 378.2392, $[M+Na]^+$: **Boc-F-OH** 288.1208.

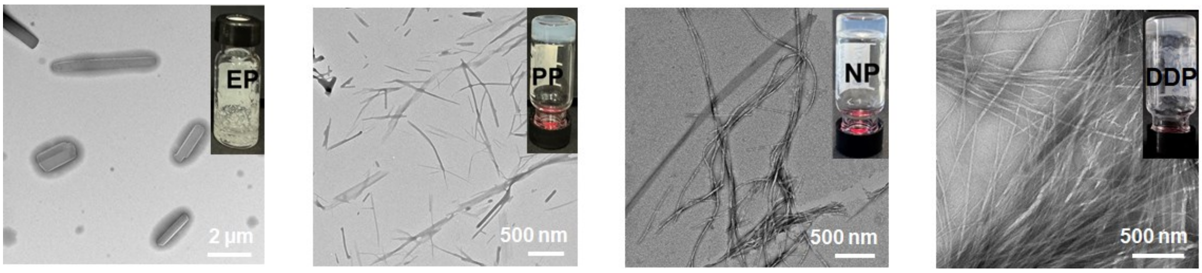

Figure S15. TEM images showing the assemblies obtained from synthesized Boc-FL-NH_2 when mixed with the corresponding phosphate salts (**EP**, **PP**, **NP** and **DDP**) in 0.6 M borate buffer, pH 9.1.

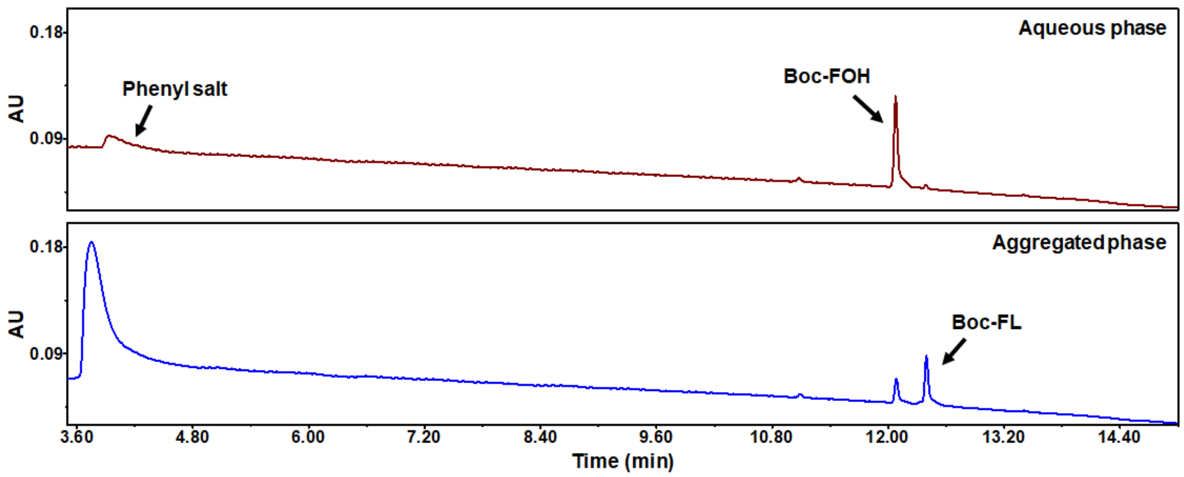

Figure S16. UPLC chromatograms of reactions between 10 mM L-NH_2 with 10 mM **Boc-FPP** in aqueous (top) and aggregated phase (bottom), in 0.6 M borate buffer, pH 9.1. The aggregated phase was dissolved in THF:H_2O (1:1) to analyse in UPLC.

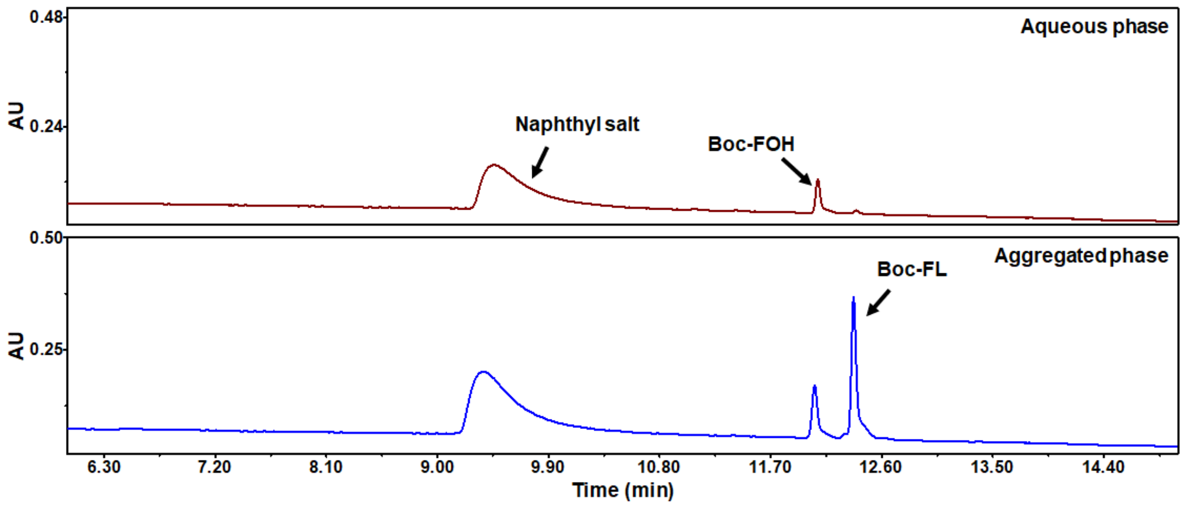

Figure S17. UPLC chromatograms of reactions between 10 mM L-NH_2 with 10 mM **Boc-FNP** in aqueous (top) and aggregated phase (bottom), in 0.6 M borate buffer, pH 9.1. The aggregated phase was dissolved in THF:H_2O (1:1) to analyse in UPLC.

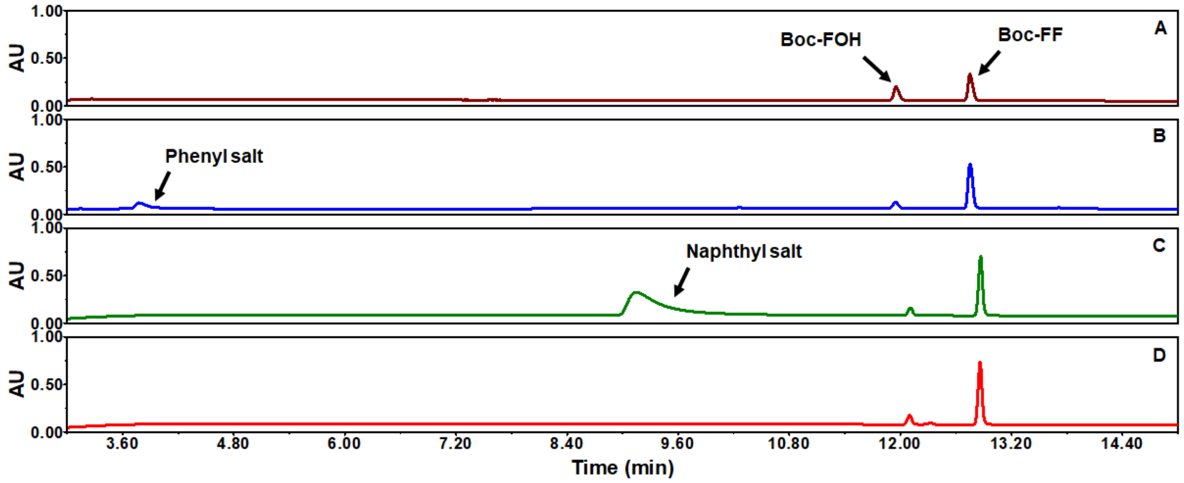

Figure S18. UPLC chromatograms of reactions between 10 mM F-NH_2 with **A)** 10 mM **Boc-FEP**, **B)** 10 mM **Boc-FPP**, **C)** 10 mM **Boc-FNP** and **D)** 10 mM **Boc-FDDP**, in 0.6 M borate buffer, pH 9.1. Measurements were taken after 1 hour.

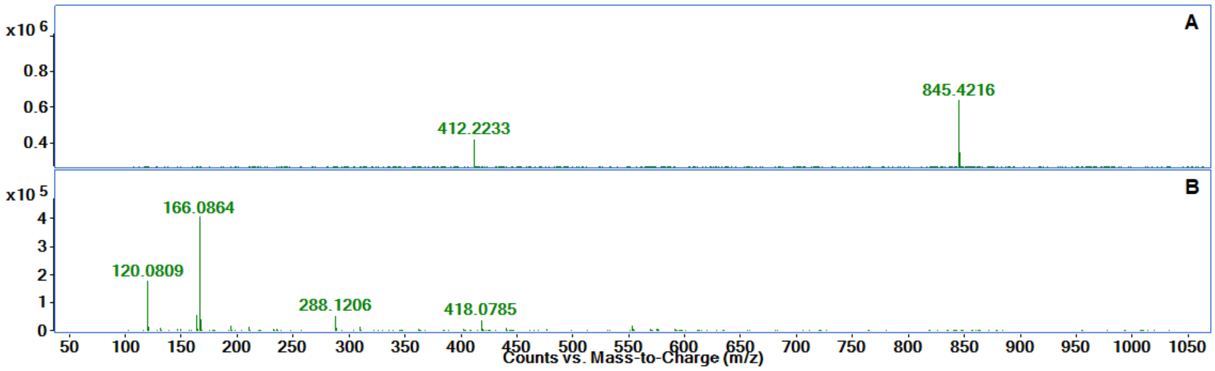

Figure S19. Mass spectra of peptide formed between10 mM F-NH_2 with 10 mM Boc-protected amino acyl phosphate ester (**Boc-FEP**, **Boc-FPP**, **Boc-FNP** and **Boc-FDDP**) shown for: **A)** Boc-FF-NH_2 (retention time 12.86 min), **B)** Boc-F-OH (retention time 12.11 min), in Supporting Figure S18, obtained from the LC-MS analysis.

Calculated m/z $[M+H]^+$: **Boc-FF-NH_2** 412.2231, **Boc-F-OH** 266.1387.

Observed m/z $[M+H]^+$: **Boc-FF-NH_2** 412.2233, $[M+Na]^+$: **Boc-F-OH** 288.1206.

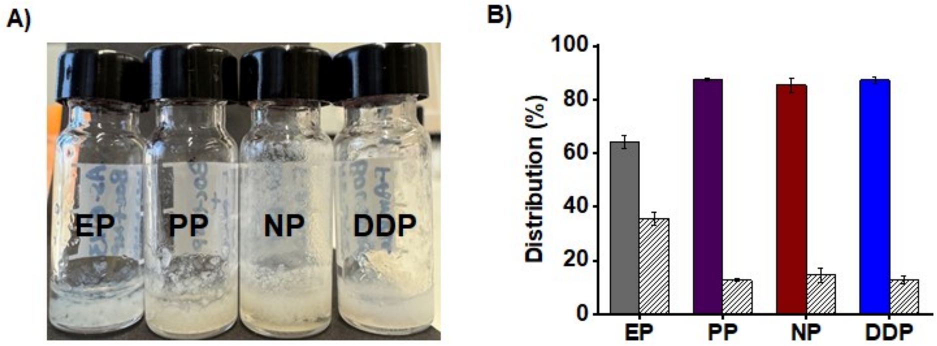

Figure S20. A) Digital images of reaction vials from peptide coupling between 10 mM F-NH_2 and Boc-protected aminoacyl phosphates (**EP**, **PP**, **NP**, and **DDP**) at equimolar concentrations. **B**) Corresponding peptide products under the same conditions. Solid bars correspond to peptide product (Boc-FF-NH_2) while striped bars indicate hydrolysis (Boc-F-OH). All reactions were carried out in 0.6 M borate buffer at pH 9.1. Peptide bond formation was measured at 1h. Error bars represent standard deviation from three independent experiments.

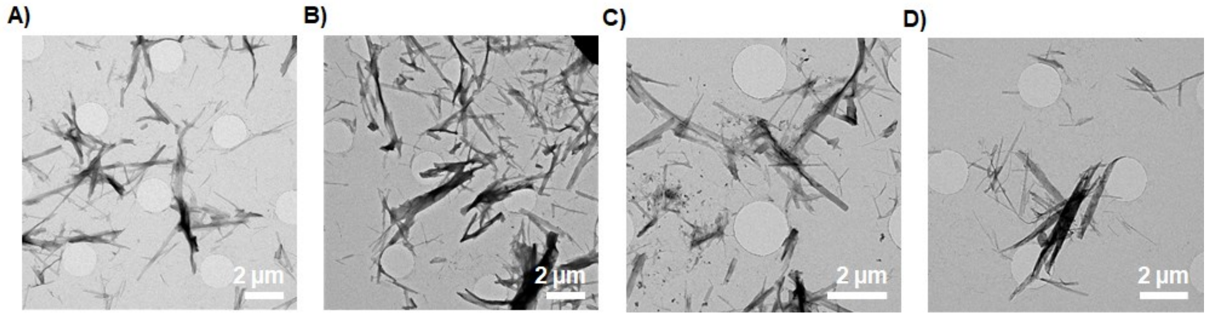

Figure S21. TEM images showing the assemblies obtained from reactions of Boc-protected aminoacyl phosphates with F-NH_2. Images were taken after 1 h.

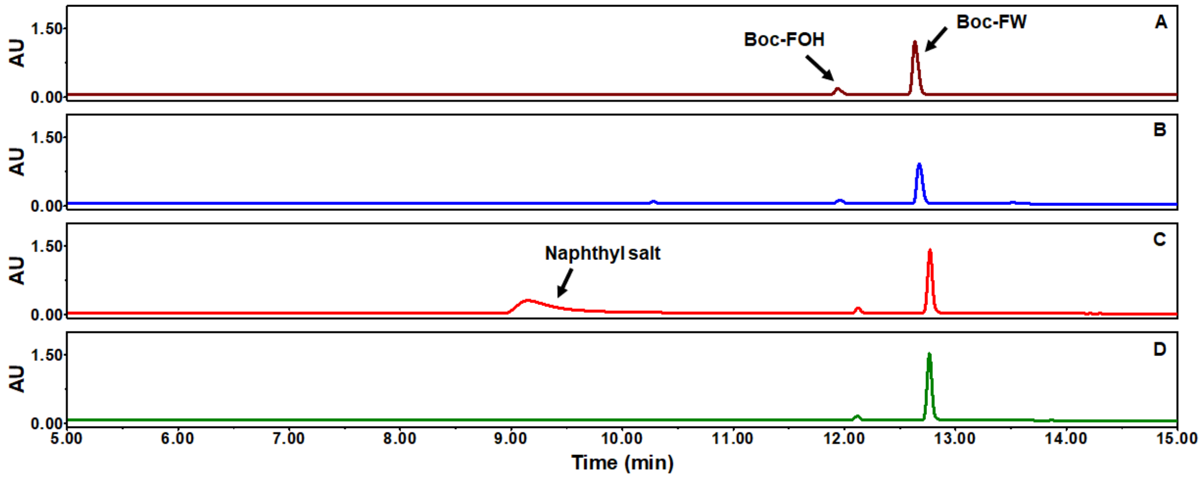

Figure S22. UPLC chromatograms of reactions between 10 mM W-NH_2 with **A)** 10 mM **Boc-FEP**, **B)** 10 mM **Boc-FPP**, **C)** 10 mM **Boc-FNP** and **D)** 10 mM **Boc-FDDP**, in 0.6 M borate buffer, pH 9.1. Measurements were taken after 1 hour.

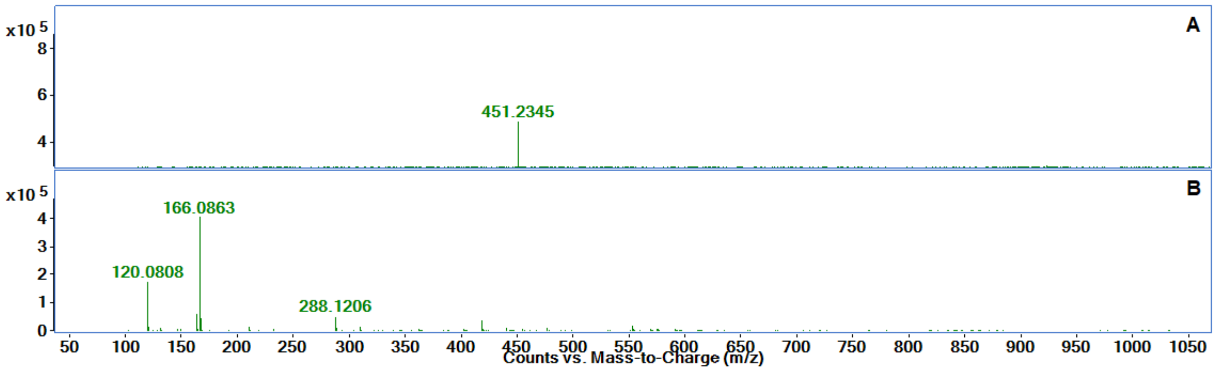

Figure S23. Mass spectra of peptide formed between10 mM W-NH_2 and 10 mM Boc-protected amino acyl phosphate ester (**Boc-FEP**, **Boc-FPP**, **Boc-FNP** and **Boc-FDDP**) shown for: **A)** Boc-FW-NH_2 (retention time 12.76 min), **B)** Boc-F-OH (retention time 12.11 min), in Supporting Figure S22, obtained from the LC-MS analysis.

Calculated m/z $[M+H]^+$: **Boc-FW-NH_2** 451.2340, **Boc-FOH** 266.1387.

Observed m/z $[M+H]^+$: **Boc-FW-NH_2** 451.2345, $[M+Na]^+$: **Boc-FOH** 288.1206.

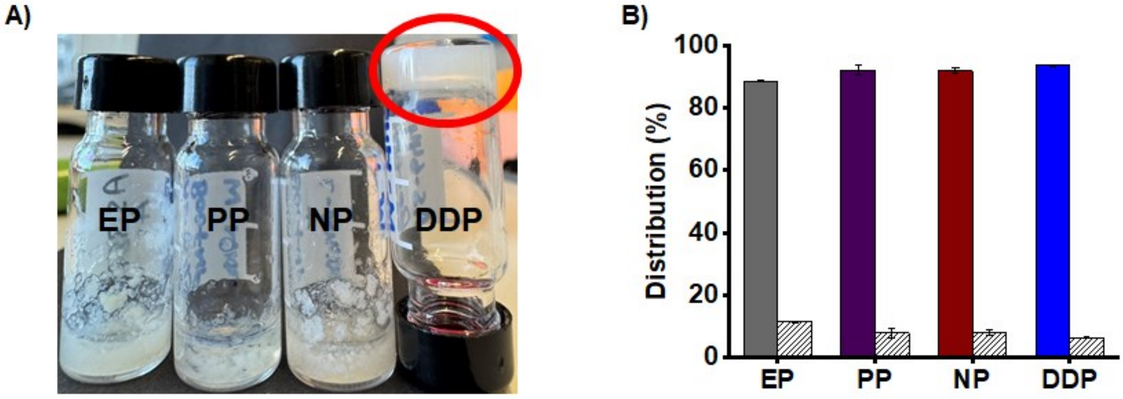

Figure S24. A) Digital images of reaction vials from peptide coupling between 10 mM W-NH_2 and Boc-protected aminoacyl phosphates (**EP**, **PP**, **NP**, and **DDP**) at equimolar concentrations. **B**) Corresponding peptide products under the same conditions. Solid bars correspond to peptide product (Boc-FW-NH_2) while striped bars indicate hydrolysis (Boc-F-OH). All reactions were carried out in 0.6 M borate buffer at pH 9.1. Peptide bond formation was measured at 1h. Error bars represent standard deviation from three independent experiments.

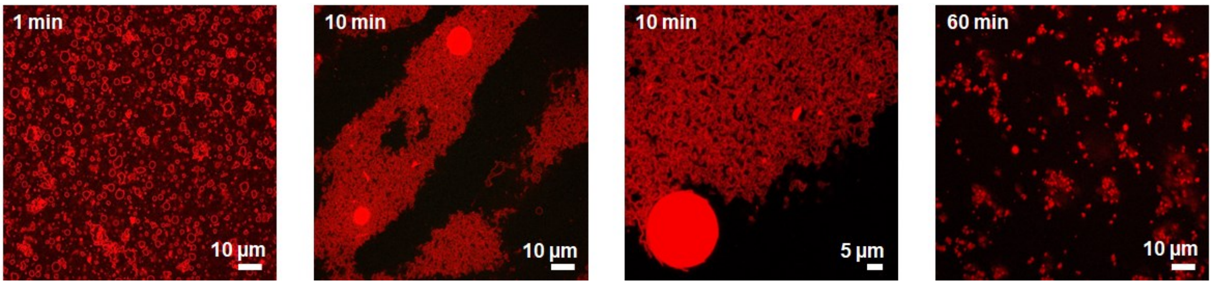

Figure S25. Time-dependent confocal images (nile red staining) of reaction between 10 mM W-NH_2 with 10 mM **Boc-FDDP** at different time points. Reaction was carried out in 0.6 M borate buffer at pH 9.1.

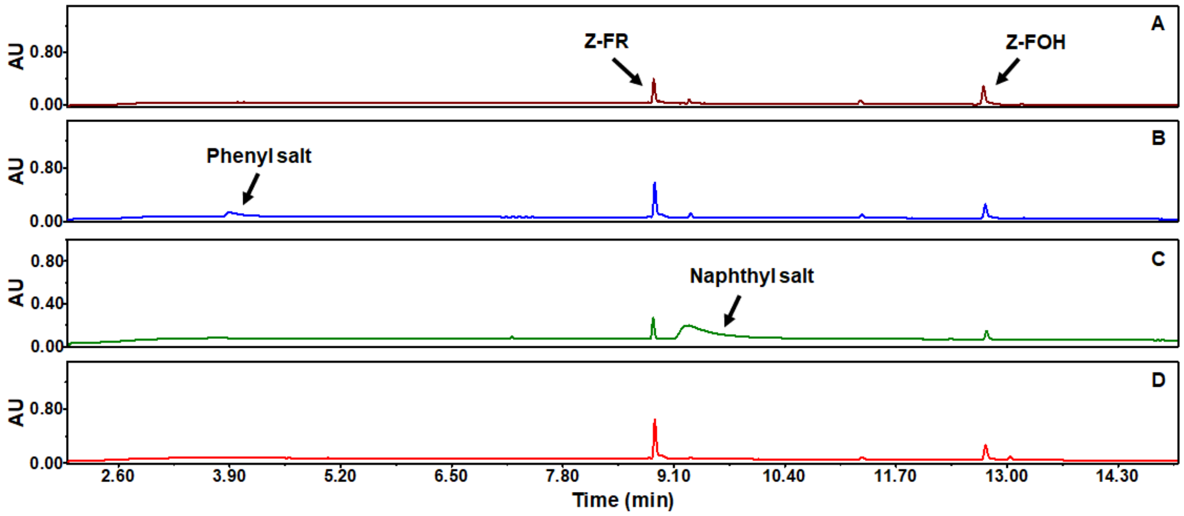

Figure S26. UPLC chromatograms of reactions between 10 mM R-NH_2 with **A)** 10 mM **Z-FEP**, **B)** 10 mM **Z-FPP**, **C)** 10 mM **Z-FNP** and **D)** 10 mM **Z-FDDP**, in 0.6 M borate buffer, pH 9.1. Measurements were taken after 1 hour.

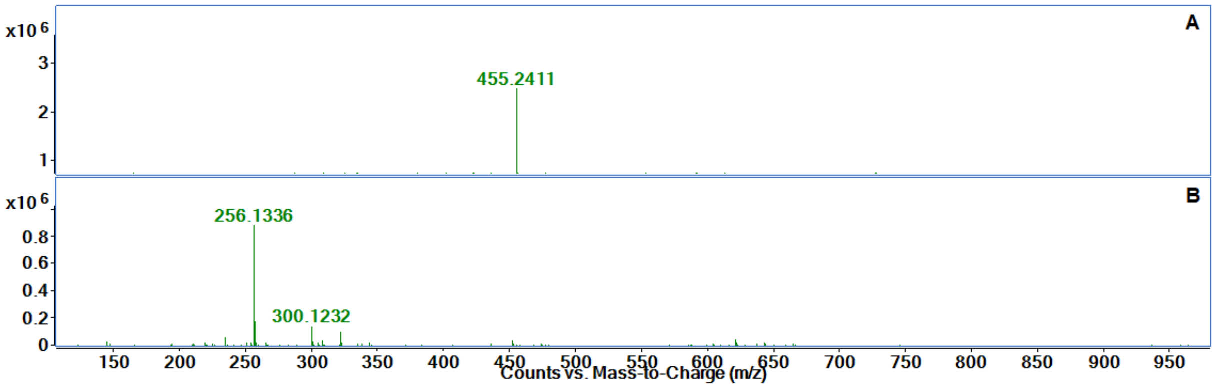

Figure S27. Mass spectra of peptide formed between 10 mM R-NH_2 and 10 mM Z-protected amino acyl phosphate ester (**Z-FEP**, **Z-FPP**, **Z-FNP,** and **Z-FDDP**) shown for: **A)** Z-FR-NH_2 (retention time 8.91 min), **B)** Z-F-OH (retention time 12.76 min), in Supporting Figure S26, obtained from the LC-MS analysis.

Calculated m/z $[M+H]^+$: **Z-FR-NH₂** 455.2401, **Z-F-OH** 300.1230.

Observed m/z $[M+H]^+$: **Z-FR-NH₂** 455.2411, **Z-F-OH** 300.1232.

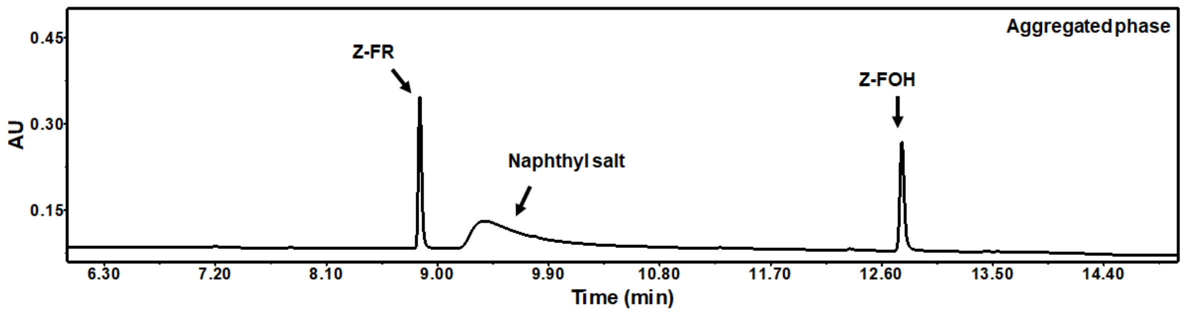

Figure S28. UPLC chromatogram of aggregated phase of reaction between 10 mM R-NH_2 with 10 mM **Z-FNP**, in 0.6 M borate buffer, pH 9.1.

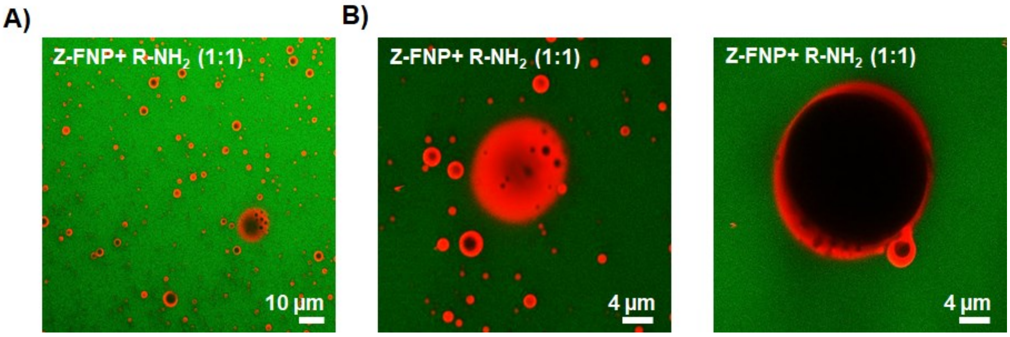

Figure S29. Confocal microscopy images of reaction between 10 mM R-NH_2 with 10 mM **Z-FNP**, incubated with AF488 (green) and Nile red (red) in 0.6 M borate buffer, pH 9.1.

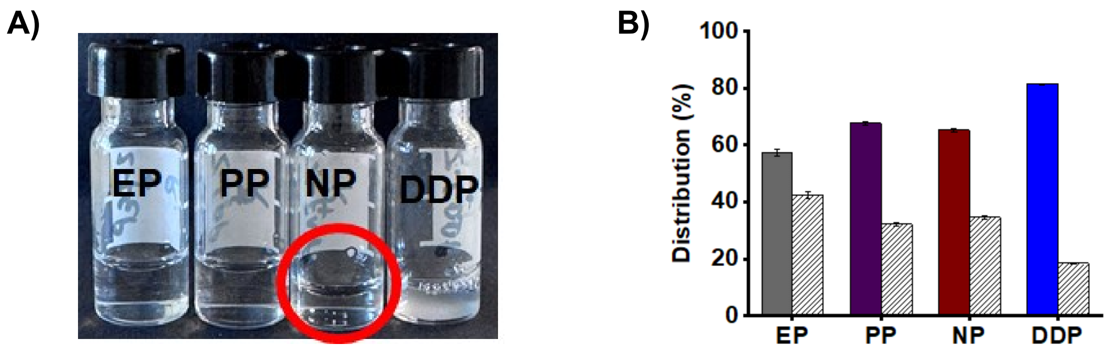

Figure S30. A) Digital images of reaction vials from peptide coupling between 10 mM R-NH_2 and Z-protected aminoacyl phosphates (**EP**, **PP**, **NP**, and **DDP**) at equimolar concentrations. **B**) Corresponding peptide products under the same conditions. Solid bars correspond to peptide product (Z-FR-NH_2) while striped bars indicate hydrolysis (Z-F-OH). All reactions were carried out in 0.6 M borate buffer at pH 9.1. Peptide bond formation was measured at 1h. Error bars represent standard deviation from three independent experiments.

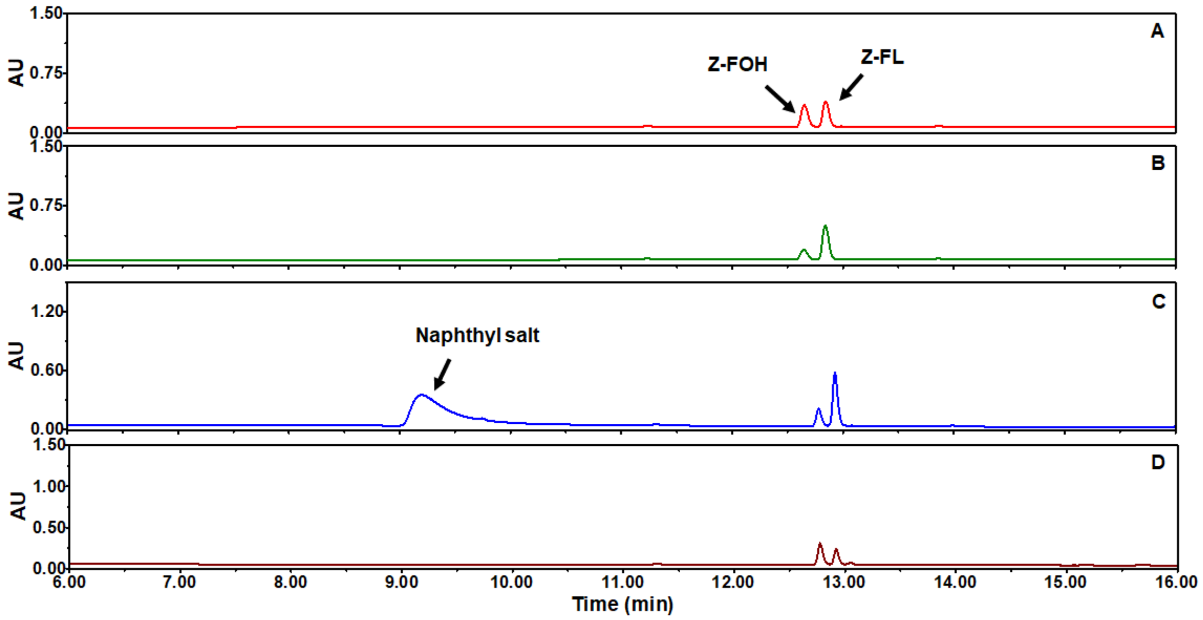

Figure S31. UPLC chromatograms of reactions between 10 mM L-NH_2 with **A)** 10 mM **Z-FEP**, **B)** 10 mM **Z-FPP**, **C)** 10 mM **Z-FNP** and **D)** 10 mM **Z-FDDP**, in 0.6 M borate buffer, pH 9.1. Measurements were taken after 1 hour.

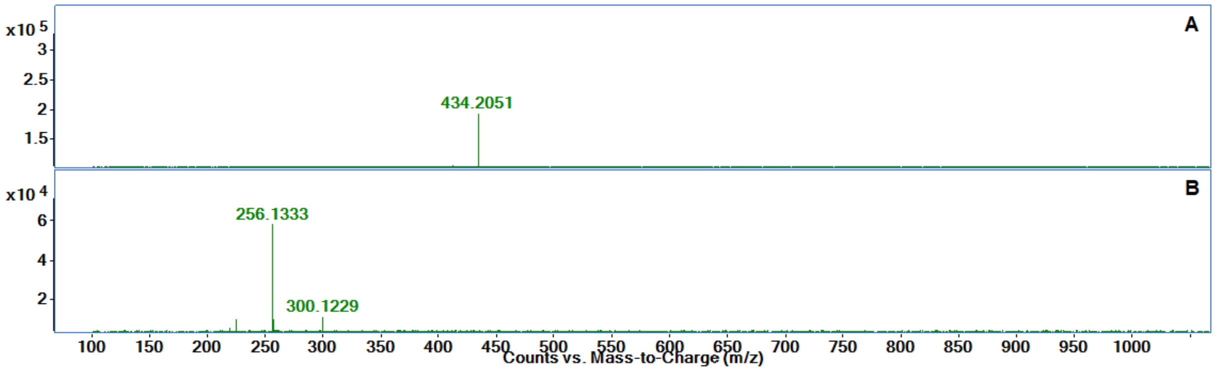

Figure S32. Mass spectra of peptide formed between 10 mM L-NH_2 and 10 mM Z-protected amino acyl phosphate ester (**Z-FEP**, **Z-FPP**, **Z-FNP,** and **Z-FDDP**) shown for: **A)** Z-FL-NH_2 (retention time 12.91 min), **B)** Z-F-OH (retention time 12.75 min), in Supporting Figure S31, obtained from the LC-MS analysis.

Calculated m/z $[M+H]^+$: **Z-FL-NH_2** 434.2050, **Z-F-OH** 300.1230.

Observed m/z $[M+H]^+$: **Z-FL-NH_2** 434.2051, **Z-F-OH** 300.1229.

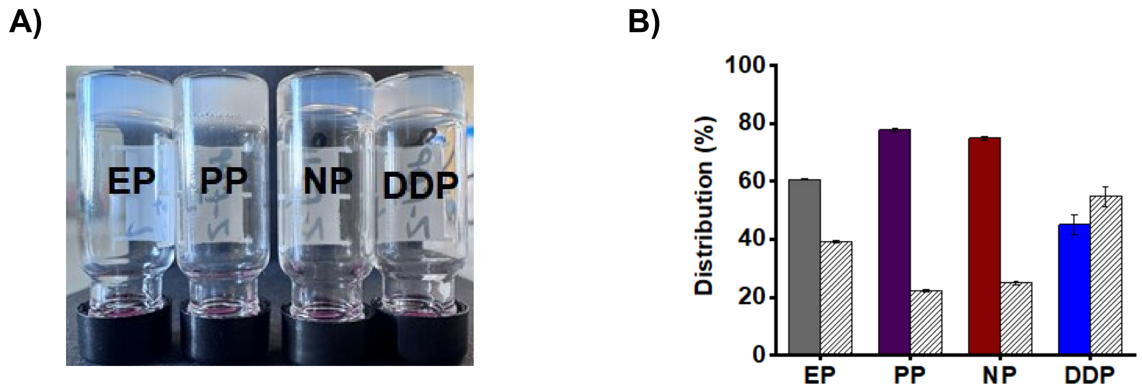

Figure S33. A) Digital images of reaction vials from peptide coupling between 10 mM L-NH_2 and Z-protected aminoacyl phosphates (**EP**, **PP**, **NP**, and **DDP**) at equimolar concentrations. **B**) Corresponding peptide products under the same conditions. Solid bars correspond to peptide product (Z-FL-NH_2) while striped bars indicate hydrolysis (Z-F-OH). All reactions were carried out in 0.6 M borate buffer at pH 9.1. Peptide bond formation was measured at 1h. Error bars represent standard deviation from three independent experiments.

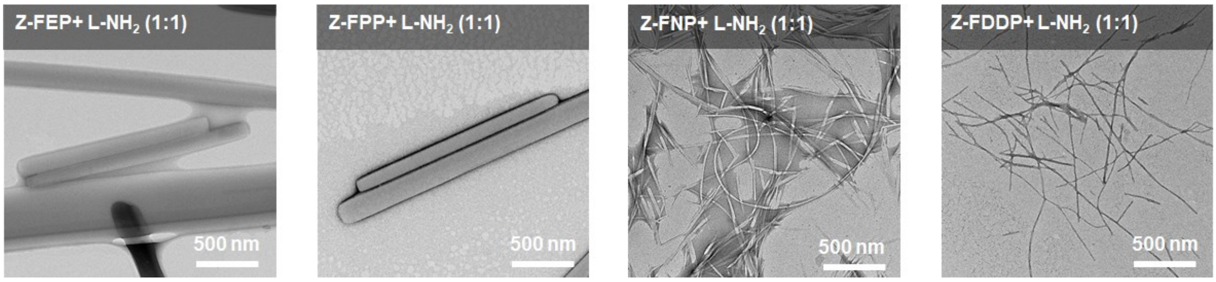

Figure S34. TEM images showing the assemblies obtained from reactions of Z-protected aminoacyl phosphates with L-NH_2. Images were taken after 1 h.

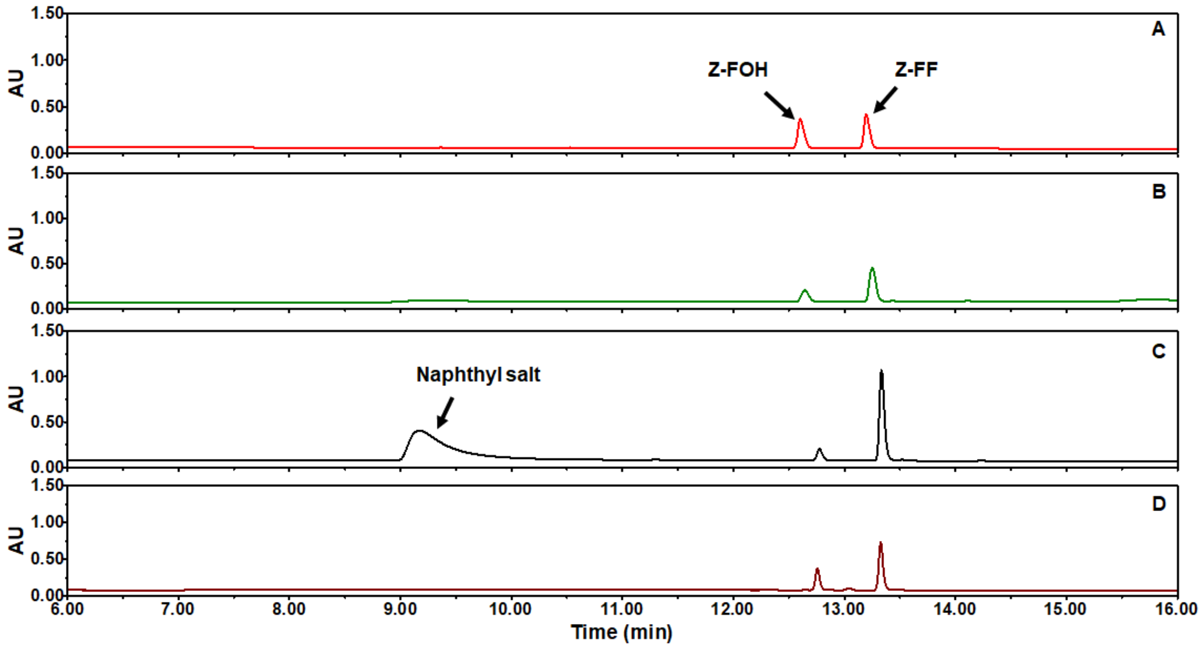

Figure S35. UPLC chromatograms of reactions between 10 mM F-NH_2 with **A)** 10 mM **Z-FEP**, **B)** 10 mM **Z-FPP**, **C)** 10 mM **Z-FNP** and **D)** 10 mM **Z-FDDP**, in 0.6 M borate buffer, pH 9.1. Measurements were taken after 1 hour.

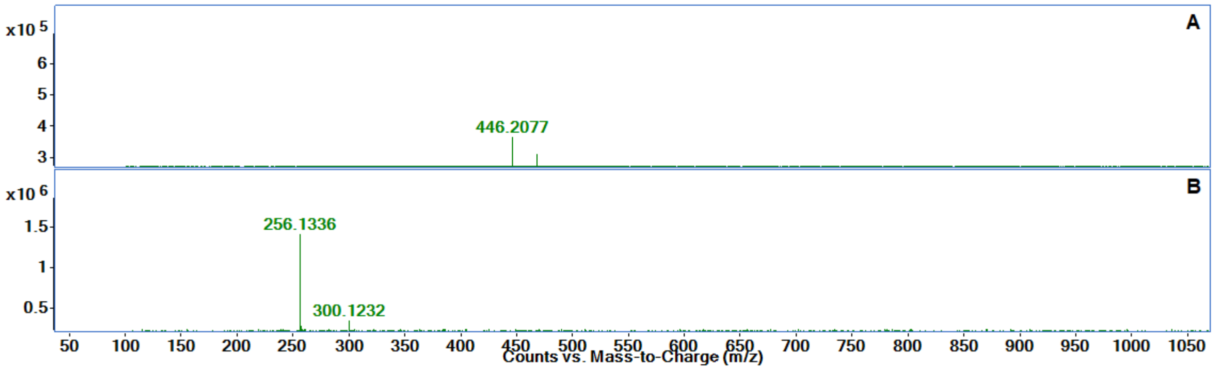

Figure S36. Mass spectra of peptide formed between 10 mM F-NH_2 and 10 mM Z-protected amino acyl phosphate ester (**Z-FEP**, **Z-FPP**, **Z-FNP,** and **Z-FDDP**) shown for: **A)** Z-FF-NH_2 (retention time 13.32 min), **B)** Z-F-OH (retention time 12.75 min), in Supporting Figure S35, obtained from the LC-MS analysis.

Calculated m/z $[M+H]^+$: **Z-FF-NH_2** 446.2074, **Z-F-OH** 300.1230.

Observed m/z $[M+H]^+$: **Z-FF-NH_2** 446.2077, **Z-F-OH** 300.1232.

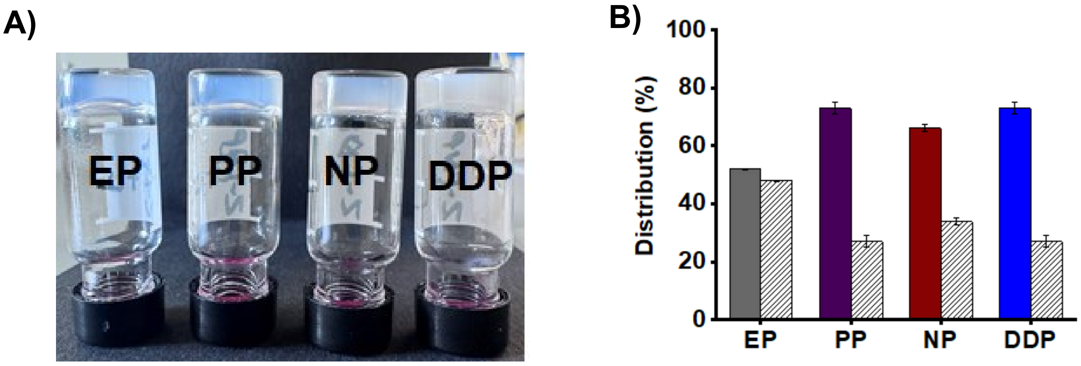

Figure S37. A) Digital images of reaction vials from peptide coupling between 10 mM F-NH_2 and Z-protected aminoacyl phosphates (**EP**, **PP**, **NP**, and **DDP**) at equimolar concentrations. **B**) Corresponding peptide products under the same conditions. Solid bars correspond to peptide product (Z-FF-NH_2) while striped bars indicate hydrolysis (Z-F-OH). All the reactions were carried out in 0.6 M borate buffer at pH 9.1. Peptide bond formation was measured at 1h. Error bars represent standard deviation from three independent experiments.

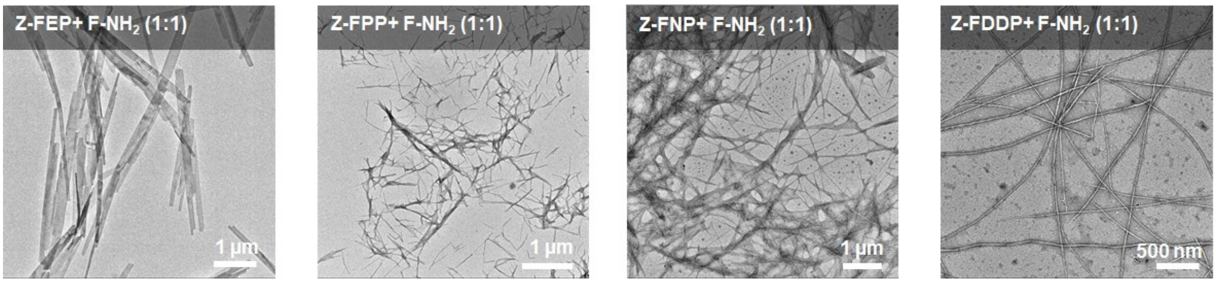

Figure S38. TEM images showing the assemblies obtained from reactions of Z-protected aminoacyl phosphates with F-NH_2. Images were taken after 1 h.

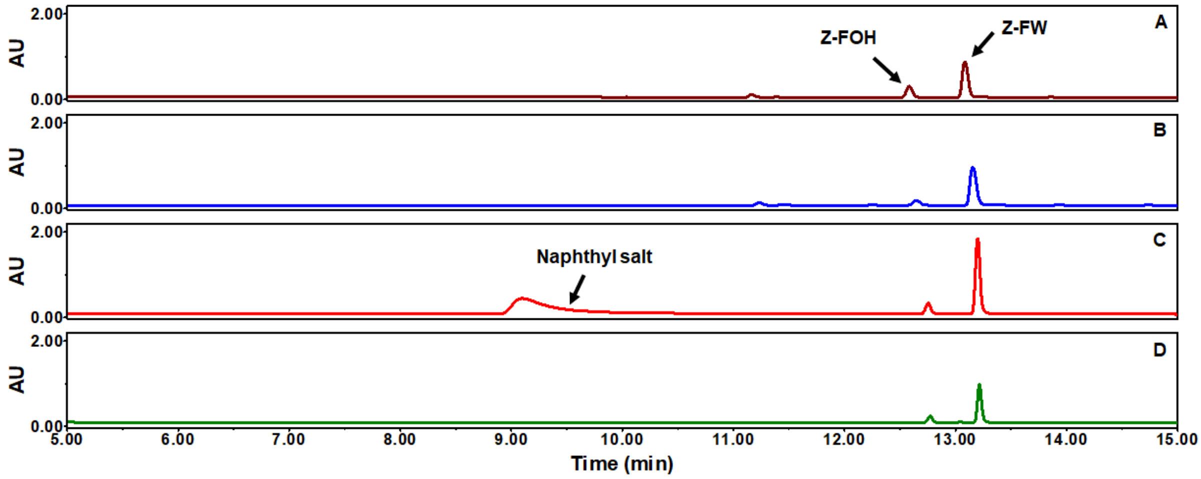

Figure S39. UPLC chromatograms of reactions between 10 mM W-NH_2 with **A)** 10 mM **Z-FEP**, **B)** 10 mM **Z-FPP**, **C)** 10 mM **Z-FNP** and **D)** 10 mM **Z-FDDP**, in 0.6 M borate buffer, pH 9.1. Measurements were taken after 1 hour.

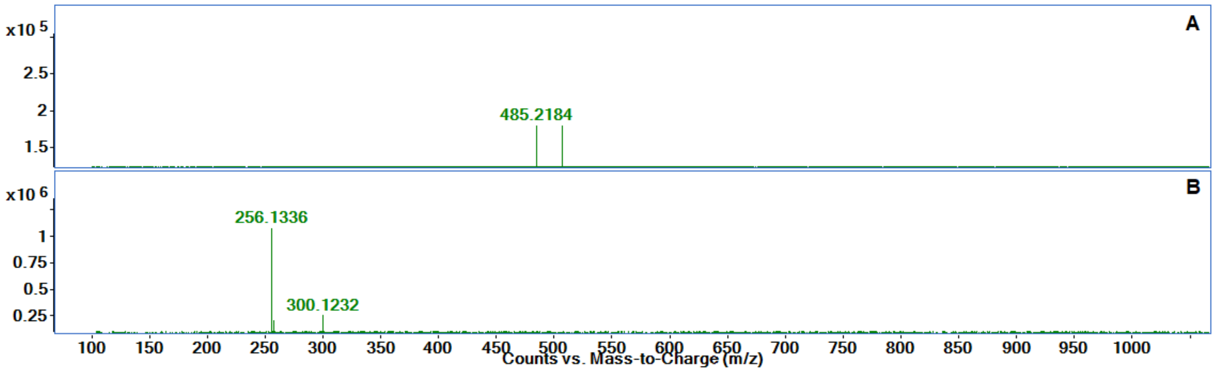

Figure S40. Mass spectra of peptide formed between 10 mM W-NH_2 and 10 mM Z-protected amino acyl phosphate ester (**Z-FEP**, **Z-FPP**, **Z-FNP,** and **Z-FDDP**) shown for: **A)** Z-FW-NH_2 (retention time 13.19 min), **B)** Z-F-OH (retention time 12.75 min), in Supporting Figure S39, obtained from the LC-MS analysis.

Calculated m/z $[M+H]^+$: **Z-FW-NH_2** 485.2183, **Z-F-OH** 300.1230.

Observed m/z $[M+H]^+$: **Z-FW-NH_2** 485.2184, **Z-F-OH** 300.1232.

A) B)

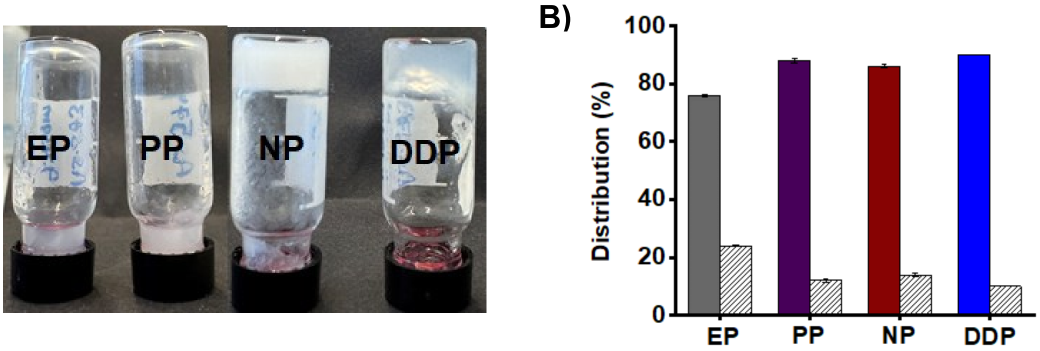

Figure S41. A) Digital images of reaction vials from peptide coupling between 10 mM W-NH_2 and Z-protected aminoacyl phosphates (**EP**, **PP**, **NP**, and **DDP**) at equimolar concentrations. **B)** Corresponding peptide products under the same conditions. Solid bars correspond to peptide product (Z-FW-NH_2) while striped bars indicate hydrolysis (Z-F-OH). All the reactions were carried out in 0.6 M borate buffer at pH 9.1. Peptide bond formation was measured at 1h. Error bars represent standard deviation from three independent experiments.

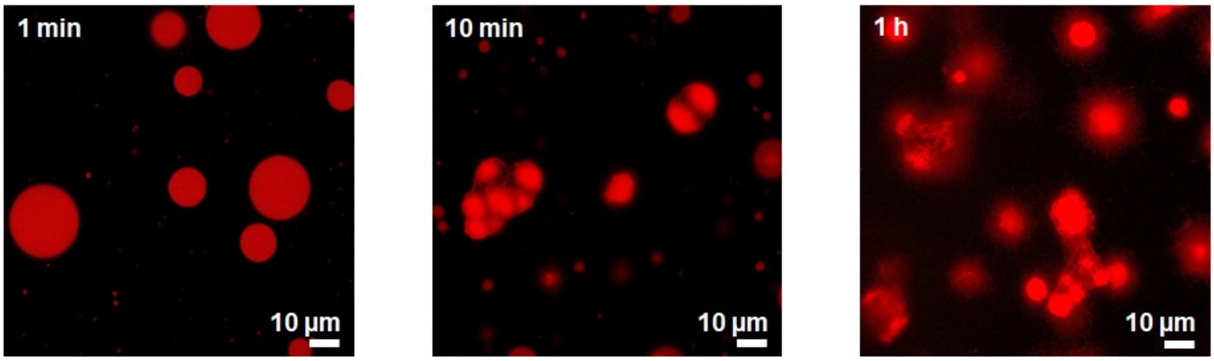

Figure S42. Time-dependent confocal images (nile red staining) of reaction between 10 mM W-NH_2 and 10 mM **Z-FDDP** at different time points. Reaction was carried out in 0.6 M borate buffer at pH 9.1.

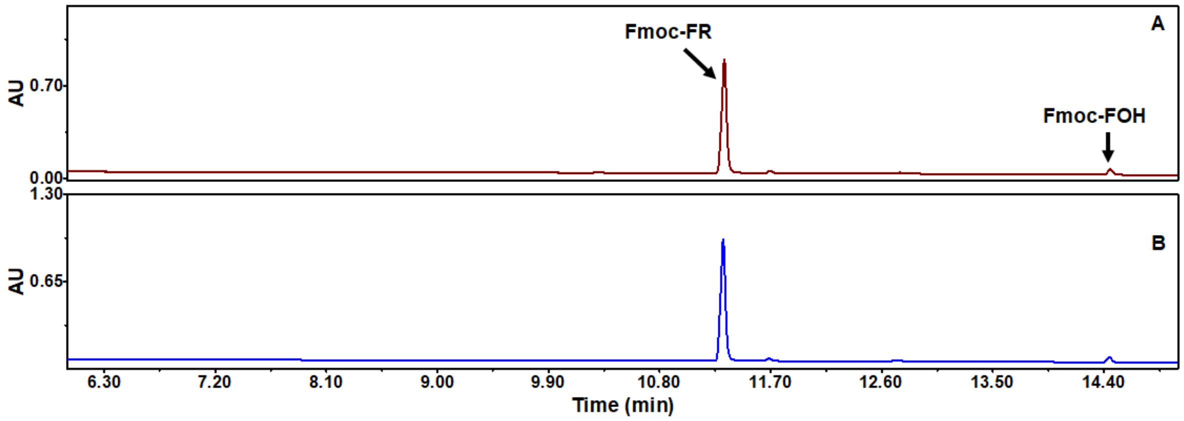

Figure S43. UPLC chromatograms of reactions between 10 mM R-NH_2 and **A)** 10 mM **Fmoc-FEP**, **B)** 10 mM **Fmoc -FPP**, in 0.6 M borate buffer, pH 9.1. Measurements were taken after 1 hour.

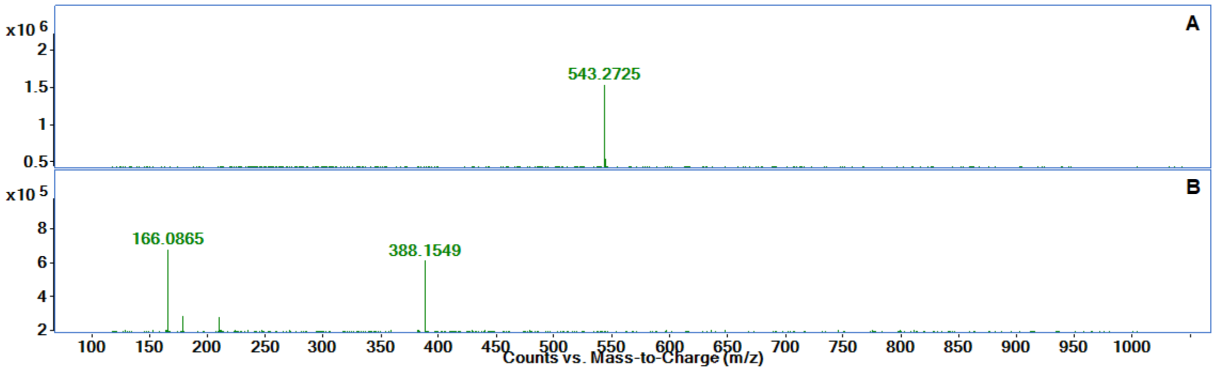

Figure S44. Mass spectra of peptide formed between10 mM R-NH_2 and 10 mM Fmoc-protected amino acyl phosphate ester (**Fmoc-FEP** and **Fmoc-FPP**) shown for: **A)** Fmoc-FR-NH_2 (retention time 11.31 min), **B)** Fmoc-F-OH (retention time 14.45 min), in Supporting Figure S 43, obtained from the LC-MS analysis.

Calculated m/z $[M+H]^+$: **Fmoc-FR-NH_2** 543.2714, **Fmoc-F-OH** 388.1543.

Observed m/z $[M+H]^+$: **Fmoc-FR-NH_2** 543.2725, **Fmoc-F-OH** 388.1549.

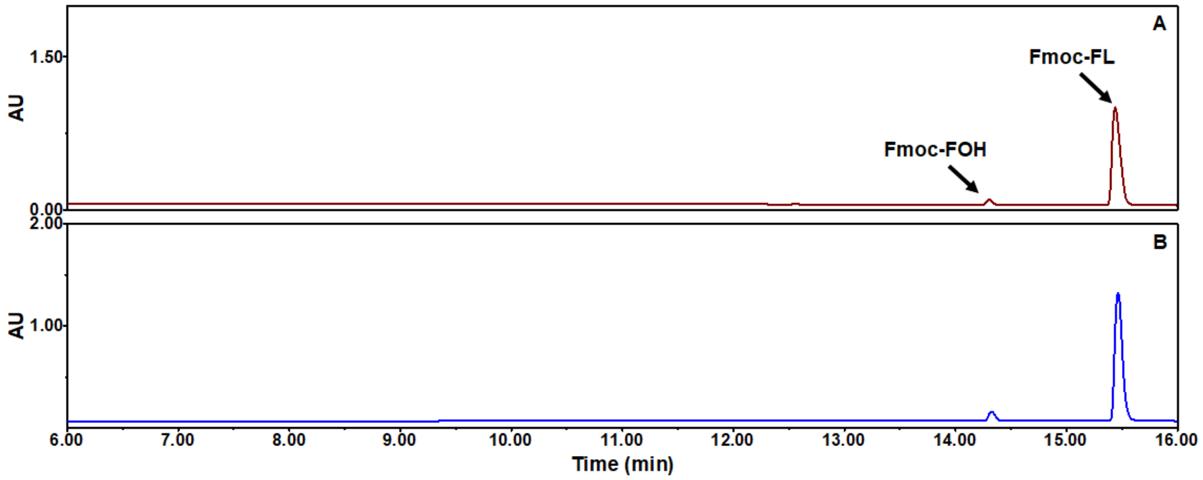

Figure S45. UPLC chromatograms of reactions between 10 mM L-NH_2 with **A)** 10 mM **Fmoc-FEP** and **B)** 10 mM **Fmoc -FPP**, in 0.6 M borate buffer, pH 9.1. Measurements were taken after 1 hour.

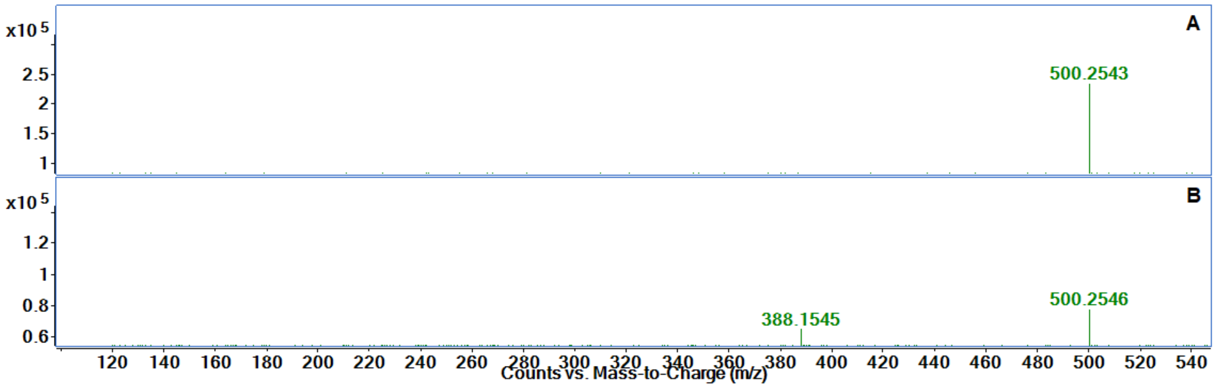

Figure S46. Mass spectra of peptide formed between10 mM L-NH_2 and 10 mM Fmoc-protected amino acyl phosphate ester (**Fmoc-FEP** and **Fmoc-FPP**) shown for: **A)** Fmoc-FR-NH_2 (retention time 15.45 min), **B)** Fmoc-F-OH (retention time 14.30 min), in Supporting Figure S 45, obtained from the LC-MS analysis.

Calculated m/z $[M+H]^+$: **Fmoc-FL-NH_2** 500.2544, **Fmoc-F-OH** 388.1543.

Observed m/z $[M+H]^+$: **Fmoc-FL-NH_2** 500.2543, **Fmoc-F-OH** 388.1545.

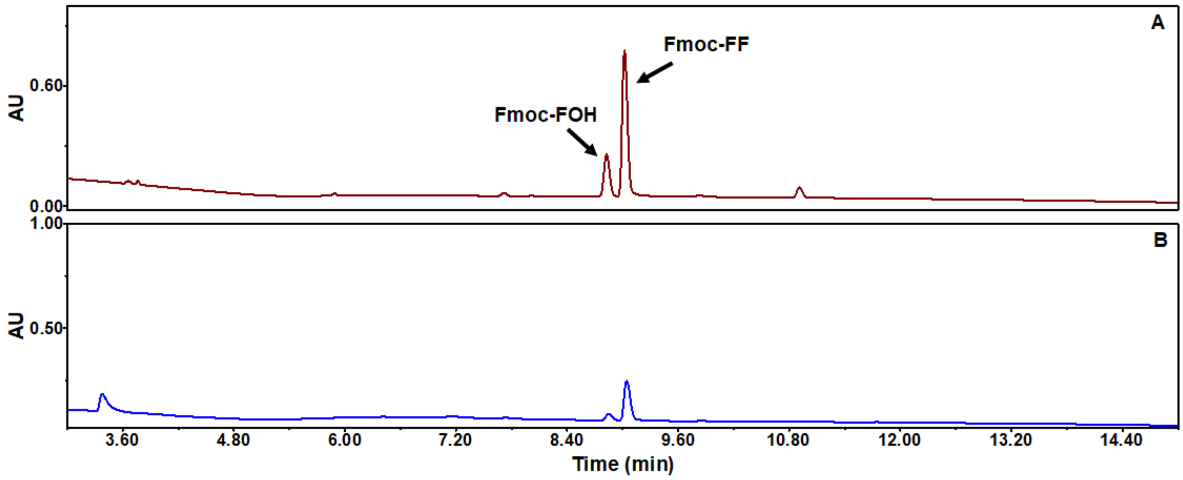

Figure S47. UPLC chromatograms of reactions between 10 mM F-NH_2 with **A)** 10 mM **Fmoc-FEP**, **B)** 10 mM **Fmoc -FPP**, in 0.6 M borate buffer, pH 9.1. Measurements were taken after 1 hour.

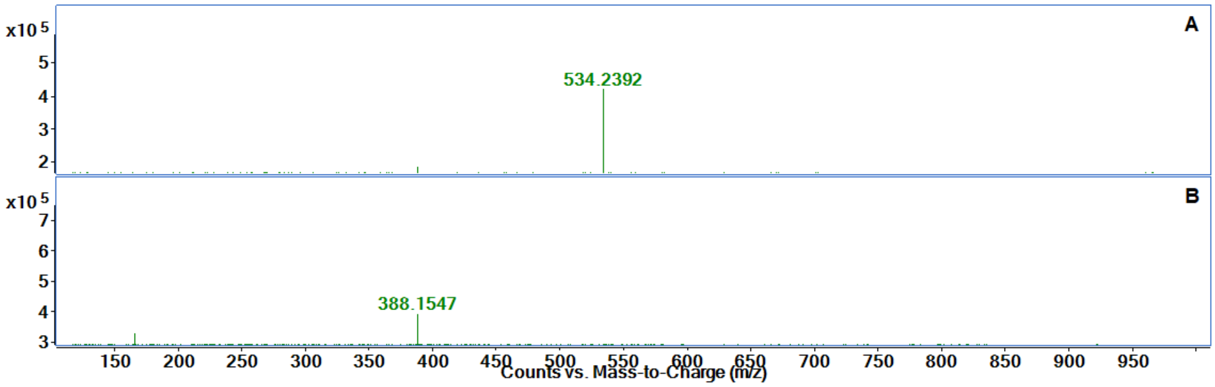

Figure S48. Mass spectra of peptide formed between10 mM F-NH_2 and 10 mM Fmoc-protected amino acyl phosphate ester (**Fmoc-FEP** and **Fmoc-FPP**) shown for: **A)** Fmoc-FF-NH_2 (retention time 9.02 min), **B)** Fmoc-F-OH (retention time 8.82 min), in Supporting Figure S 47, obtained from the LC-MS analysis.

Calculated m/z $[M+H]^+$: **Fmoc-FF-NH_2** 534.2387, **Fmoc-F-OH** 388.1543.

Observed m/z $[M+H]^+$: **Fmoc-FF-NH_2** 534.2392, **Fmoc-F-OH** 388.1547.

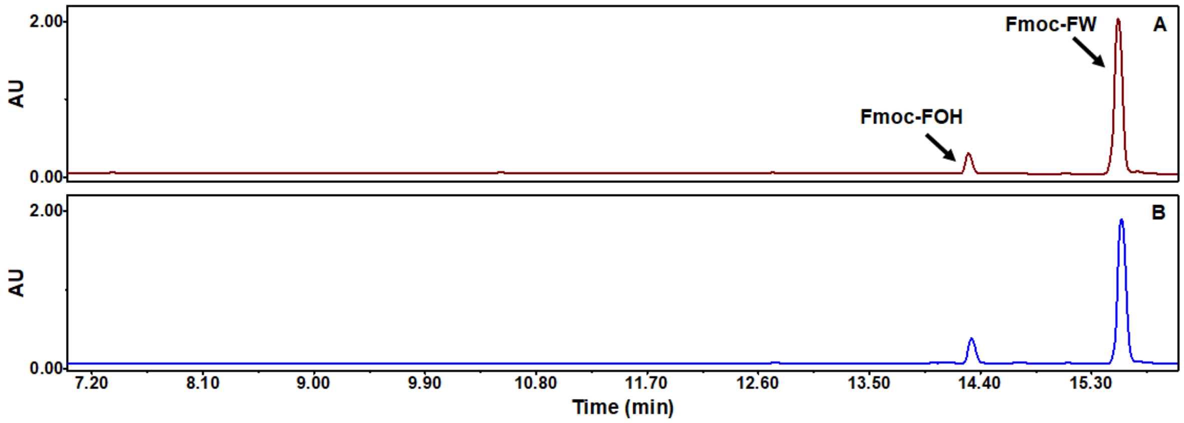

Figure S49. UPLC chromatograms of reactions between 10 mM W-NH_2 with **A)** 10 mM **Fmoc-FEP** and **B)** 10 mM **Fmoc -FPP**, in 0.6 M borate buffer, pH 9.1. Measurements were taken after 1 hour.

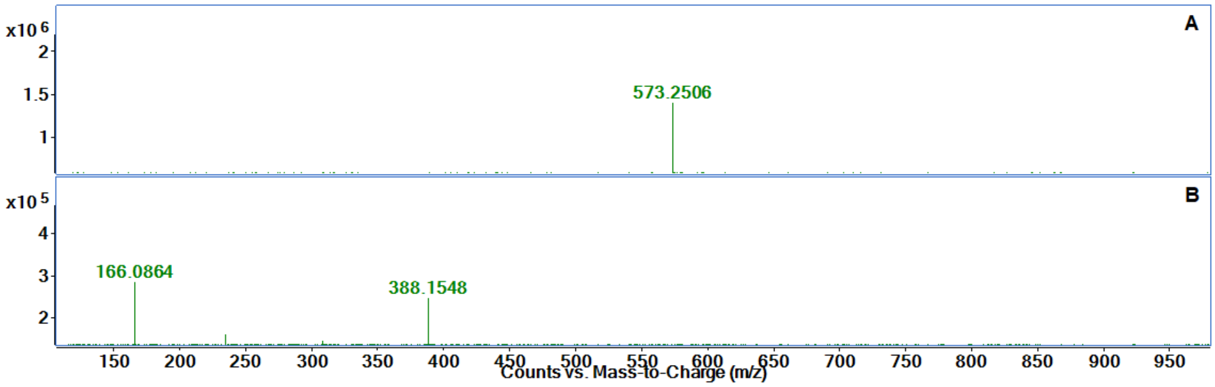

Figure S50. Mass spectra of peptide formed between10 mM W-NH_2 and 10 mM Fmoc-protected amino acyl phosphate ester (**Fmoc-FEP** and **Fmoc-FPP**) shown for: **A)** Fmoc-FW-NH_2 (retention time 15.54 min), **B)** Fmoc-FOH (retention time 14.32 min), in Supporting Figure S 49, obtained from the LC-MS analysis.

Calculated m/z $[M+H]^+$: **Fmoc-FW-NH_2** 573.2496, **Fmoc-F-OH** 388.1543.

Observed m/z $[M+H]^+$: **Fmoc-FW-NH_2** 573.2506, **Fmoc-F-OH** 388.1548.

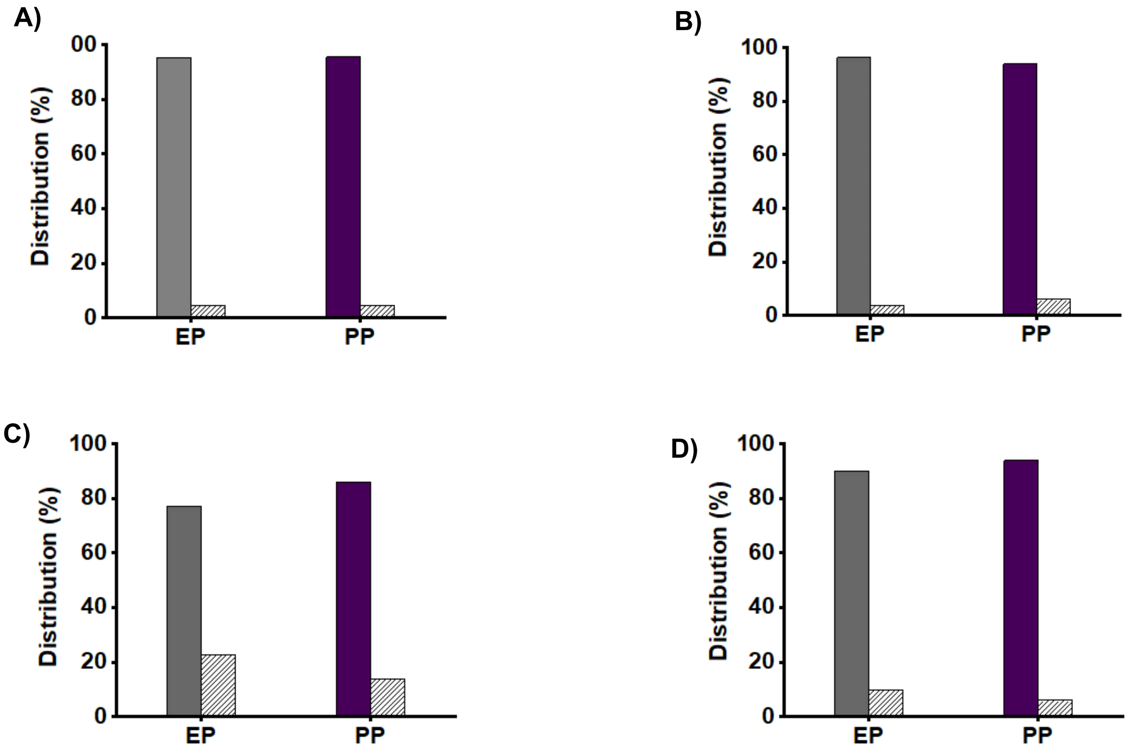

Figure S51. Bar graphs showing peptide coupling from **Fmoc-FEP** and **Fmoc-FPP** with amides in equimolar concentration**s A)** R-NH_2, **B)** L-NH_2, **C)** F-FNH_2 and **D)** W-NH_2 in 0.6 M borate buffer, pH 9.1. Solid bars correspond to peptide products (Fmoc-FR-NH_2, Fmoc-FL-NH_2, Fmoc-FF-NH_2, Fmoc-FW-NH_2) while striped bars indicate hydrolysis (Fmoc-F-OH). Measurements were taken after 1 hour.

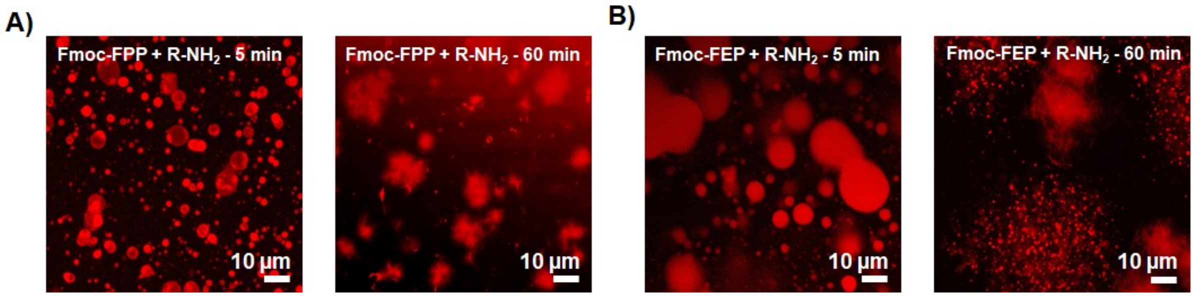

Figure S52. Time dependent confocal microscopy images (nile red staining) of reactions between 10 mM R-NH_2 and **A)** 10 mM **Fmoc-FPP** and **B)** 10 mM **Fmoc-FEP** in 0.6 M borate buffer, pH 9.1.

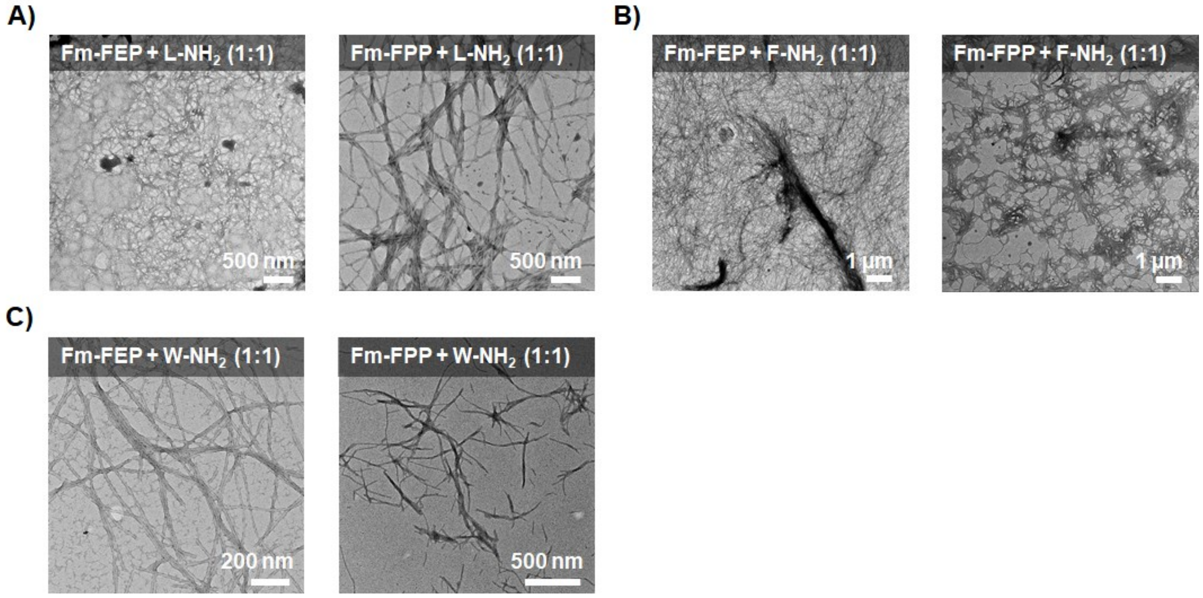

Figure S53. TEM images of reaction between Fmoc- protected amino acyl phosphate esters (**Fmoc-FEP** and **Fmoc-FPP** each at 10 mM concentration) with amide nucleophiles (10 mM): **A)** L-NH_2, **B)** F-NH_2, and **C)** W-NH_2 in 0.6 M borate buffer, pH 9.1. Images were taken after 1 hour.

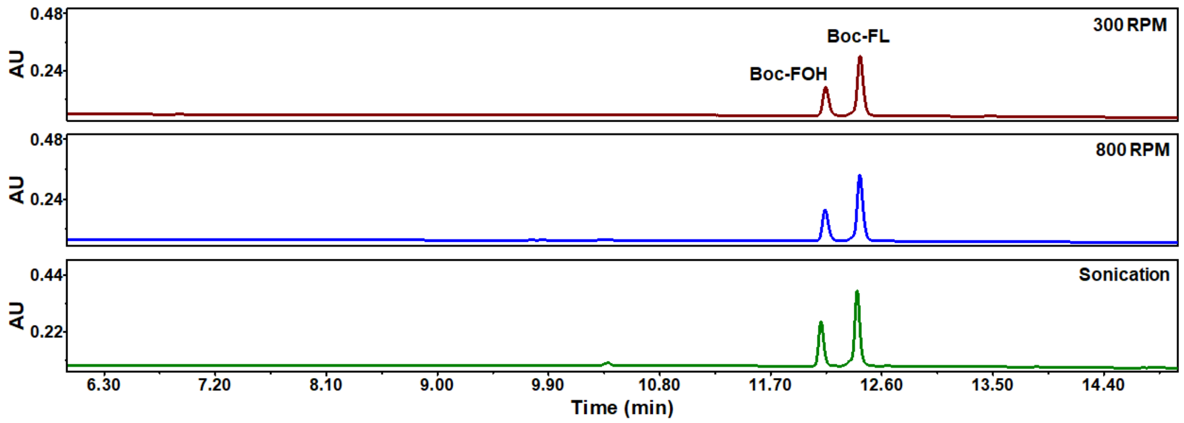

Figure S54. UPLC chromatograms of reactions between 10 mM L-NH_2 with 10 mM **Boc-FDDP** using mechanical force for 1 hour: magnetic stirring at 300 (top), 800 rpm (middle) and sonication (bottom) in 0.6 M borate buffer, pH 9.1. Measurements were taken after 1 hour.

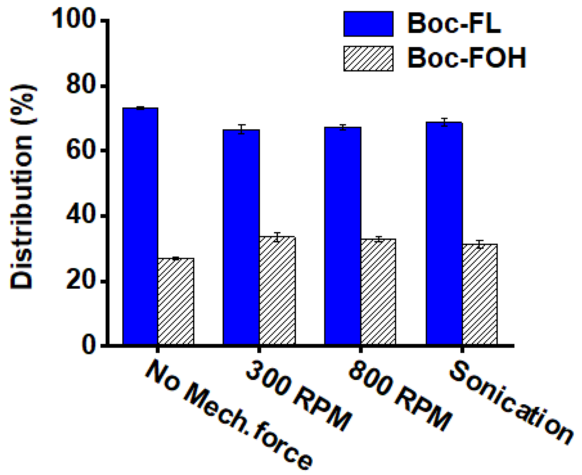

Figure S55. Bar graphs showing peptide product formation of reactions between 10 mM L-NH_2 with 10 mM **Boc-FDDP** at different reaction conditions with and without mechanical force for 1 hour: magnetic stirring at 300 and 800 rpm and sonication. Solid bars correspond to peptide product (Boc-FL-NH_2) while striped bars indicate hydrolysis (Boc-F-OH). All reactions were carried out in 0.6 M borate buffer at pH 9.1. Peptide conversions were measured at 1h. Error bars represent standard deviation from three independent experiments.

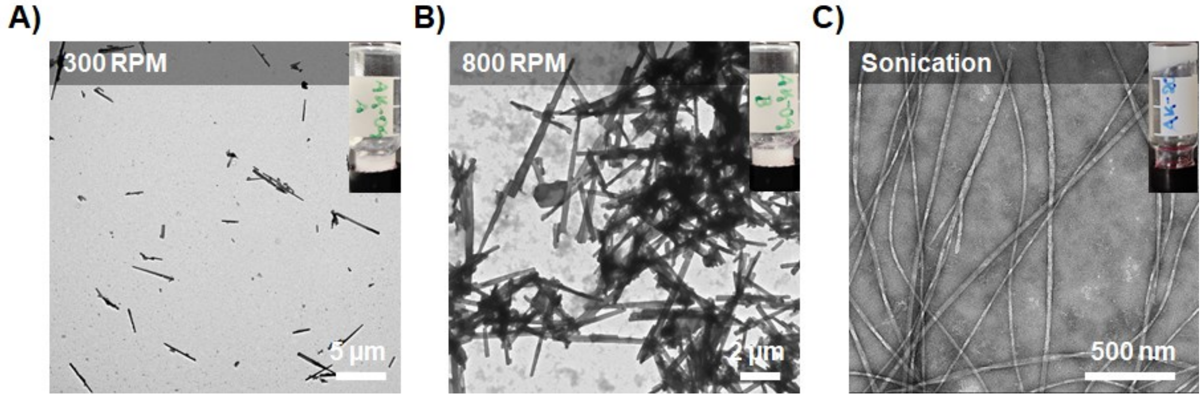

Figure S56. TEM images of reactions between 10 mM L-NH_2 with 10 mM **Boc-FDDP** at different conditions: **A)** magnetic stirring at 300 rpm, **B)** 800 rpm and **C)** Sonication in 0.6 M borate buffer, pH 9.1. Images were taken after 1 hour of initiating the reaction.

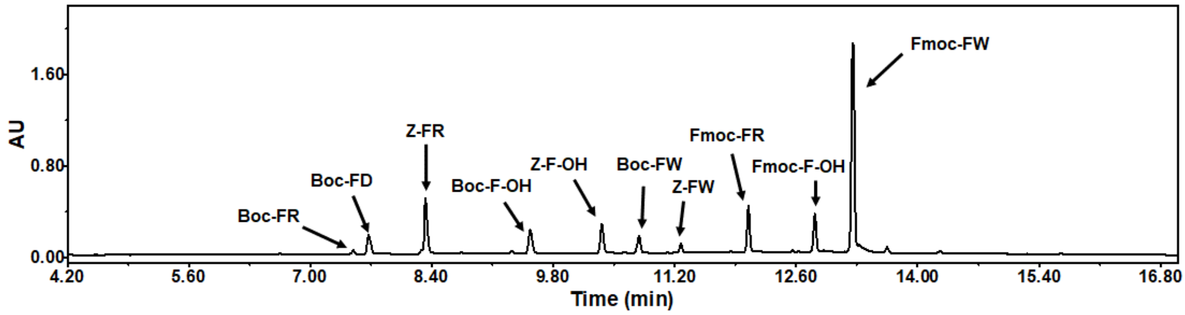

Figure S57. UPLC chromatograms of reactions of amide mixtures (R, W, D; 10 mM each) with amino acyl phosphate esters (**Boc-FEP**, **Z-FDDP**, **Fmoc-FPP**; 10 mM each) in 0.6 M borate buffer at pH 9.1. Measurements were taken after 1 h.

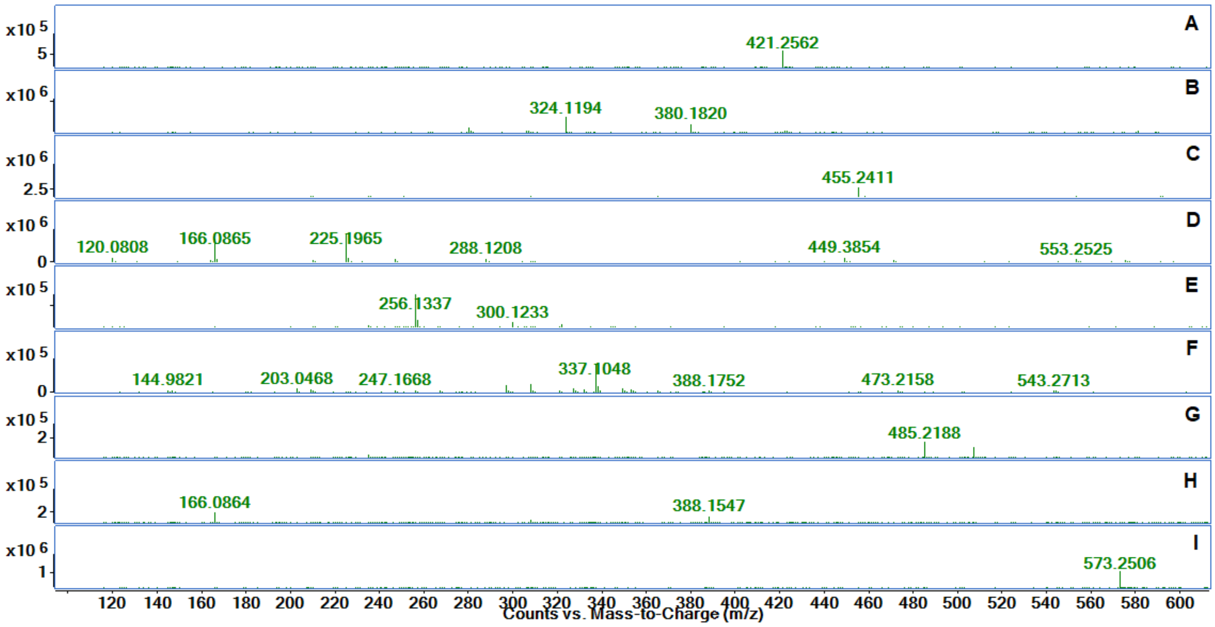

Figure S58. Mass spectra of peptide reactions of amide mixtures (R, W, D; 10 mM each) with amino acyl phosphate esters (**Boc-FEP**, **Z-FDDP**, **Fmoc-FPP**; 10 mM each) shown for: **A)** Boc-FR-NH_2 (retention time 7.49 min), **B)** Boc-FD-NH_2 (retention time 7.67 min), **C)** Z-FR-NH_2 (retention time 8.32 min), **D)** Boc-F-OH (retention time 9.53 min), **E)** Z-F-OH,(retention time 10.35 min), **F)** Boc-FW-NH_2 (retention time 10.79 min) and Fmoc-FR-NH_2 (retention time 12.09 min) **G)** Z-FW-NH_2 (retention time 11.27 min), **H)** Fmoc-F-OH (retention time 12.81 min), **I)** Fmoc-FW-NH_2 (retention time 13.25 min), in Supporting Figure S57, obtained from the LC-MS analysis.

Calculated m/z $[M+H]^+$: **Boc-FR-NH_2** 421.2558, **Boc-FD-NH_2** 380.1816, **Z-FR-NH_2** 455.2401, **Boc-F-OH** 266.1387, **Z-FOH** 300.1223, **Boc-FW-NH_2** 451.2340, **Z-FW-NH_2** 485.2183, **Fmoc-FR-NH_2** 543.2714, **Fmoc-F-OH** 388.1543, **Fmoc-FW-NH_2** 573.2496.

Observed m/z $[M+H]^+$: **Boc-FR-NH_2** 421.2562, **Boc-FD-NH_2** 380.1820, **Z-FR-NH_2** 455.2411, **Z-FOH** 300.1233, **Z-FW-NH_2** 485.2188, **Fmoc-FR-NH_2** 543.2713, **Fmoc-FOH** 388.1547, **Fmoc-FW-NH_2** 573.2506.

$[M+Na]^+$: **Boc-F-OH** 288.1208, **Boc-FW-NH_2** 473.2158.

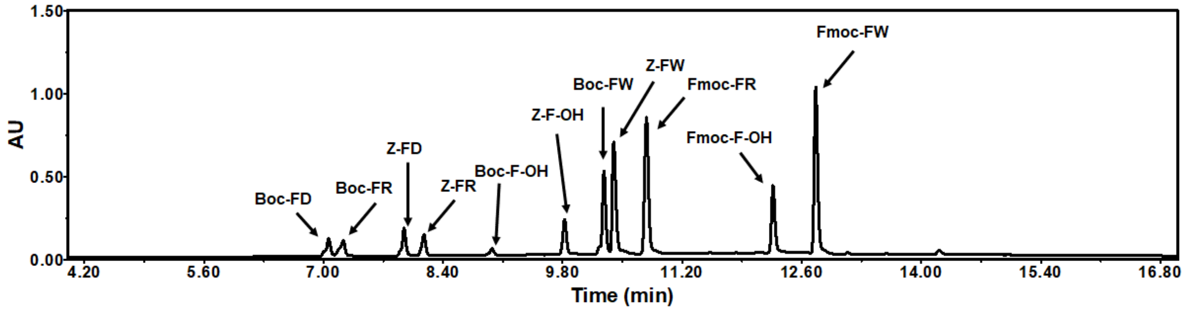

Figure S59. UPLC chromatograms of reactions of amide mixtures (R, W, D; 10 mM each) with amino acyl phosphate esters (**Boc-FEP**, **Z-FDDP**, **Fmoc-FPP**; 10 mM each) in 50% acetonitrile:0.6 M borate buffer at pH 9.1. Measurements were taken after 1 h.

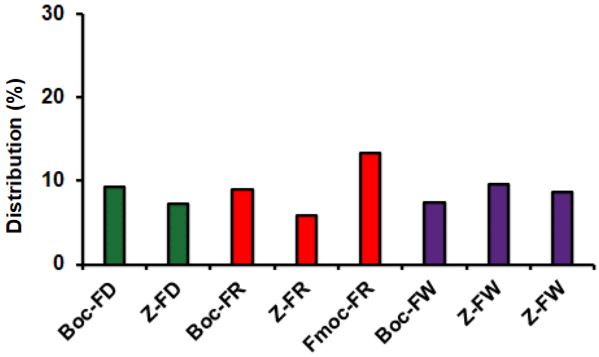

Figure S60. Bar graph showing product distribution from reactions of amide mixtures (R, W, D; 10 mM each) with amino acyl phosphate esters (**Boc-FEP**, **Z-FDDP**, **Fmoc-FPP**; 10 mM each) in 50% acetonitrile:0.6 M borate buffer at pH 9.1. Measurements were taken after 1 h.

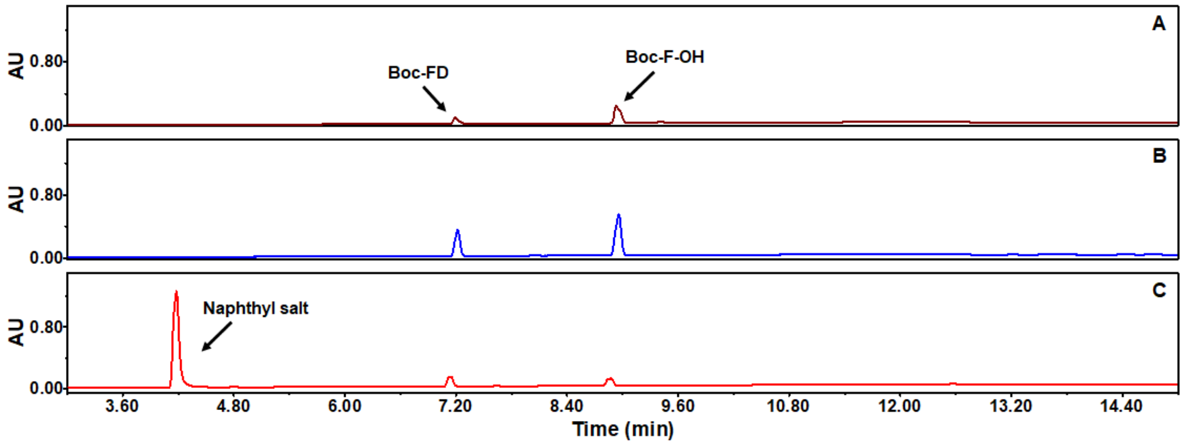

Figure S61. UPLC chromatograms of reactions of 10 mM D with Boc-protected amino acyl phosphate esters **A) Boc-FDDP**, **B) Boc-FEP**, **C) Boc-FNP**; 10 mM each in 0.6 M borate buffer at pH 9.1. Measurements were taken after 1 hour.

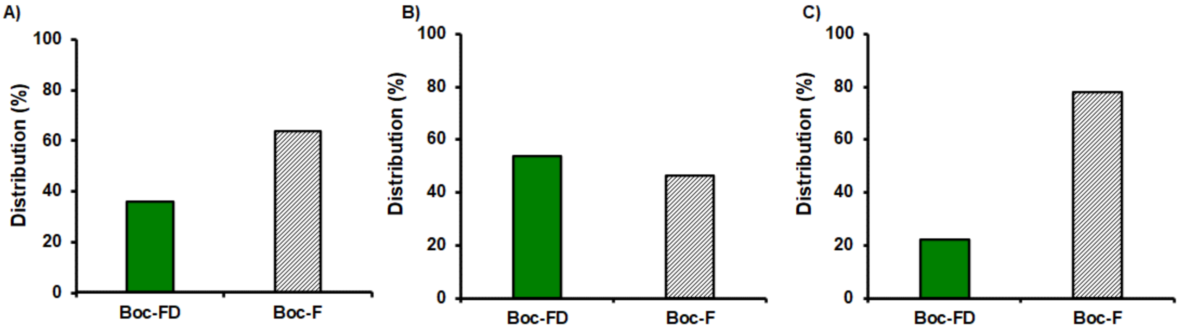

Figure S62. Bar graphs showing peptide coupling from reactions of 10 mM D with Boc-protected amino acyl phosphate esters **A) Boc-FEP**, **B) Boc-FNP**, **C) Boc-FDDP** (10 mM each) in 0.6 M borate buffer at pH 9.1. Measurements were taken after 1 hour.

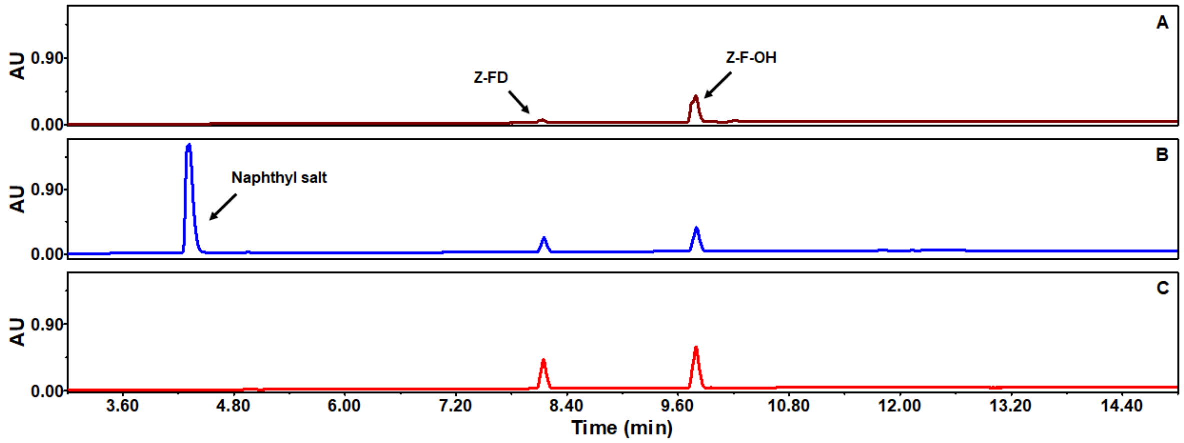

Figure S63. UPLC chromatograms of reactions of 10 mM D with Z-protected amino acyl phosphate esters **A) Boc-FDDP**, **B) Z-FNP**, **C) Z-FEP**; 10 mM each) in 0.6 M borate buffer at pH 9.1. Measurements were taken after 1 h.

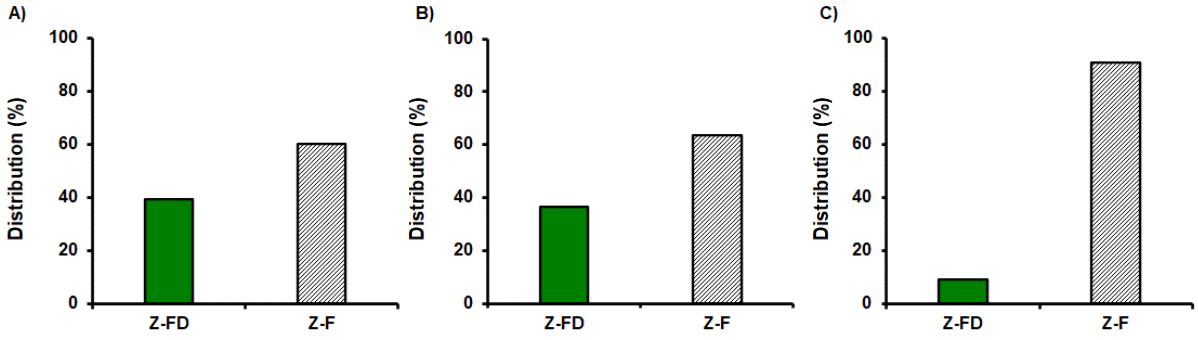

Figure S64. Bar graphs showing peptide coupling from reactions of 10 mM D with Z-protected amino acyl phosphate esters **A) Z-FEP**, **B) Z-FNP**, **C) Boc-FDDP** (10 mM each) in 0.6 M borate buffer at pH 9.1. Measurements were taken after 1 h.

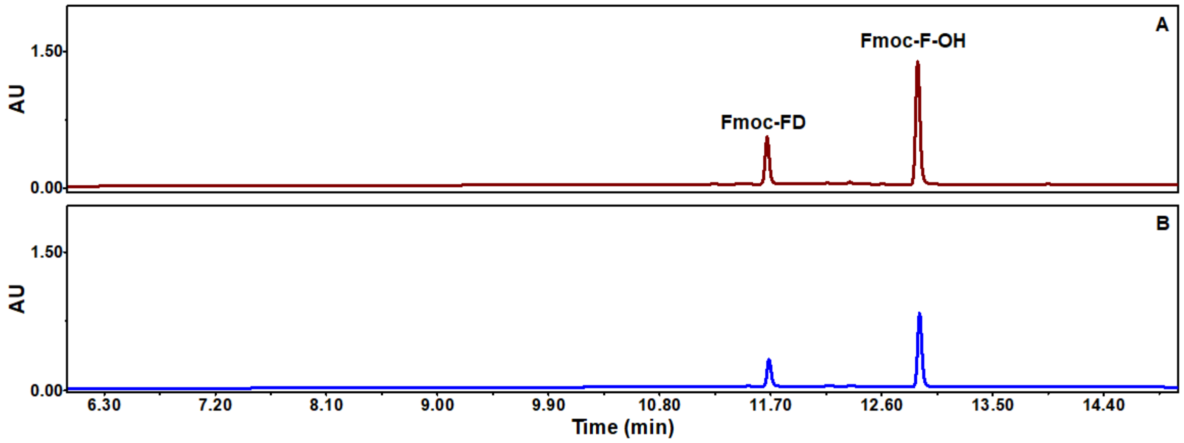

Figure S65. UPLC chromatograms of reactions of 10 mM D with Fmoc-protected amino acyl phosphate esters **A) Fmoc-FEP**, **B) Fmoc-FPP**, (10 mM each) in 0.6 M borate buffer at pH 9.1. Measurements were taken after 1 h.

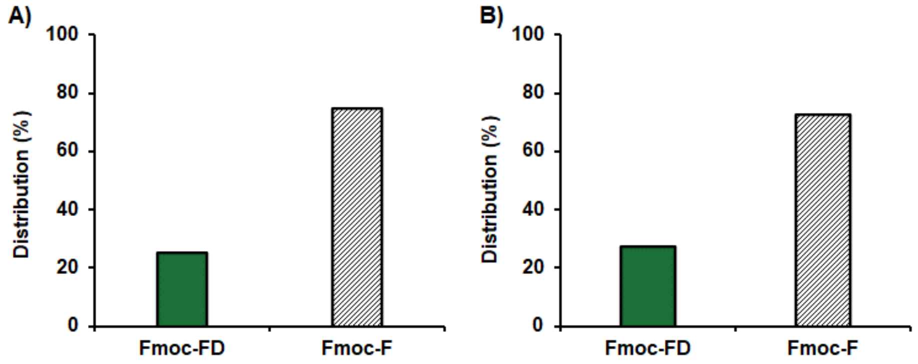

Figure S66. Bar graphs showing peptide coupling from reactions of 10 mM D with Fmoc-protected amino acyl phosphate esters **A) Fmoc-FEP**, **B) Fmoc-FPP**, (10 mM each) in 0.6 M borate buffer at pH 9.1. Measurements were taken after 1 h.

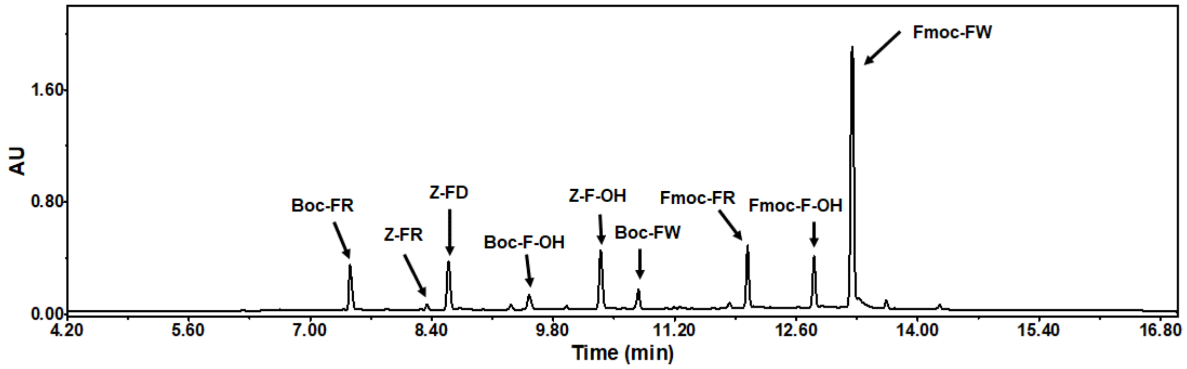

Figure S67. UPLC chromatograms of reactions of amide mixtures (R, W, D; 10 mM each) with amino acyl phosphate esters (**Boc-FDDP**, **Z-FEP**, **Fmoc-FPP**; 10 mM each) in 0.6 M borate buffer at pH 9.1. Measurements were taken after 1 h.

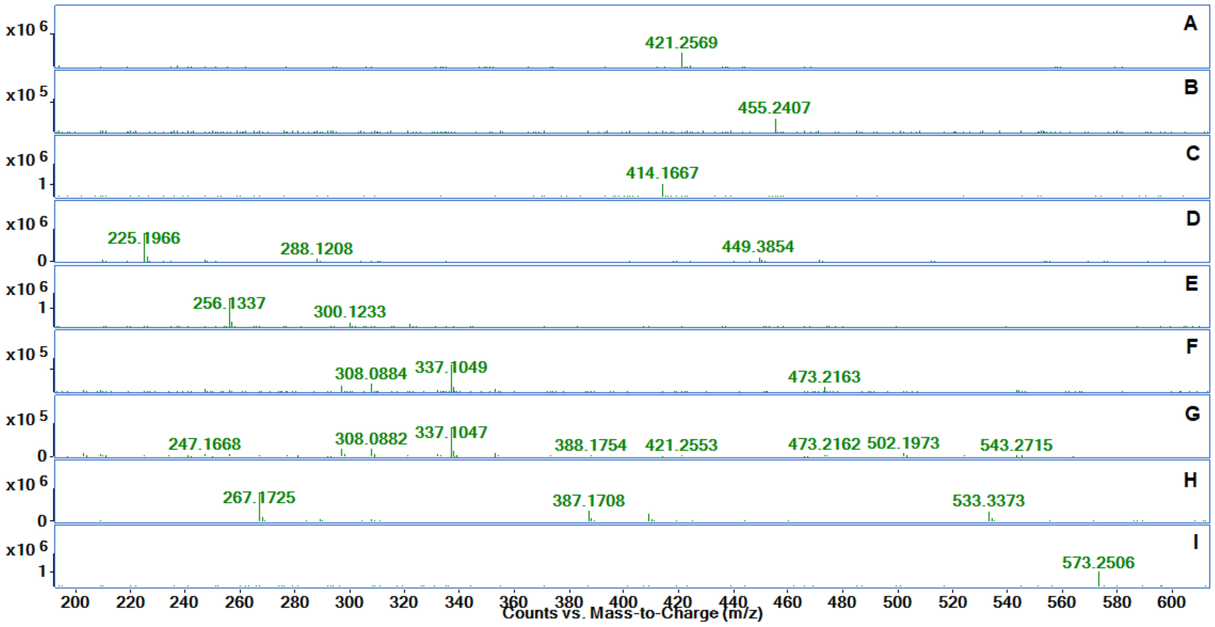

Figure S68. Mass spectra of peptide reactions of amide mixtures (R, W, D; 10 mM each) with amino acyl phosphate esters (**Boc-FDDP**, **Z-FEP**, **Fmoc-FPP**; 10 mM each) shown for: **A)** Boc-FR-NH_2 (retention time 7.49 min), **B)** Z-FR-NH_2 (retention time 7.67 min), **C)** Z-FD-NH_2 (retention time 8.59 min), **D)** Boc-F-OH (retention time 9.53 min), **E)** Z-F-OH (retention time 10.35 min), **F)** Boc-FW-NH_2 (retention time 10.79 min), **G)** Fmoc-FR-NH_2 (retention time 12.09 min), **H)** Fmoc-F-OH (retention time 12.81 min), **I)** Fmoc-FW-NH_2 (retention time 13.25 min), in Supporting Figure S67, obtained from the LC-MS analysis.

Calculated m/z $[M+H]^+$: **Boc-FR-NH_2** 421.2558, **Z-FR-NH_2** 455.2401, **Z-FD-NH_2** 414.1660, **Boc-FOH** 266.1387, **Z-FOH** 300.1223, **Boc-FW-NH_2** 451.2340, **Fmoc-FR-NH_2** 543.2714, **Fmoc-FOH** 388.1543, **Fmoc-FW-NH_2** 573.2496.

Observed m/z $[M+H]^+$: **Boc-FR-NH_2** 421.2562, **Z-FR-NH_2** 455.2411, **Z-FD-NH_2** 414.1667, **Z-FOH** 300.1233, **Fmoc-FR-NH_2** 543.2715, **Fmoc-FOH** 387.1708, **Fmoc-FW-NH_2** 573.2506.

[M+Na]+: **Boc-F-OH** 288.1208, **Boc-FW-NH_2** 473.2163.

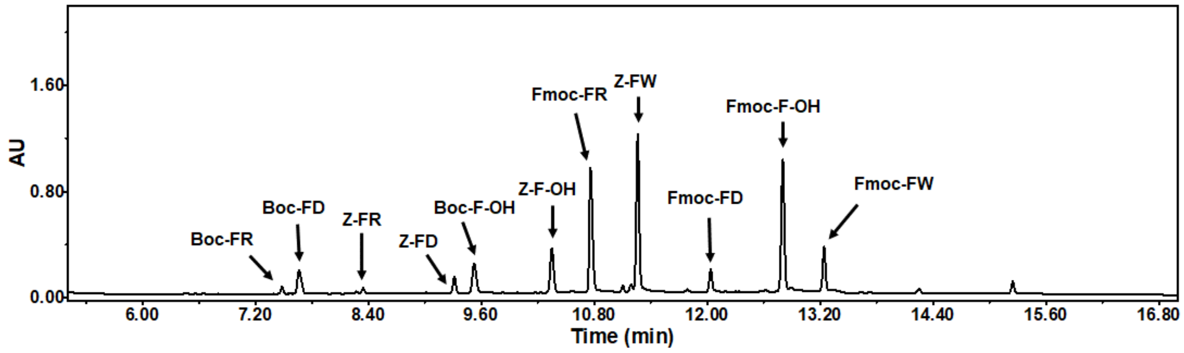

Figure S69. UPLC chromatograms of reactions of amide mixtures (R, W, D; 10 mM each) with amino acyl phosphate esters (**Boc-FEP**, **Z-FNP**, **Fmoc-FEP**; 10 mM each) in 0.6 M borate buffer at pH 9.1. Measurements were taken after 1 h.

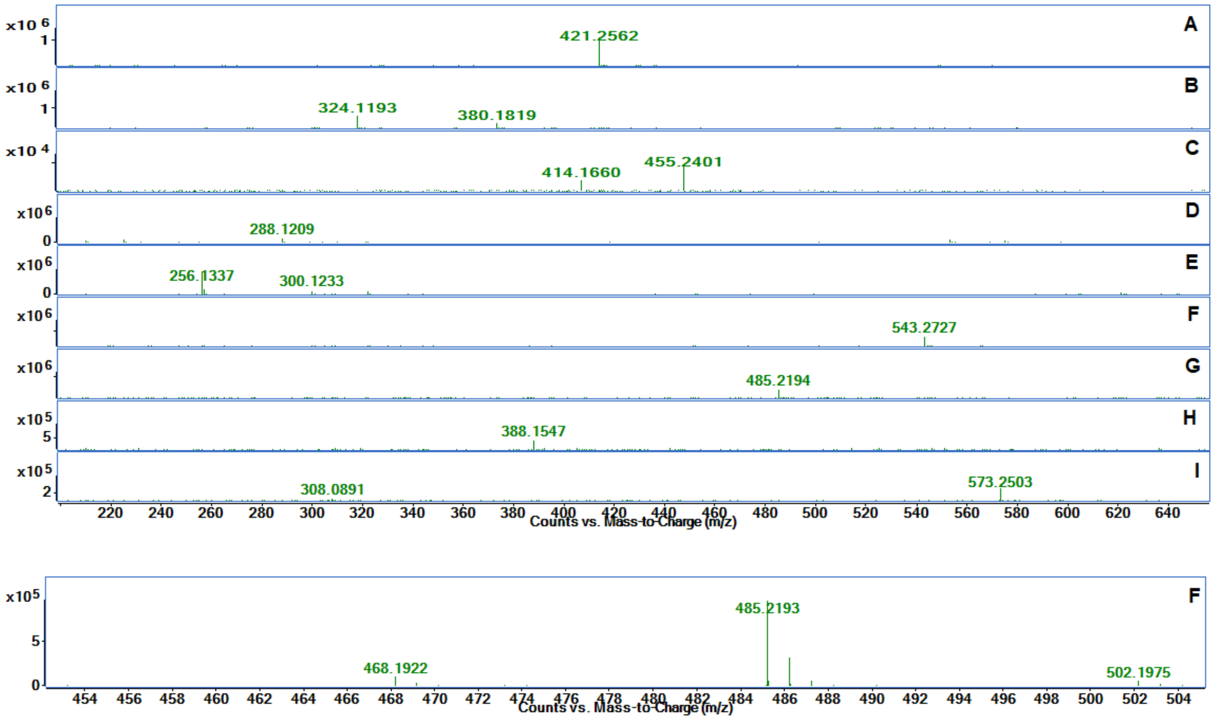

Figure S70. Mass spectra of peptide reactions of amide mixtures (R, W, D; 10 mM each) with amino acyl phosphate esters (**Boc-FEP**, **Z-FNP**, **Fmoc-FEP**; 10 mM each) shown for: **A)** Boc-FR-NH_2 (retention time 7.49 min), **B)** Boc-FD-NH_2 (retention time 7.67 min), **C)** Z-FR-NH_2 (retention time 8.34 min) and Z-FD-NH_2 (retention time 9.31 min) **D)** Boc-F-OH (retention time 9.53 min), **E)** Z-F-OH (retention time 10.35 min), **F)** Z-FW-OH (retention time 10.35 min), **F)** Fmoc-FR-NH_2 (retention time 10.71 min), **G)** Z-FW-NH_2 (retention time 11.26min) **H)** Fmoc-F-OH (retention time 12.81 min), **I**) Fmoc-FW-NH_2 (retention time 13.25 min), **F**) Fmoc-FD-NH_2 (retention time 12.04 min) in Supporting Figure S69, obtained from the LC-MS analysis.
Calculated m/z $[M+H]^+$: **Boc-FR-NH_2** 421.2558, **Boc-FD-NH_2** 380.1816, **Z-FR-NH_2** 455.2401, **Z-FD-NH_2** 414.1660, **Boc-F-OH** 266.1387, **Z-FOH** 300.1223, **Z-FD-NH_2** 485.2183, **Fmoc-FR-NH_2** 543.2714, **Fmoc-F-OH** 388.1543, **Fmoc-FW-NH_2** 573.2496, **Fmoc-FD-NH_2** 502.1973.
Observed m/z $[M+H]^+$: **Boc-FR-NH_2** 421.2565, **Boc-FD-NH_2** 380.1819, **Z-FR-NH_2** 455.2401, **Z-FD-NH_2** 414.1660, **Z-F-OH** 300.1233, **Z-FD-NH_2** 485.2194, **Fmoc-FR-NH_2** 543.2727, **Fmoc-FOH** 388.1547, **Fmoc-FW-NH_2** 573.2503, **Fmoc-FD-NH_2** 502.1975.
$[M+Na]^+$: **Boc-F-OH** 288.1209.

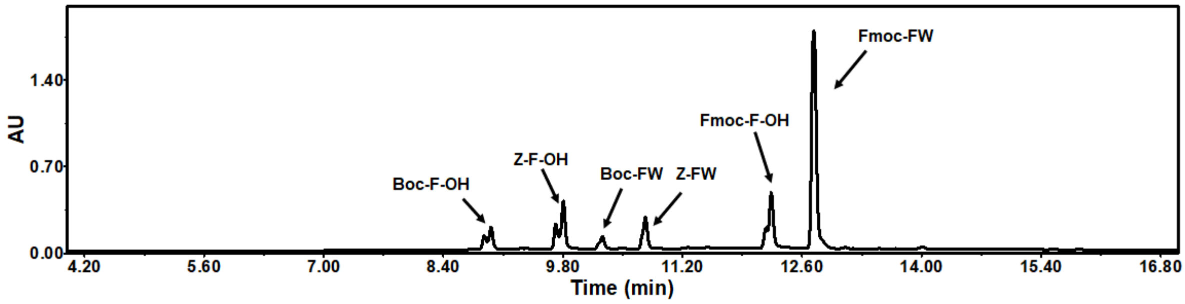

Figure S71. UPLC chromatogram of reactions of 10 mM W with amino acyl phosphate esters mixtures (**Boc-FEP**, **Z-FDDP**, **Fmoc-FPP**; 10 mM each) in 0.6 M borate buffer at pH 9.1. Measurements were taken after 1 h.

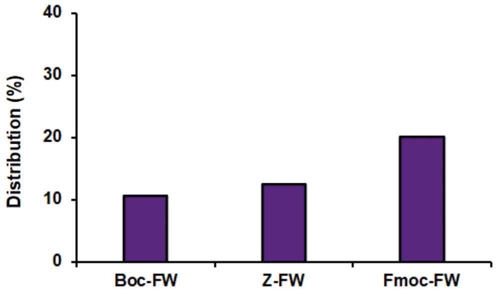

Figure S72. Bar graph showing peptide coupling of reactions of 10 mM W with amino acyl phosphate esters mixtures (**Boc-FEP**, **Z-FDDP**, **Fmoc-FPP**; 10 mM each) in 0.6 M borate buffer at pH 9.1. Measurements were taken after 1 h.

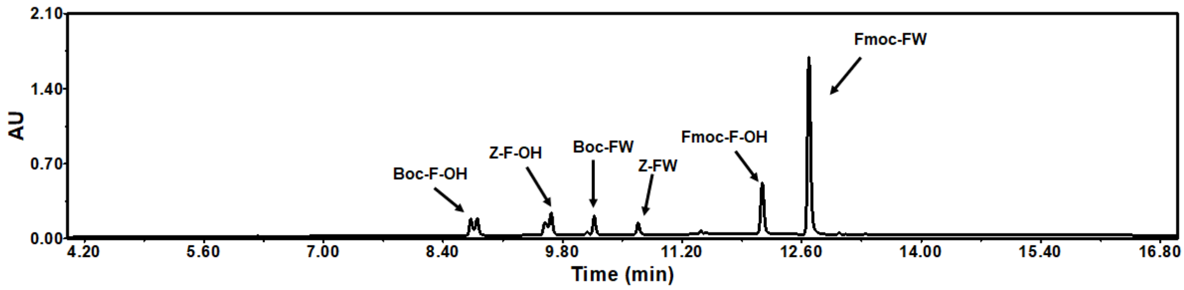

Figure S73. UPLC chromatogram of reactions of 10 mM W with amino acyl phosphate esters mixtures (**Boc-FDDP**, **Z-FEP**, **Fmoc-FPP**; 10 mM each) in 0.6 M borate buffer at pH 9.1. Measurements were taken after 1 h.

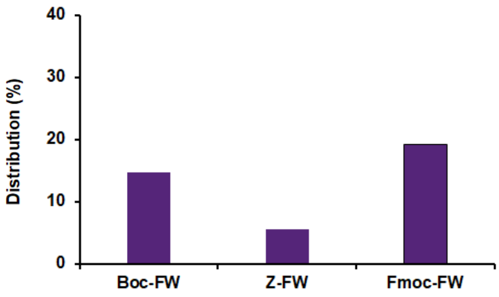

Figure S74. Bar graph showing peptide coupling of reactions of 10 mM W with amino acyl phosphate esters mixtures (**Boc-FDDP**, **Z-FEP**, **Fmoc-FPP**; 10 mM each) in 0.6 M borate buffer at pH 9.1. Measurements were taken after 1 h.

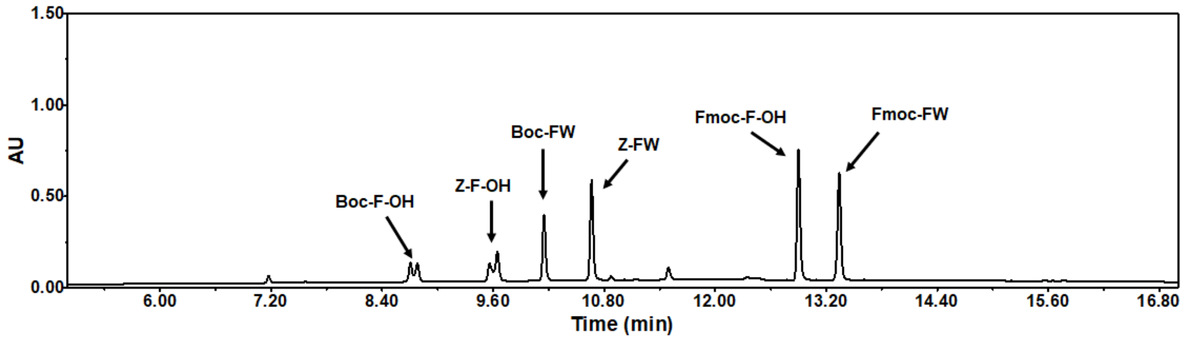

Figure S75. UPLC chromatogram of reactions of 10 mM W with amino acyl phosphate esters mixtures (**Boc-FEP**, **Z-FNP**, **Fmoc-FEP**; 10 mM each) in 0.6 M borate buffer at pH 9.1. Measurements were taken after 1 h.

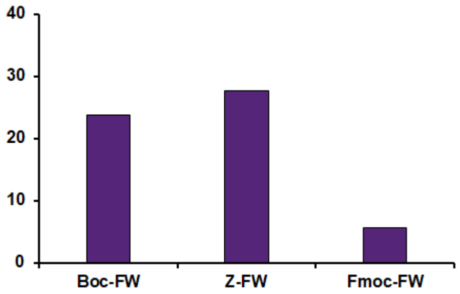

Figure S76. Bar graph showing peptide coupling of reactions of 10 mM W with amino acyl phosphate esters mixtures (**Boc-FDDP**, **Z-FNP**, **Fmoc-FEP**; 10 mM each) in 0.6 M borate buffer at pH 9.1. Measurements were taken after 1 h.

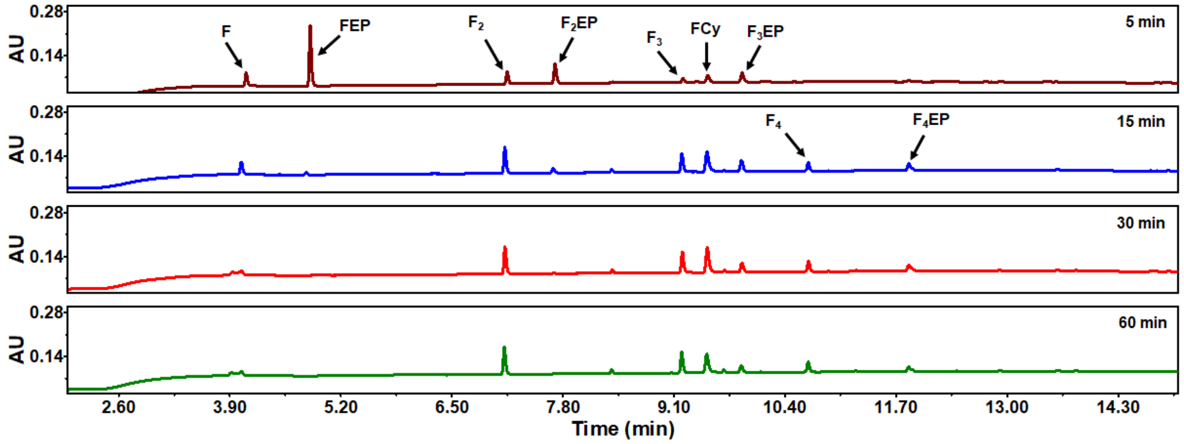

Figure S77. Time-dependent UPLC chromatograms of 10 mM **FEP** in 0.6M MOPS buffer, pH 8.0.

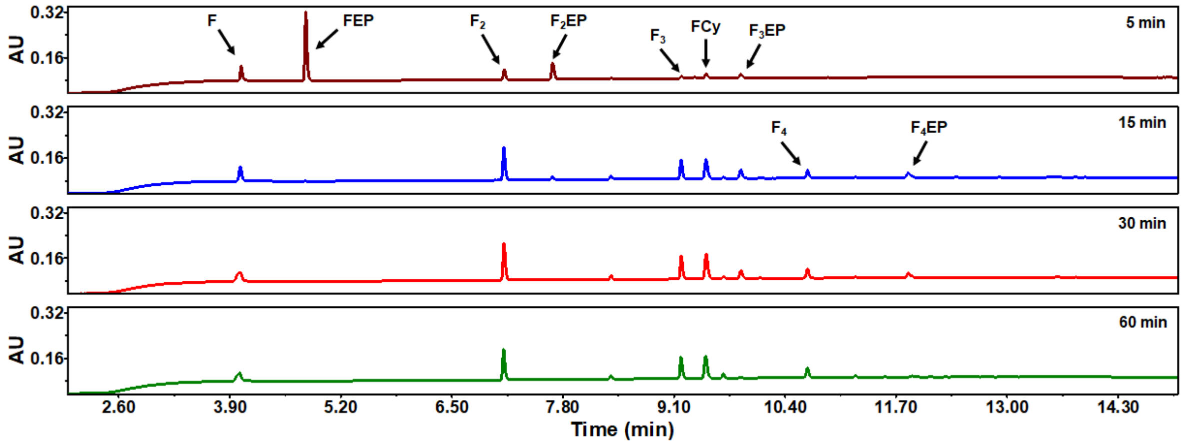

Figure S78. Time-dependent UPLC chromatograms of 10 mM **FEP** in 1.2 M MOPS buffer, pH 8.0

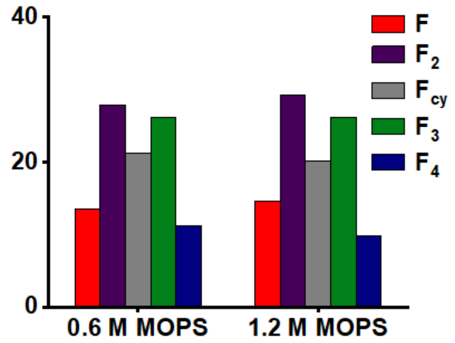

Figure S79. Bar graph comparing the oligomer distribution obtained from 10 mM **FEP** in 0.6 M and 1.2 M MOPS buffer (pH 8.0).

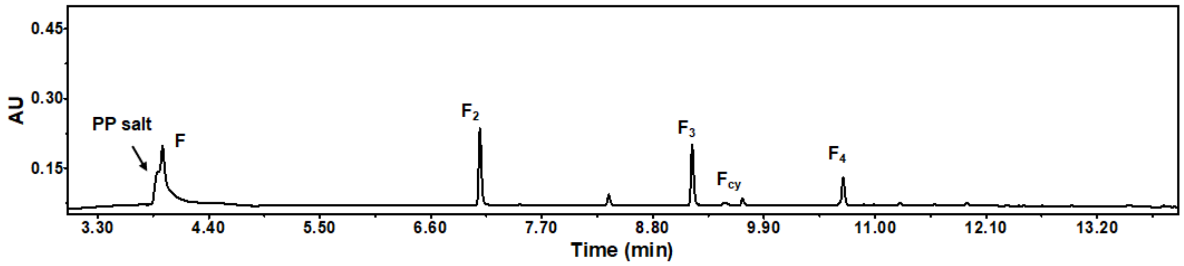

Figure S80. UPLC chromatograms of 10 mM **FEP** in 0.6 M MOPS buffer, pH 8.0. The measurement was done after 1 hour of reaction.

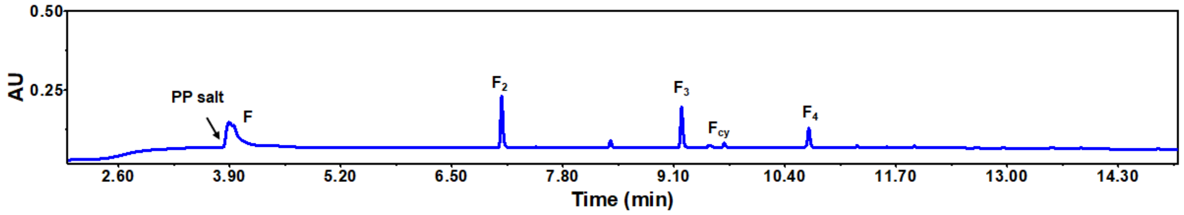

Figure S81. Time-dependent UPLC chromatograms of 10 mM **FEP** in 1.2 M MOPS buffer, pH 8.0. The measurement was done after 1 hour of reaction.

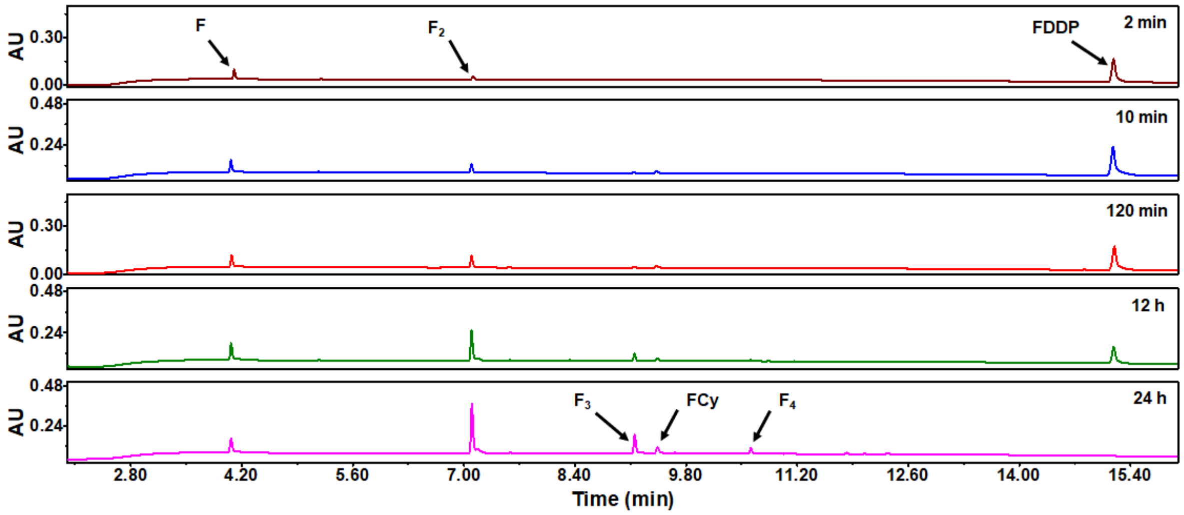

Figure S82. Time-dependent UPLC chromatograms of 10 mM **FDDP** in 1.2 M MOPS buffer, pH 8.0.

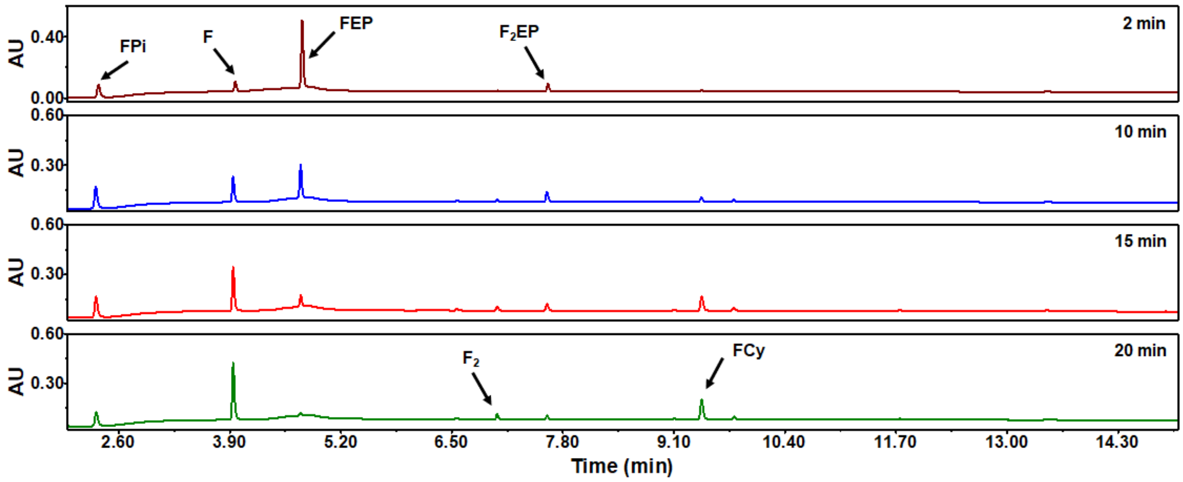

Figure S83. Time-dependent UPLC chromatograms of 10 mM **FEP** in 0.6 M PBS buffer, pH 8.0.

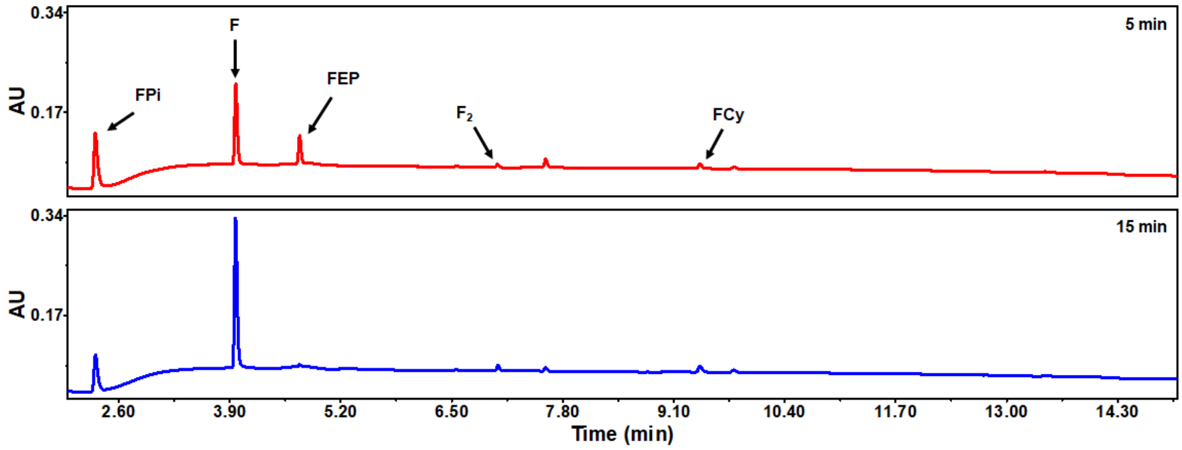

Figure S84. Time-dependent UPLC chromatograms of 10 mM **FEP** in 1.2 M PBS buffer, pH 8.0.

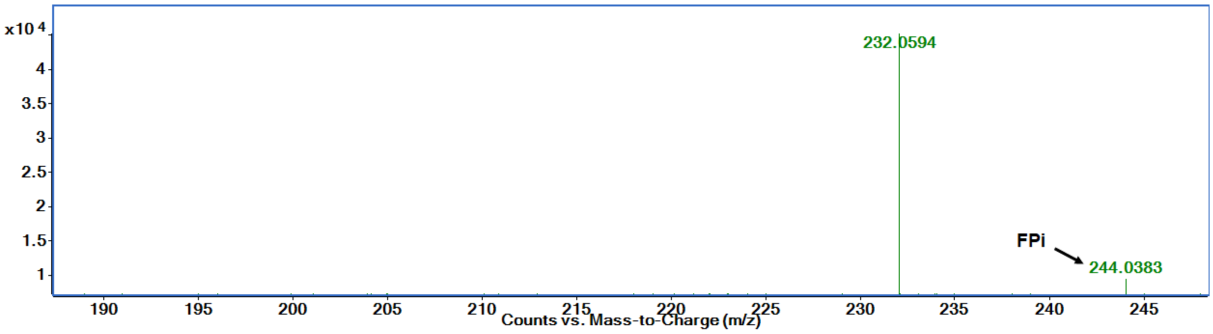

Figure S85. Mass spectra of the formed **FPi** (retention time 2.42 min) from reaction between 10 mM **FEP** in 1.2 M PBS buffer, pH 8.0. The observed mass is consistent with that reported previously by Ghadiri and co-workers.[4]
Calculated m/z $[M-H]^-$: **FPi** 244.0380.
Observed m/z $[M-H]^-$: **FPi** 244.0383.

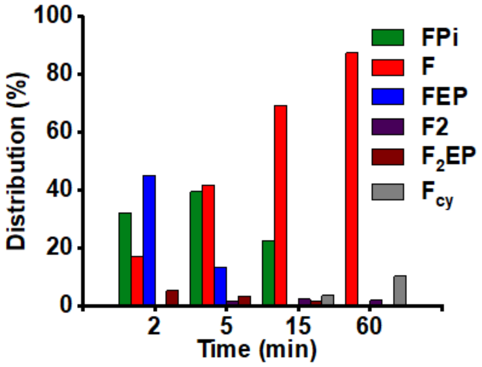

Figure S86. Bar graph showing product distribution from the reaction of 10 mM **FEP** in 1.2 M PBS buffer, pH 8.0.

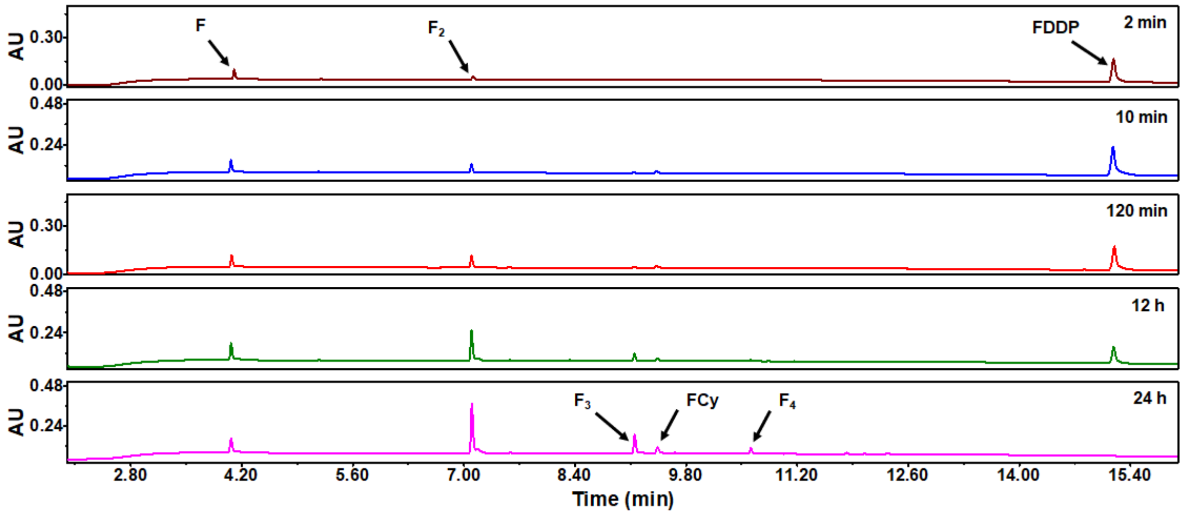

Figure S87. Time-dependent UPLC chromatograms of 10 mM **FDDP** in 1.2 M PBS buffer, pH 8.0.

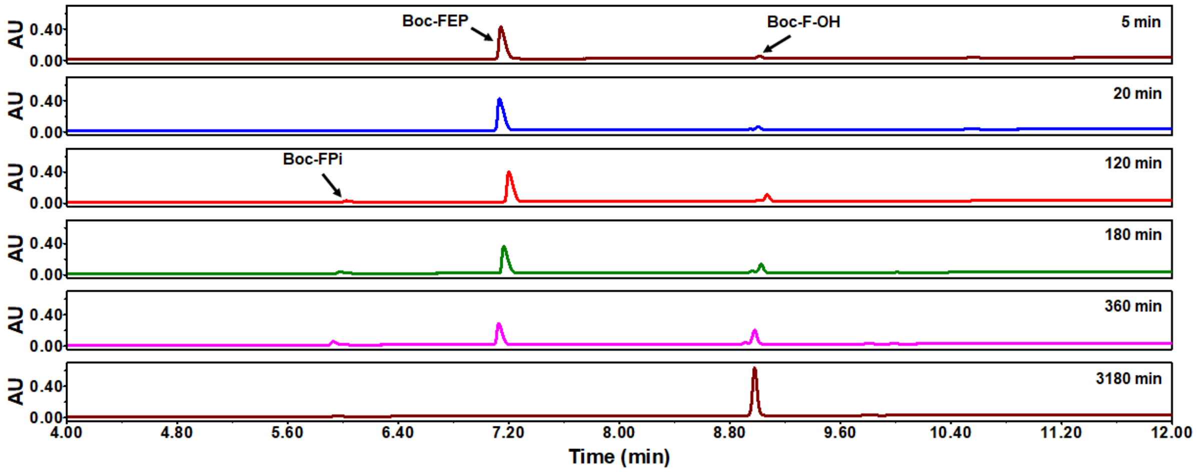

Figure S88: Time-dependent UPLC chromatograms of 10 mM **Boc-FEP** in 1.2 M PBS buffer, pH 8.0.

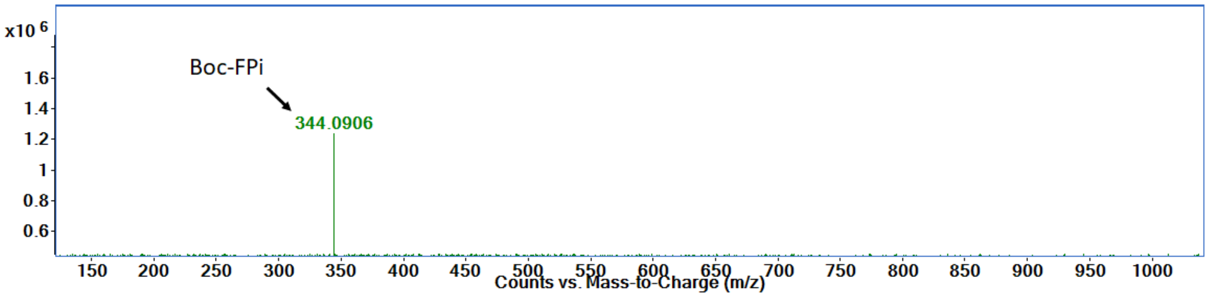

Figure S89. Mass spectra of the formed **Boc-FPi** (retention time 5.9 min) from reaction between 10 mM Boc-FEP in 1.2 M PBS buffer, pH 8.0.
Calculated m/z [M-H]-: **Boc-FPi** 344.0905.
Observed m/z [M-H]-: **Boc-FPi** 344.0906.

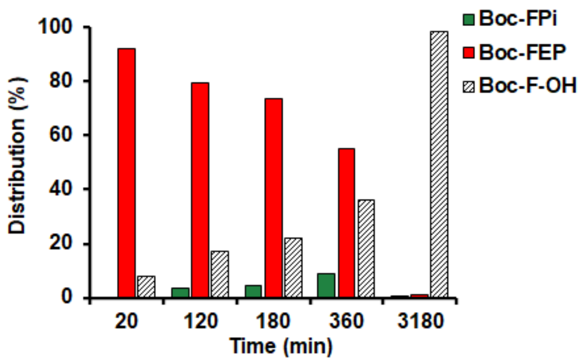

Figure S90. Bar graph showing product distribution from the reaction of 10 mM **Boc-FEP** in 1.2 M PBS buffer, pH 8.0.

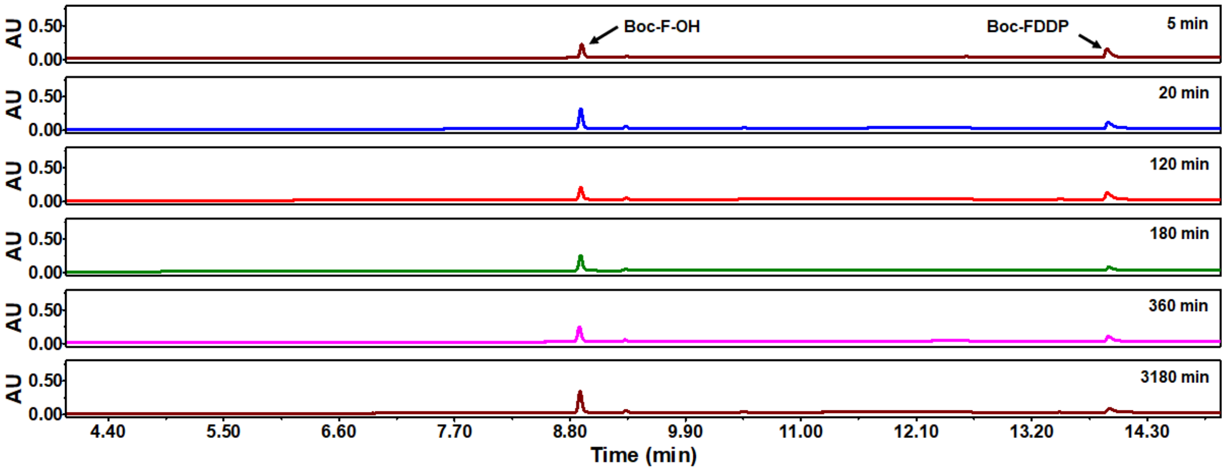

Figure S91: Time-dependent UPLC chromatograms of 10 mM **Boc-FDDP** in 1.2 M PBS buffer, pH 8.0.

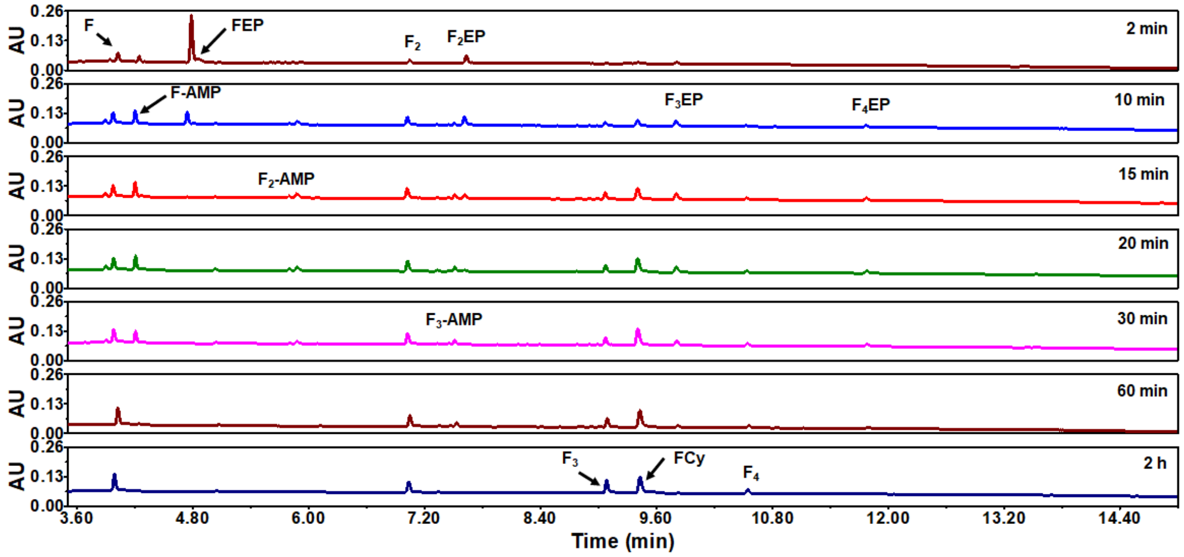

Figure S92. Time-dependent UPLC chromatograms of 10 mM **FEP** incubated with 100 mM AMP in 0.1 M HEPES buffer, pH 8.0.

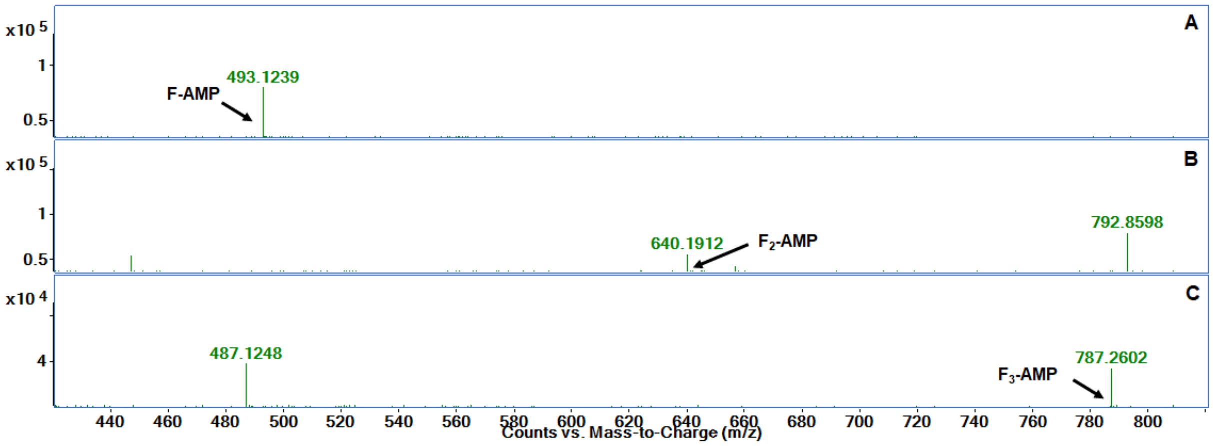

Figure S93. Mass spectra showing the formed **F-AMP** oligomer species from reaction between 10 mM **FEP** and 100 mM AMP in 0.1 M HEPES buffer, pH 8.0, shown for: **A)** F-AMP (retention time 4.26 min), **B)** F_2-AMP (retention time 5.94 min), **C)** F_3-AMP (retention time 7.60 min) in Supporting Figure S92, obtained from the LC-MS analysis.
Calculated m/z $[M-H]^-$: **F-AMP** 493.1242, **F_2-AMP** 640.1926, **F_3-AMP** 787.2611.
Observed m/z $[M-H]^-$: **F-AMP** 493.1239, **F_2-AMP** 640.1912, **F_3-AMP** 787.2602.

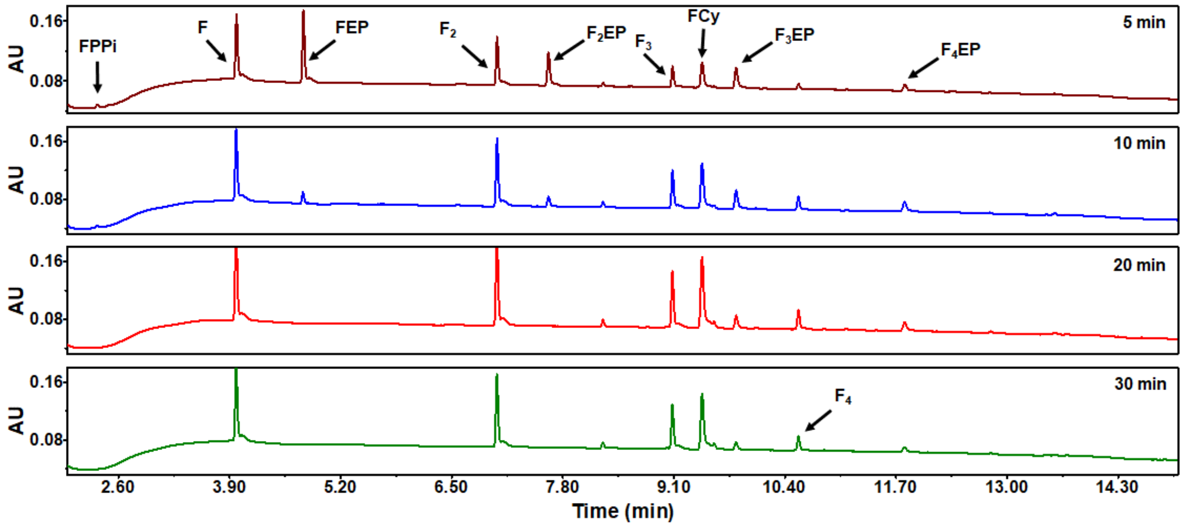

Figure S94. Time-dependent UPLC chromatograms of 10 mM **FEP** incubated with 100 mM pyrophosphate in 0.1 M HEPES buffer, pH 8.0.

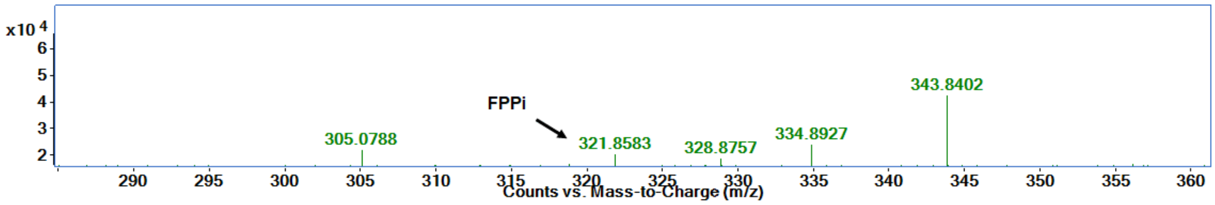

Figure S95. Mass spectra of the formed **F-PPi** (retention time 2.45 min) from reaction between 10 mM **FEP** and 100 mM pyrophosphate ($Na_2H_2P_2O_7$) in 0.1 M HEPES buffer, pH 8.0.
Calculated m/z $[M-H]^-$: **FPPi** 321.9898.
Observed m/z $[M-H]^-$: **FPPi** 321.8583.

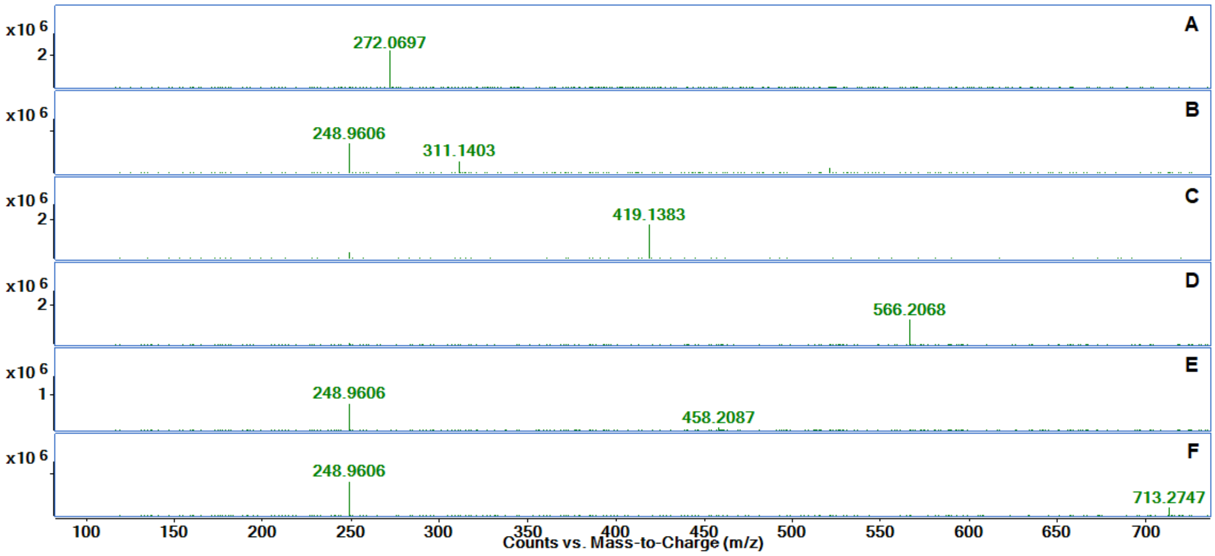

Figure S96. Mass spectra showing the formed oligomers from reaction between 10 mM **FEP** and pyrophosphate in 0.1 M HEPES buffer, pH 8.0, shown for: **A) FEP** (retention time 4.78 min), **B) F_2** (retention time 7.06 min), **C) F_2EP** (retention time 7.60 min), **D) F_3EP** (retention time 9.77 min), **E)** F_3 (retention time 9.11 min), **F) F_4EP** (retention time 11.71 min) in Supporting Figure S94, obtained from the LC-MS analysis.

Calculated m/z $[M\text{-}H]^-$: **FEP** 272.0688, **F_2** 311.1401, **F_2EP** 419.1372, **F_3EP** 566.2056, **F_3** 458.2085, **F_4EP** 713.2740.

Observed m/z $[M\text{-}H]^-$: **FEP** 272.0697, **F_2** 311.1403, **F_2EP** 419.1383, **F_3EP** 566.2068, **F_3** 458.2087, **F_4EP** 713.2747.

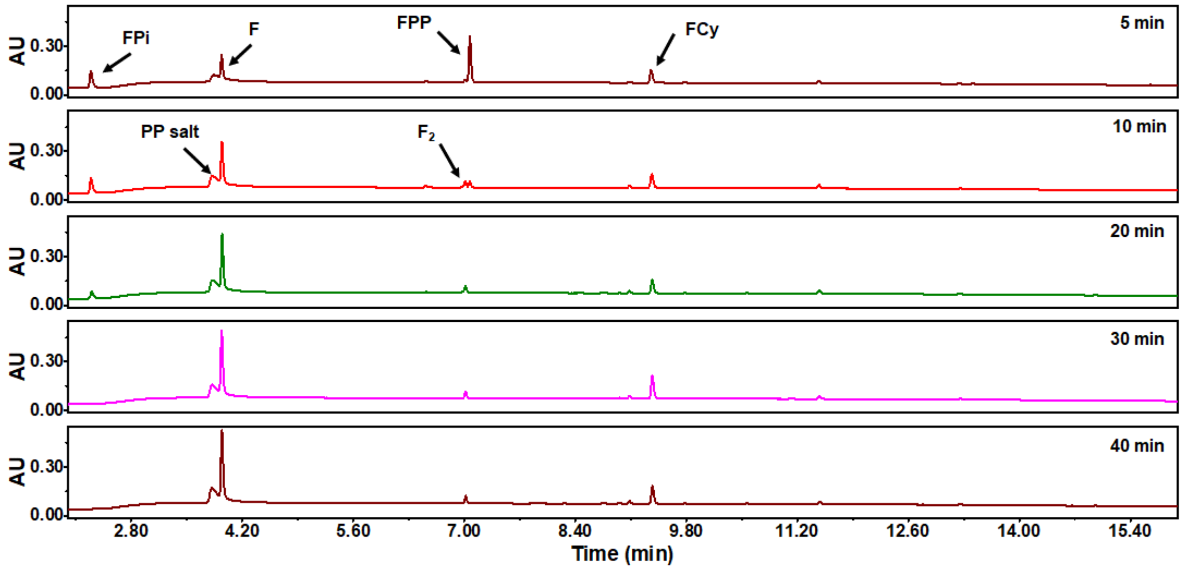

Figure S97. Time-dependent UPLC chromatograms of 10 mM **FPP** in 0.2 M PBS buffer, pH 8.0.

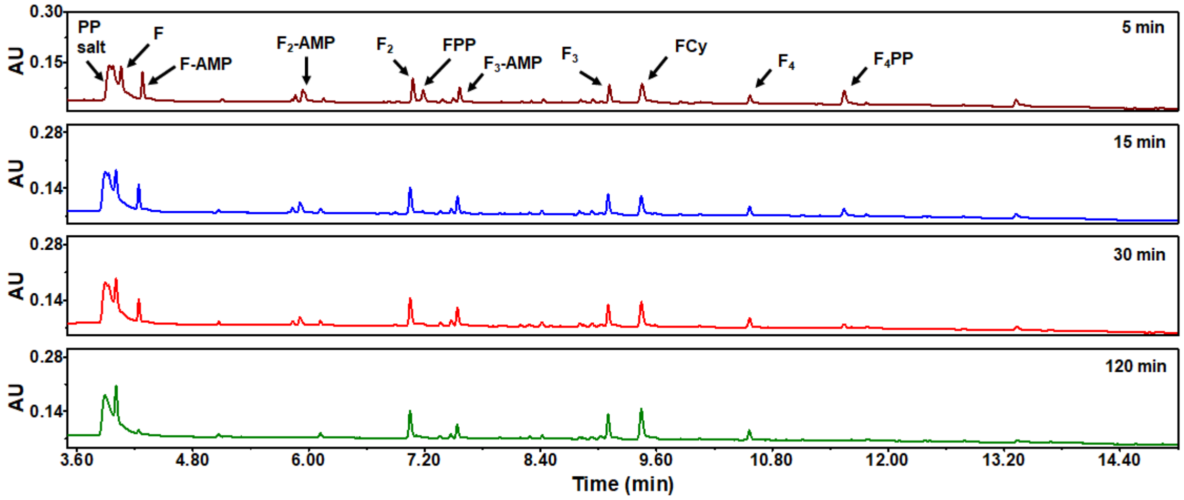

Figure S98. Time-dependent UPLC chromatograms of 10 mM **FPP** incubated with 100 mM AMP in 0.1 M HEPES buffer, pH 8.0.

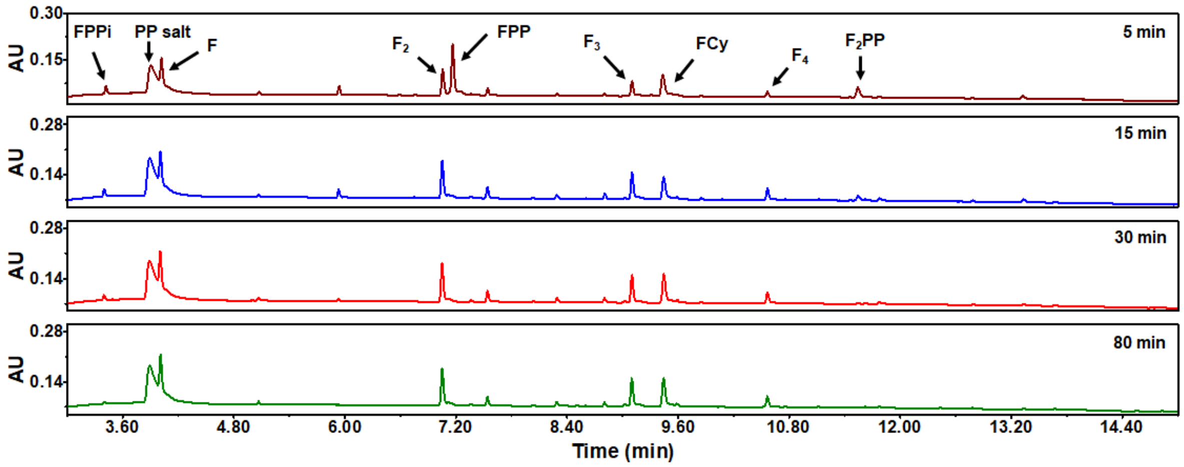

Figure S99. Time-dependent UPLC chromatograms of 10 mM **FPP** incubated with 100 mM pyrophosphate in 0.1 M HEPES buffer, pH 8.0.

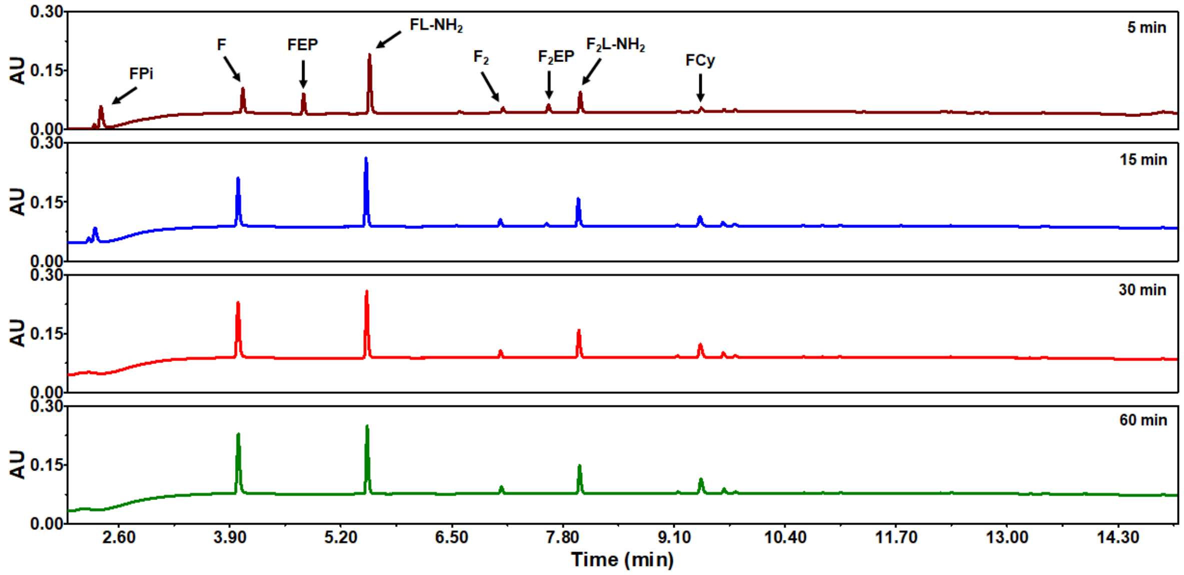

Figure S100. Time-dependent UPLC chromatograms of reaction between 10 mM **FEP** and 10 mM L-NH_2 in 0.6 M PBS buffer, pH 8.0.

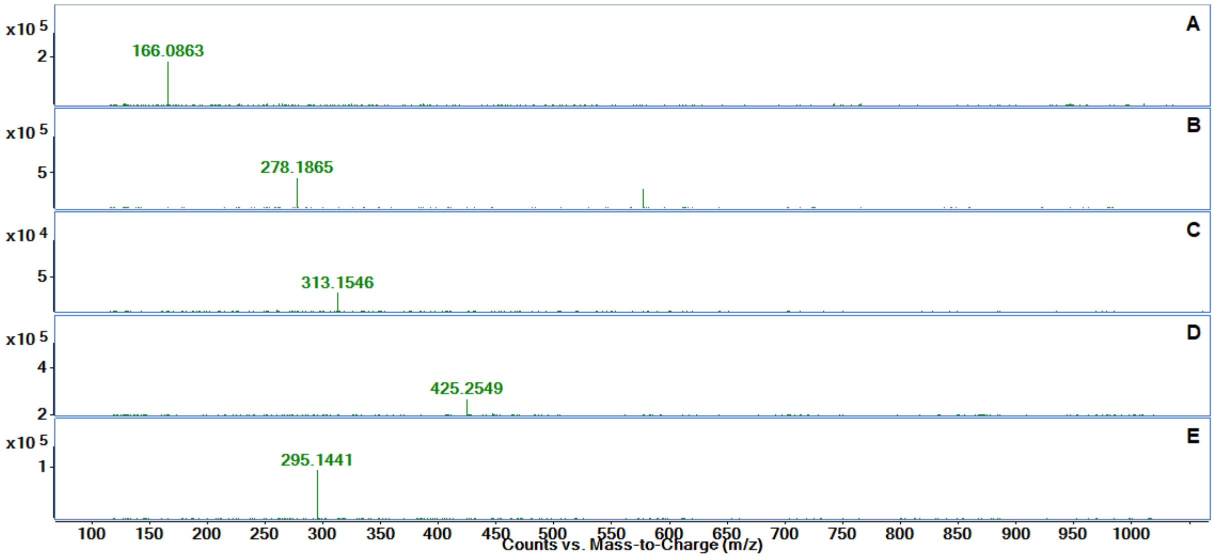

Figure S101. Mass spectra of mixed oligomers formed from the reaction between 10 mM **FEP** and 10 mM L-NH_2 in 0.6 M PBS buffer, pH 8.0, shown for: **A)** **F** (retention time 4.04 min), **B)** **FL-NH_2** (retention time 5.52 min), **C)** **F_2** (retention time 7.10 min), **D)** **F_2L-NH_2** (retention time 7.98 min), **E)** **F_{cy}** (retention time 9.40 min) in Supporting Figure S100, obtained from the LC-MS analysis.

Calculated m/z $[M+H]^+$: **F** 166.0863, **FL-NH_2** 278.1863, **F_3** 313.1547, **F_2L-NH_2** 425.2547, **F_{cy}** 295.1441.

Observed m/z $[M+H]^+$: **F** 166.0863, **FL-NH_2** 278.1865, **F_3** 313.1546, **F_2L-NH_2** 425.2549, **F_{cy}** 295.1441.

4 Characterization of compounds

4.1 Characterization of Boc/Cbz/Fmoc-aminoacyl phosphate esters by NMR

[i] (S)-2-((tert-butoxycarbonyl)amino)-3-phenylpropanoic (ethyl phosphoric) anhydride: The title compound was prepared according to the general procedure [I, II & III] as white hygroscopic solid in 45% yield. **^{1}H NMR (300 MHz, Chloroform-*d*)** *δ* (ppm) 7.20 (tt, *J* = 9.6, 5.2 Hz, 5H), 5.75 – 5.64 (m, 1H), 4.52 (td, *J* = 8.7, 4.6 Hz, 1H), 3.98 (p, *J* = 7.2 Hz, 2H), 3.24 (dd, *J* = 14.1, 4.6 Hz, 1H), 2.97 (dd, *J* = 14.1, 9.2 Hz, 1H), 1.43 – 1.01 (m, 13H); **^{31}P NMR (122 MHz, Chloroform-*d*)** *δ* (ppm) -6.29.

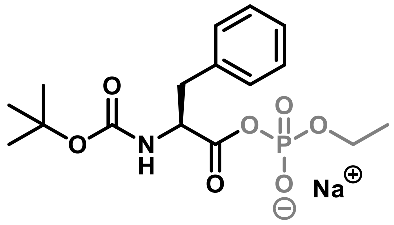

Boc-FEP

[ii] (S)-2-((tert-butoxycarbonyl)amino)-3-phenylpropanoic (phenyl phosphoric) anhydride:

The title compound was prepared according to the general procedure [I, II & III] as white hygroscopic solid in 55% yield. **^{1}H NMR (300 MHz, DMSO-d_6)** *δ* (ppm) 7.33 – 7.05 (m, 10H), 7.04 – 6.97 (m, 1H), 4.12 (ddd, *J* = 10.7, 8.7, 3.7 Hz, 1H), 2.95 (dd, *J* = 13.9, 3.8 Hz, 1H), 2.72 (dd, *J* = 13.9, 10.8 Hz, 1H), 1.30 (s, 9H); **^{31}P NMR (122 MHz, Chloroform-*d*)** δ (ppm) -12.41.

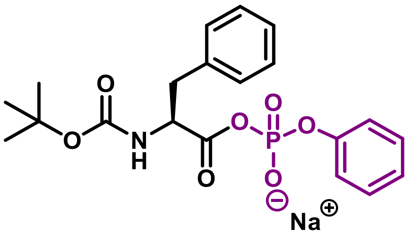

Boc-FPP

[iii] (S)-2-((tert-butoxycarbonyl)amino)-3-phenylpropanoic (naphthyl phosphoric) anhydride:

The title compound was prepared according to the general procedure [I, II & III] as white hygroscopic solid in 45% yield. **^{1}H NMR (300 MHz, DMSO-d$_6$)** δ (ppm) 7.88 – 7.72 (m, 2H), 7.72 – 7.58 (m, 1H), 7.47 – 7.27 (m, 3H), 7.27 – 7.09 (m, 6H), 4.14 (ddd, *J* = 10.7, 8.7, 3.7 Hz, 1H), 2.96 (dd, *J* = 13.7, 3.6 Hz, 1H), 2.82 – 2.67 (m, 1H), 1.29 (d, *J* = 3.3 Hz, 9H); **^{31}P NMR (162 MHz, Chloroform-*d*)** δ (ppm) -12.37.

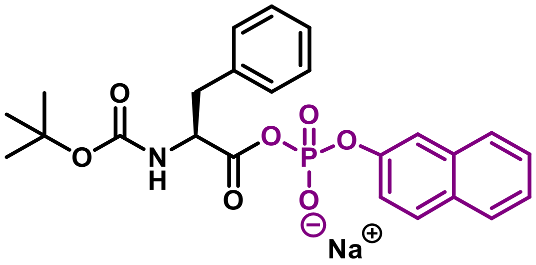

Boc-FNP

[iv] (S)-2-((tert-butoxycarbonyl)amino)-3-phenylpropanoic (dodecyl phosphoric) anhydride: The title compound was prepared according to the general procedure [I, II & III] as white hygroscopic solid in 60% yield. **^{1}H NMR (300 MHz, DMSO-d$_6$)** δ (ppm) 7.28 – 7.19 (m, 5H), 4.12 (ddd, *J* = 10.6, 8.7, 3.8 Hz, 1H), 3.70 (q, *J* = 6.7 Hz, 2H), 3.06 (dd, *J* = 13.8, 3.8 Hz, 1H), 2.78 (dd, *J* = 13.8, 10.6 Hz, 1H), 1.52 – 1.43 (m, 2H), 1.31 (s, 9H), 1.23 (d, *J* = 4.4 Hz, 18H), 0.89 – 0.79 (m, 3H); **^{31}P NMR (122 MHz, DMSO-d$_6$)** δ (ppm) -8.07.

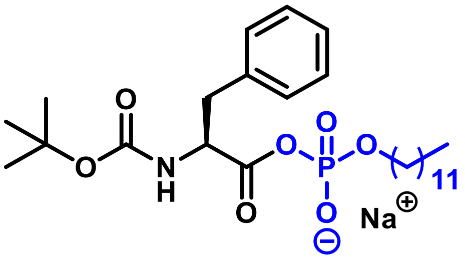

Boc-FDDP

[v] (S)-2-(((benzyloxy)carbonyl)amino)-3-phenylpropanoic (ethyl phosphoric) anhydride: The title compound was prepared according to the general procedure [I, II & III] as white solid in 52% yield. **^{1}H NMR (400 MHz, DMSO-d_6)** δ (ppm) 7.75 – 7.59 (m, 1H), 7.37 – 7.19 (m, 11H), 4.96 (s, 2H), 4.30 – 4.17 (m, 1H), 3.85 – 3.73 (m, 2H), 3.13 (dd, *J* = 14.0, 3.9 Hz, 1H), 2.83 (dd, *J* = 14.2, 11.3 Hz, 1H), 1.12 (t, *J* = 7.3 Hz, 3H); **^{31}P NMR (162 MHz, DMSO-d_6)** δ (ppm) -8.12.[3]

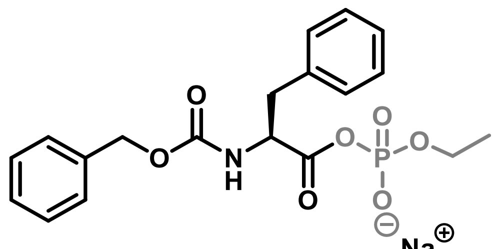

Z-FEP

[vi] (S)-2-(((benzyloxy)carbonyl)amino)-3-phenylpropanoic (phenyl phosphoric) anhydride: The title compound was prepared according to the general procedure [I, II & III] as white solid in 52% yield. **^{1}H NMR (300 MHz, DMSO-d_6)** δ (ppm) 7.68 (d, *J* = 8.8 Hz, 1H), 7.37 – 7.01 (m, 15H), 4.95 (s, 2H), 4.20 (ddd, *J* = 11.0, 8.7, 3.6 Hz, 1H), 3.01 (dd, *J* = 13.9, 3.7 Hz, 1H), 2.73 (dd, *J* = 13.9, 11.1 Hz, 1H); **^{31}P NMR (162 MHz Chloroform-*d*)** δ (ppm) -12.42.

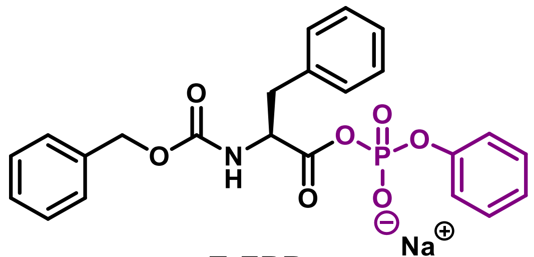

Z-FPP

[vii] (S)-2-(((benzyloxy)carbonyl)amino)-3-phenylpropanoic (naphthyl phosphoric) anhydride: The title compound was prepared according to the general procedure [I, II & III] as white solid in 52% yield. **^{1}H NMR (300 MHz, DMSO-d$_6$)** δ (ppm) 7.88 – 7.55 (m, 5H), 7.48 – 7.13 (m, 13H), 4.93 (s, 2H), 4.22 (ddd, *J* = 11.0, 8.8, 3.7 Hz, 1H), 3.02 (dd, *J* = 13.8, 3.6 Hz, 1H), 2.74 (dd, *J* = 13.9, 11.0 Hz, 1H); **^{31}P NMR (162 MHz Chloroform-*d*)** δ (ppm) -12.42.

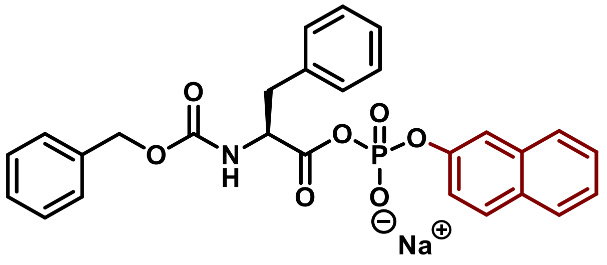

Z-FNP

[viii] (S)-2-(((benzyloxy)carbonyl)amino)-3-phenylpropanoic (dodecyl phosphoric) anhydride: The title compound was prepared according to the general procedure [I, II & III] as white hygroscopic solid in 60% yield. **^{1}H NMR (300 MHz, DMSO-d$_6$)** δ (ppm) 7.62 (d, *J* = 8.8 Hz, 1H), 7.34 – 7.20 (m, 10H), 4.95 (s, 2H), 4.21 (ddd, *J* = 10.9, 8.7, 3.7 Hz, 1H), 3.70 (q, *J* = 6.7 Hz, 2H), 3.12 (dd, *J* = 13.8, 3.8 Hz, 1H), 2.85 – 2.76 (m, 1H), 1.46 (d, *J* = 7.8 Hz, 2H), 1.22 (d, *J* = 1.2 Hz, 19H), 0.84 (s, 3H); **^{31}P NMR (122 MHz, DMSO-d$_6$)** δ (ppm) -8.04.

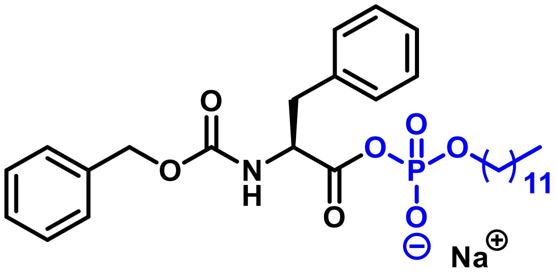

Z-FDDP

[ix] (S)-2-((((9H-fluoren-9-yl)methoxy)carbonyl)amino)-3-phenylpropanoic (ethyl phosphoric) anhydride:

The title compound was prepared according to the general procedure [I, II & III] as white hygroscopic solid in 60% yield. **^{1}H NMR (300 MHz, DMSO-d$_6$)** *δ* (ppm) 7.89 – 7.84 (m, 2H), 7.62 (dd, *J* = 7.6, 4.4 Hz, 2H), 7.40 (tt, *J* = 7.6, 1.5 Hz, 2H), 7.34 – 7.27 (m, 2H), 7.26 – 7.21 (m, 5H), 7.20 (q, *J* = 2.5 Hz, 2H), 7.16 – 7.10 (m, 2H), 6.97 (ddt, *J* = 7.2, 6.0, 1.1 Hz, 1H), 4.24 – 4.17 (m, 1H), 4.17 – 4.01 (m, 3H), 3.01 (dd, *J* = 13.8, 3.7 Hz, 1H), 2.77 (dd, *J* = 13.8, 11.0 Hz, 1H); **^{31}P NMR (162 MHz, Chloroform-*d*)** *δ* (ppm) -6.20.

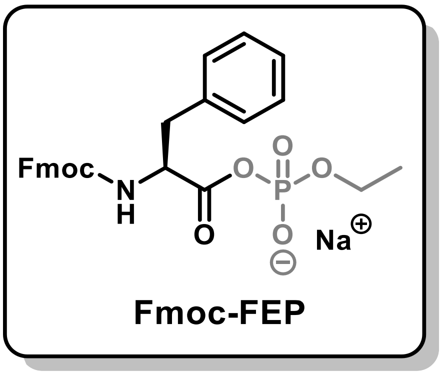

[x] (S)-2-((((9H-fluoren-9-yl)methoxy)carbonyl)amino)-3-phenylpropanoic (phenyl phosphoric) anhydride:

The title compound was prepared according to the general procedure [I, II & III] as white hygroscopic solid in 60% yield. **^{1}H NMR (300 MHz, DMSO-d$_6$)** *δ* (ppm) 7.87 (d, *J* = 7.5 Hz, 2H), 7.69 – 7.58 (m, 2H), 7.41 (tdd, *J* = 7.5, 2.2, 1.2 Hz, 2H), 7.35 – 7.26 (m, 5H), 7.26 – 7.14 (m, 2H), 4.27 – 4.18 (m, 1H), 4.16 (s, 3H), 3.78 (dq, *J* = 8.2, 7.2 Hz, 2H), 3.13 (dd, *J* = 13.9, 3.8 Hz, 1H), 2.84 (dd, *J* = 13.8, 11.0 Hz, 1H), 1.15 – 1.04 (m, 3H); **^{31}P NMR (162 MHz, Chloroform-*d*)** *δ* (ppm) -13.82.

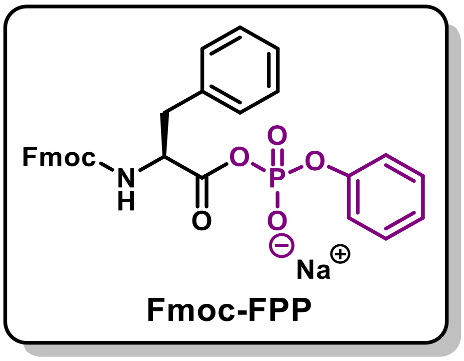

[xi] (S)-2-amino-3-phenylpropanoic (dodecyl phosphoric) anhydride: The title compound was prepared according to the general procedure [I, II & IV] as white hygroscopic solid in 42% yield.**^{1}H NMR (300 MHz, D_2O)** *δ* (ppm) 7.46 – 7.33 (m, 5H), 4.00 (dd, *J* = 7.9, 5.3 Hz, 1H), 3.87 (q, *J* = 6.7 Hz, 2H), 3.31 (dd, *J* = 14.5, 5.3 Hz, 1H), 3.14 (dd, *J* = 14.5, 7.9 Hz, 1H), 1.65 (t, *J* = 7.0 Hz, 2H), 1.28 (s, 18H), 0.93 – 0.82 (m, 3H), **^{31}P NMR (122 MHz, D_2O)** *δ* (ppm) -8.11.

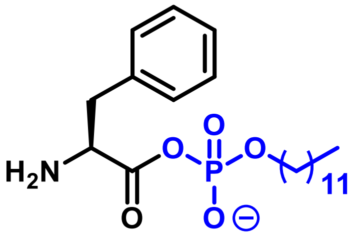

FDDP

[xii] tert-butyl ((S)-1-(((S)-1-amino-4-methyl-1-oxopentan-2-yl)amino)-1-oxo-3-phenylpropan-2-yl)carbamate: The title compound was prepared according to the earlier reported method[5] as a white hygroscopic solid in 48% yield.**^{1}H NMR (500 MHz, DMSO-d_6)** *δ* (ppm) *δ* 7.56 (d, *J* = 8.4 Hz, 1H), 7.01 (d, *J* = 5.4 Hz, 5H), 6.94 – 6.89 (m, 1H), 6.72 (d, *J* = 8.6 Hz, 2H), 4.01 (q, *J* = 8.1 Hz, 1H), 3.91 (s, 1H), 2.72 (dd, *J* = 13.8, 4.7 Hz, 1H), 2.53 – 2.46 (m, 1H), 1.24 – 1.17 (m, 2H), 1.06 (s, 8H), 1.03 – 0.97 (m, 2H), 0.67 – 0.57 (m, 6H). **^{13}C NMR (126 MHz, DMSO-d_6)** *δ* (ppm) 173.9, 171.2, 155.2, 138.1, 129.1, 127.9, 126.1, 78.0, 55.8, 50.7, 41.2, 33.3, 28.0, 25.3, 23.1, 21.5.

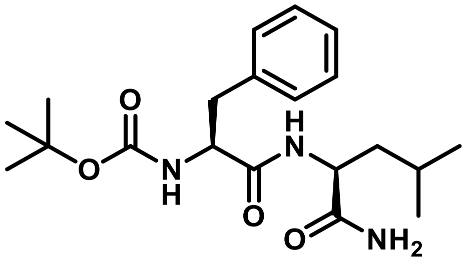

Boc-FL-NH$_2$

EP-Salt ^{1}H, 300 MHz, D_2O

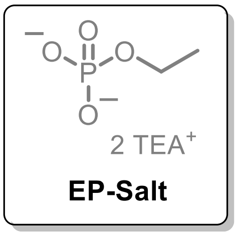

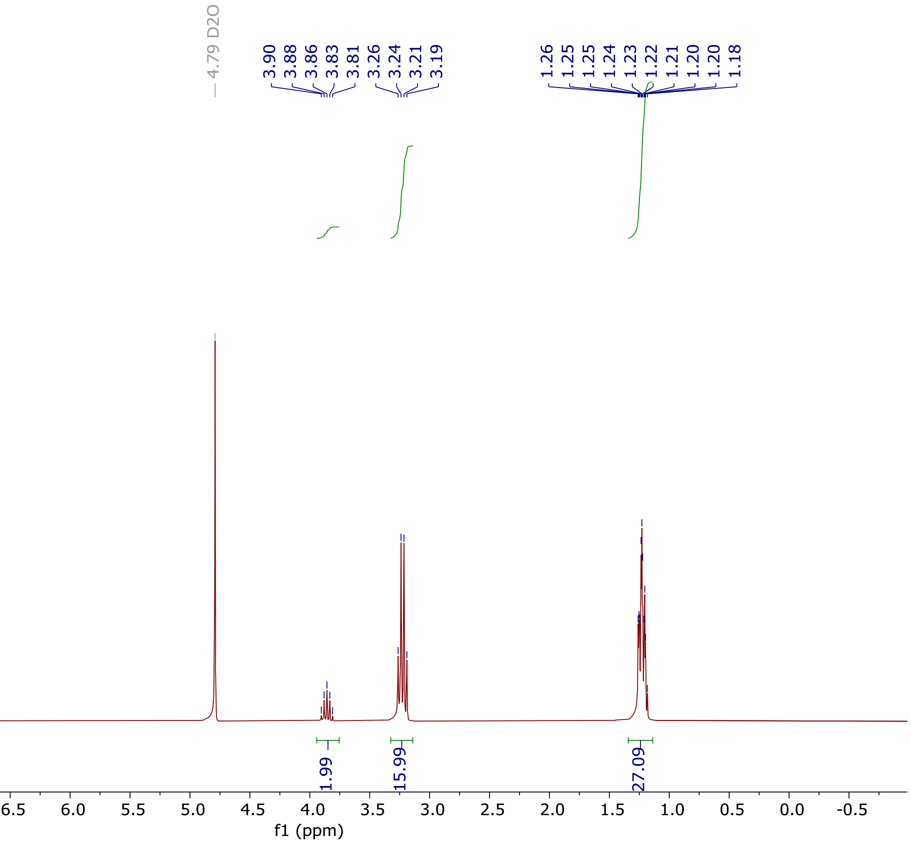

EP-Salt ^{31}P, 122 MHz, D_2O

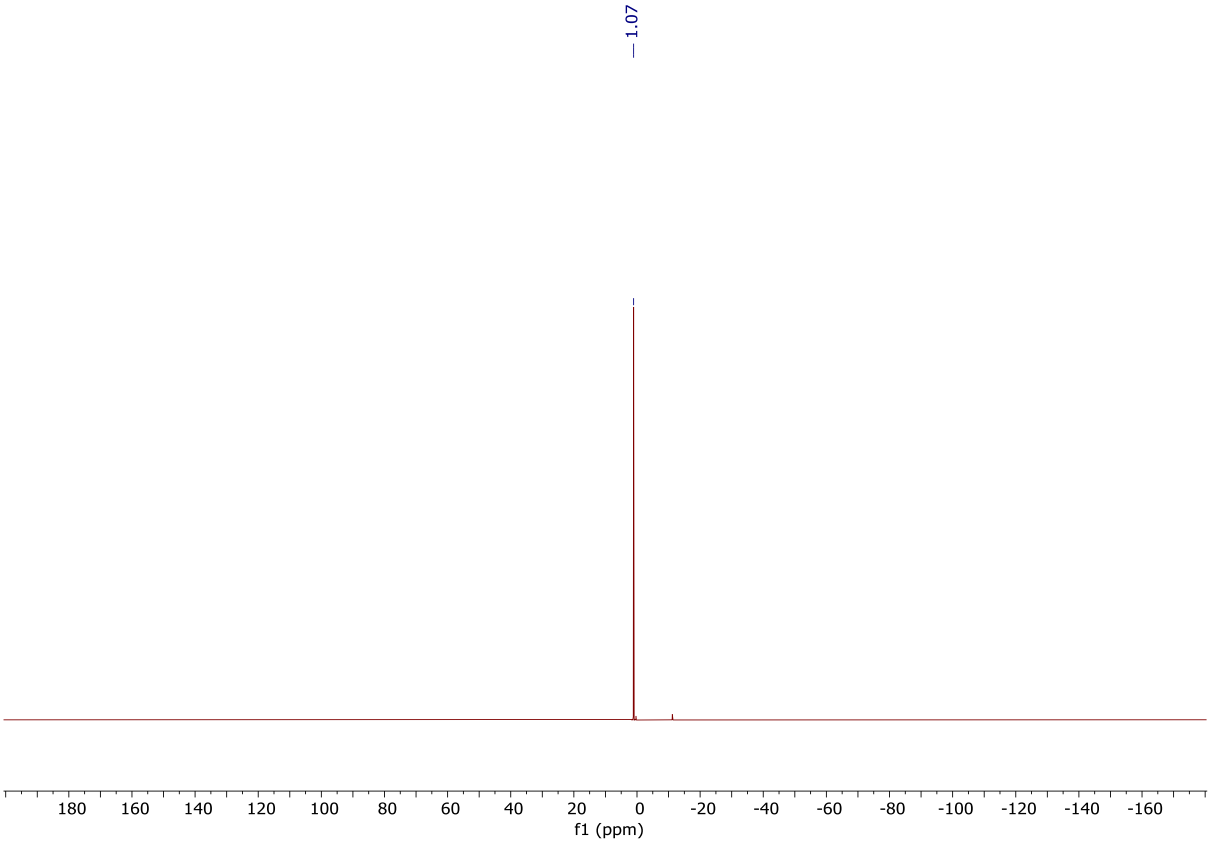

PP-Salt ^{1}H, 300 MHz, D_2O

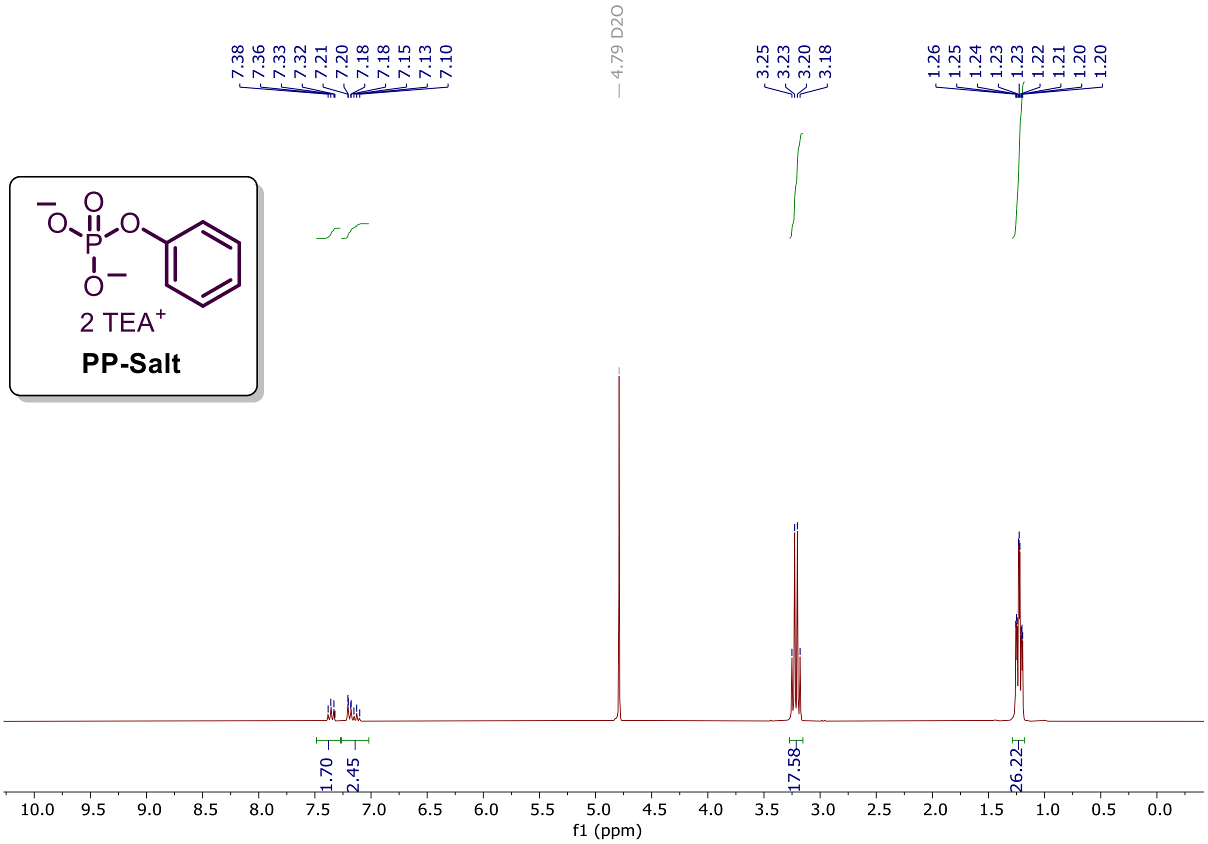

PP-Salt ^{31}P, 122 MHz, D_2O

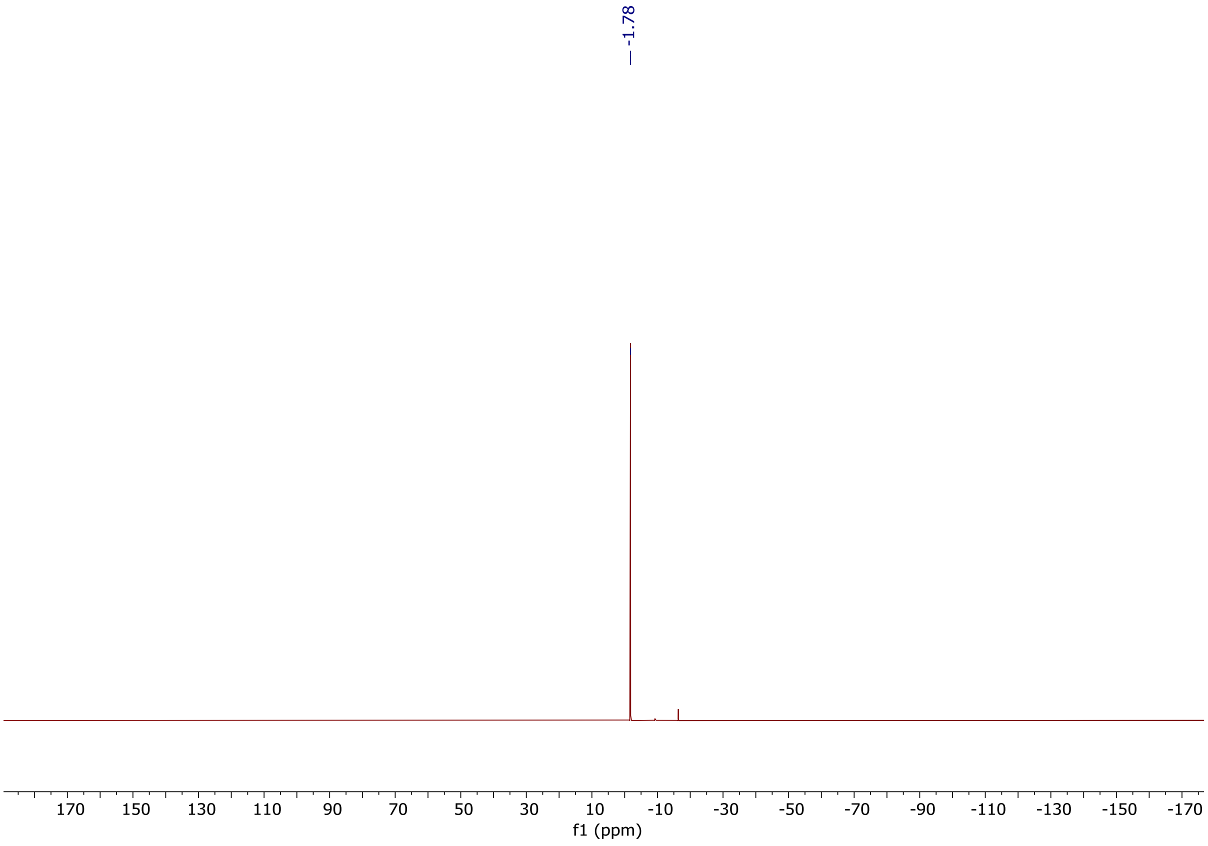

NP-Salt ^{1}H, 300 MHz, D_2O

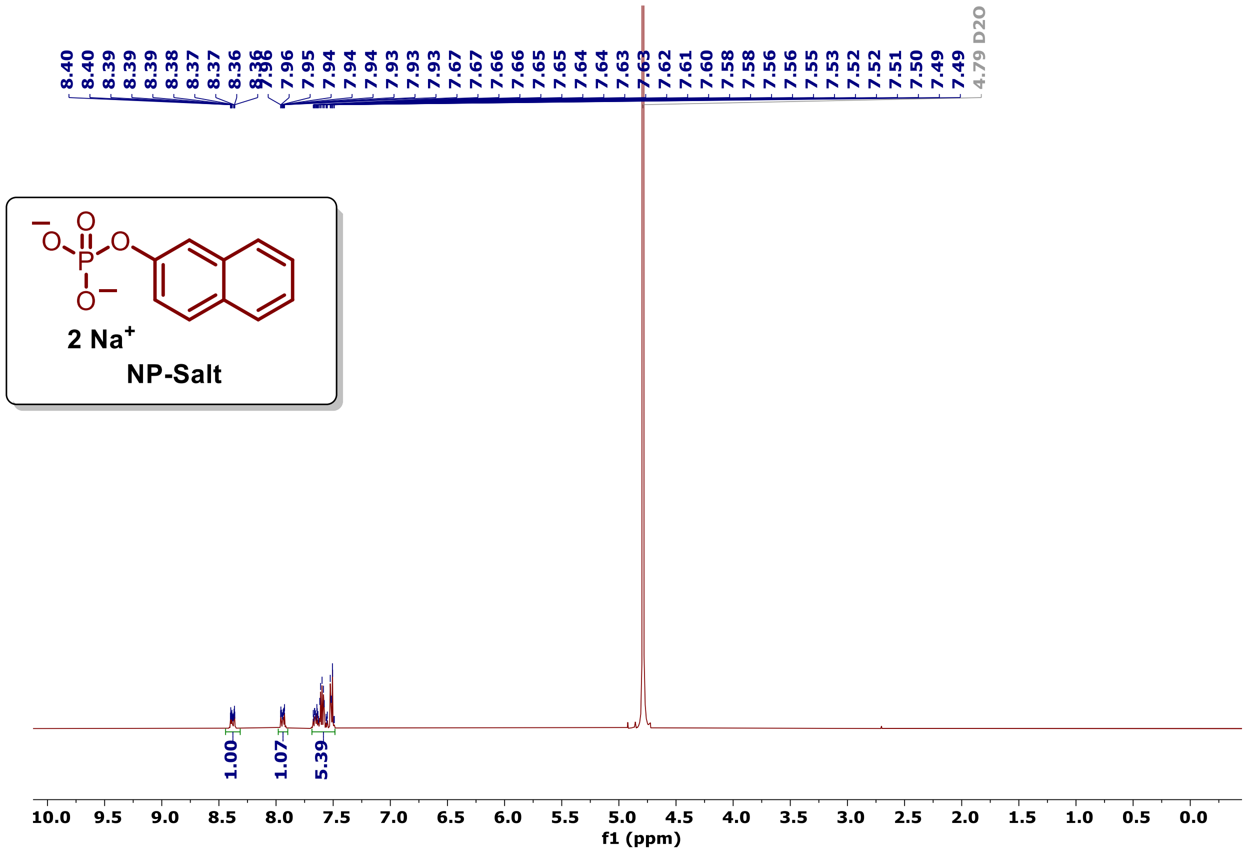

NP-Salt ^{31}P, 122 MHz, D_2O

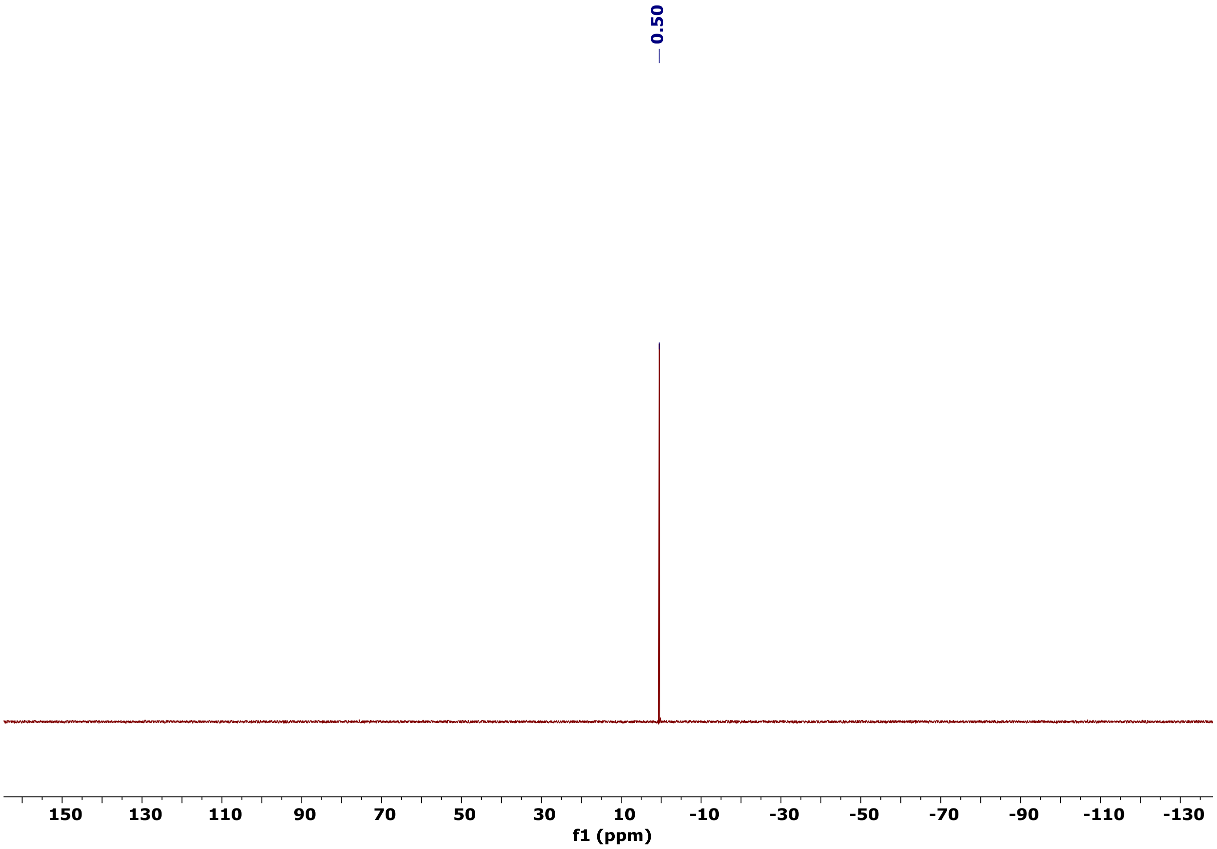

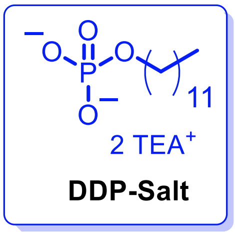

DPP ^{1}H, 300 MHz, $CDCl_3$

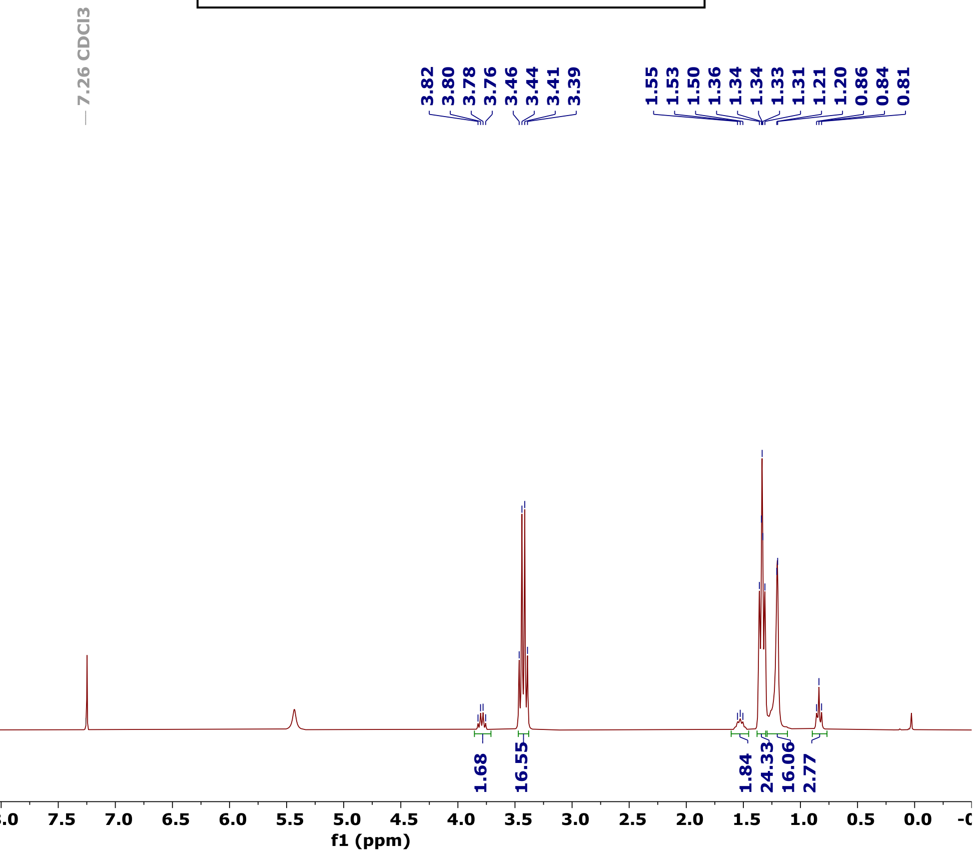

DPP ^{31}P, 122 MHz, $CDCl_3$

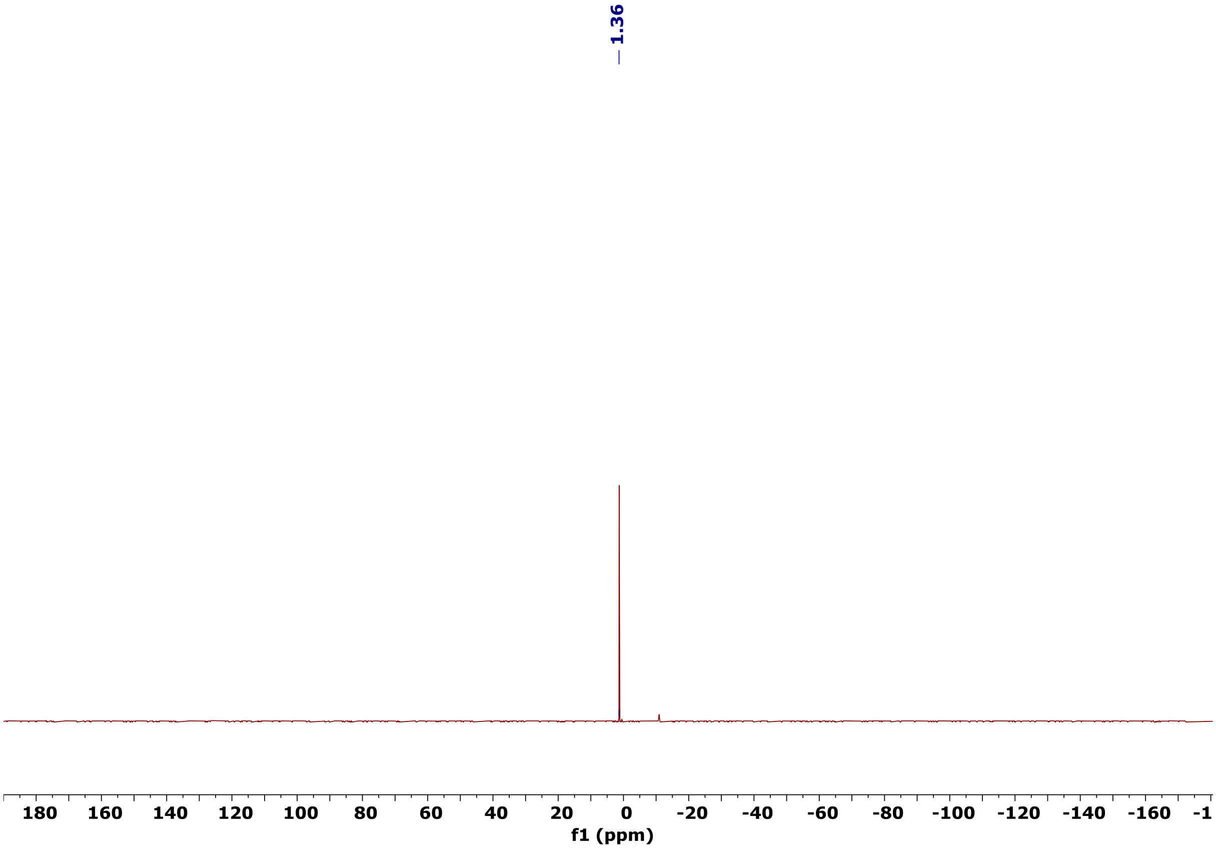

Boc-FEP ^{1}H, 300 MHz, $CDCl_3$

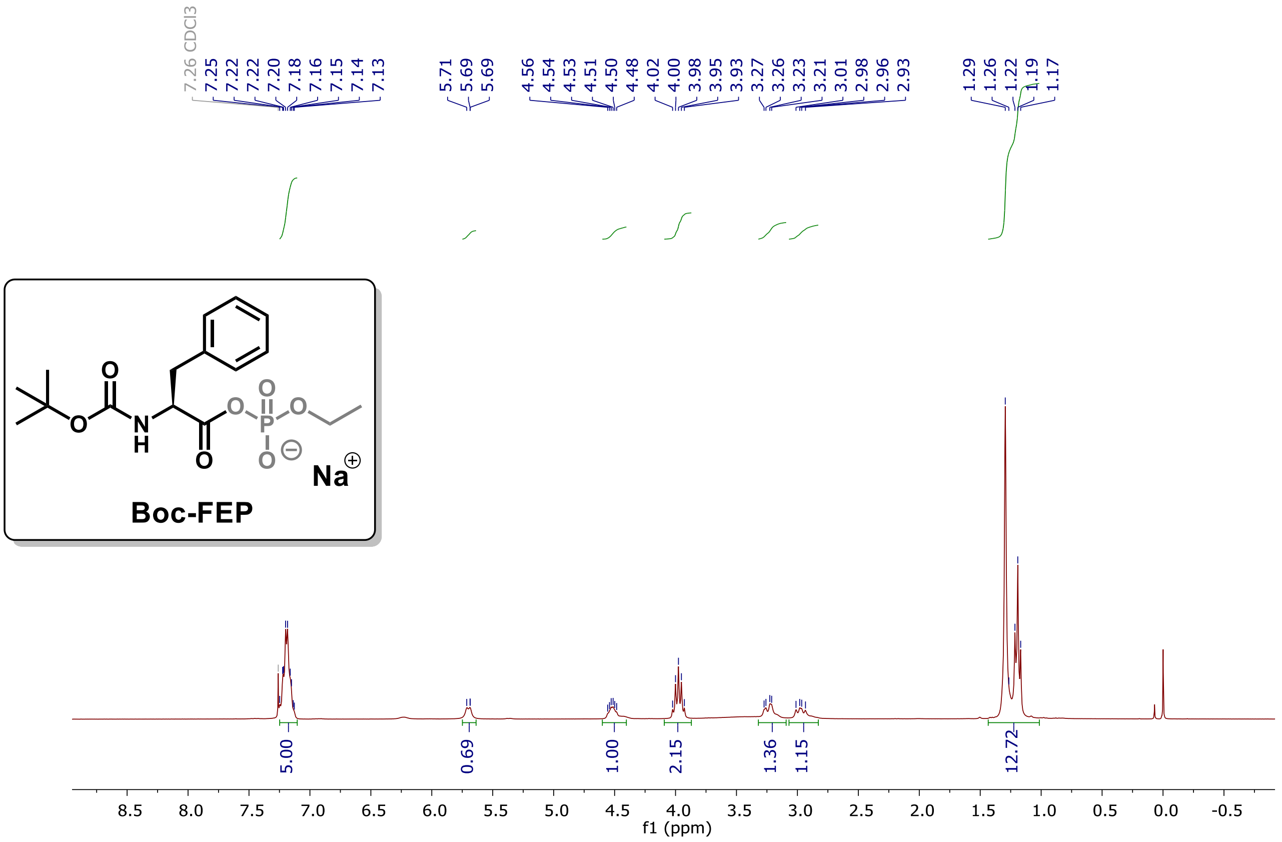

Boc-FEP ^{31}P, 122 MHz, $CDCl_3$

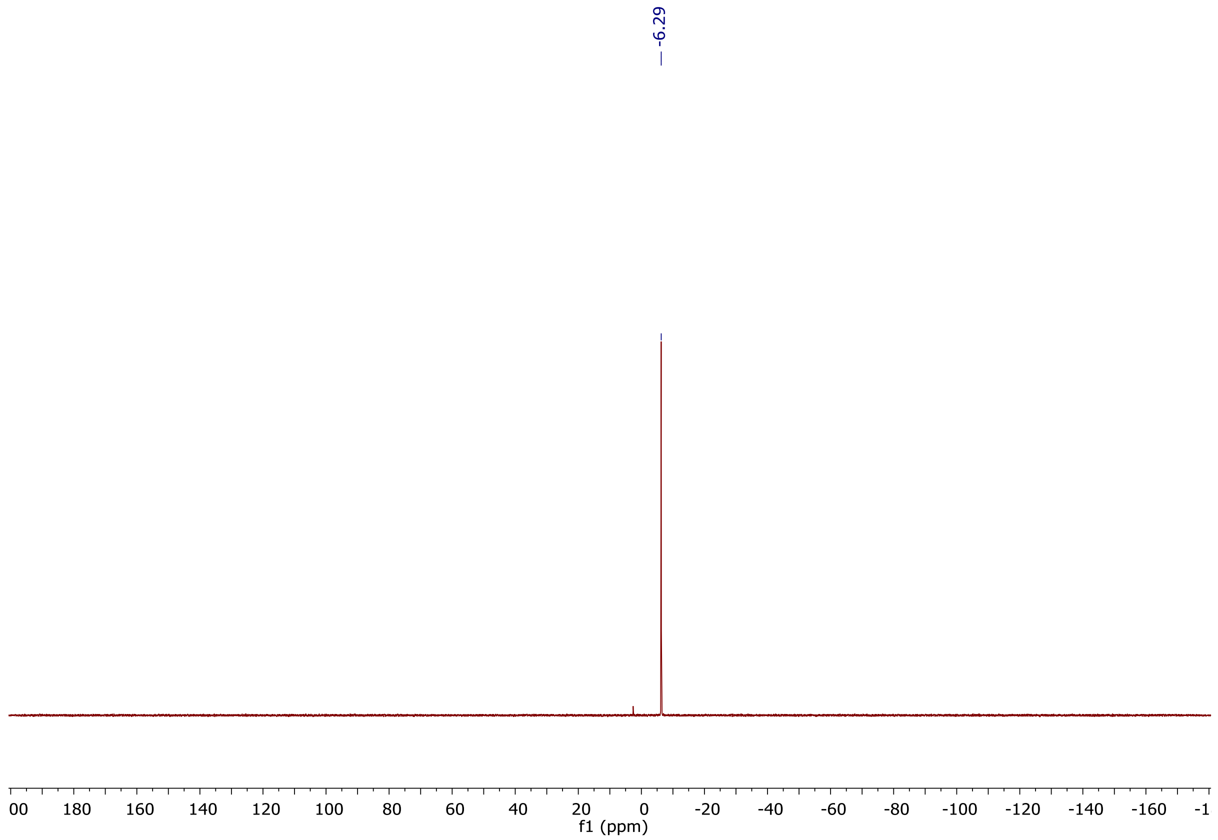

Boc-FPP ^1H, 300 MHz, DMSO-d$_6$

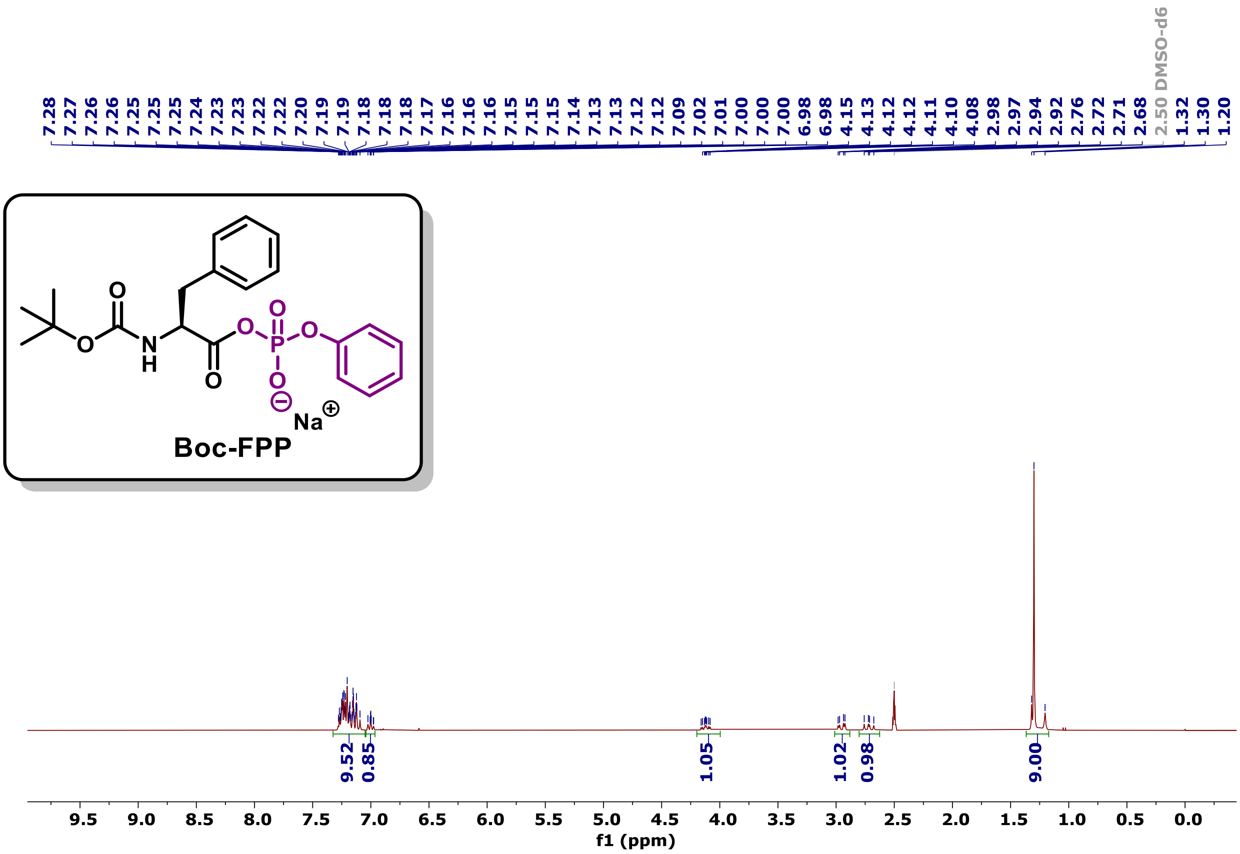

Boc-FPP ^{31}P, 162 MHz, $CDCl_3$

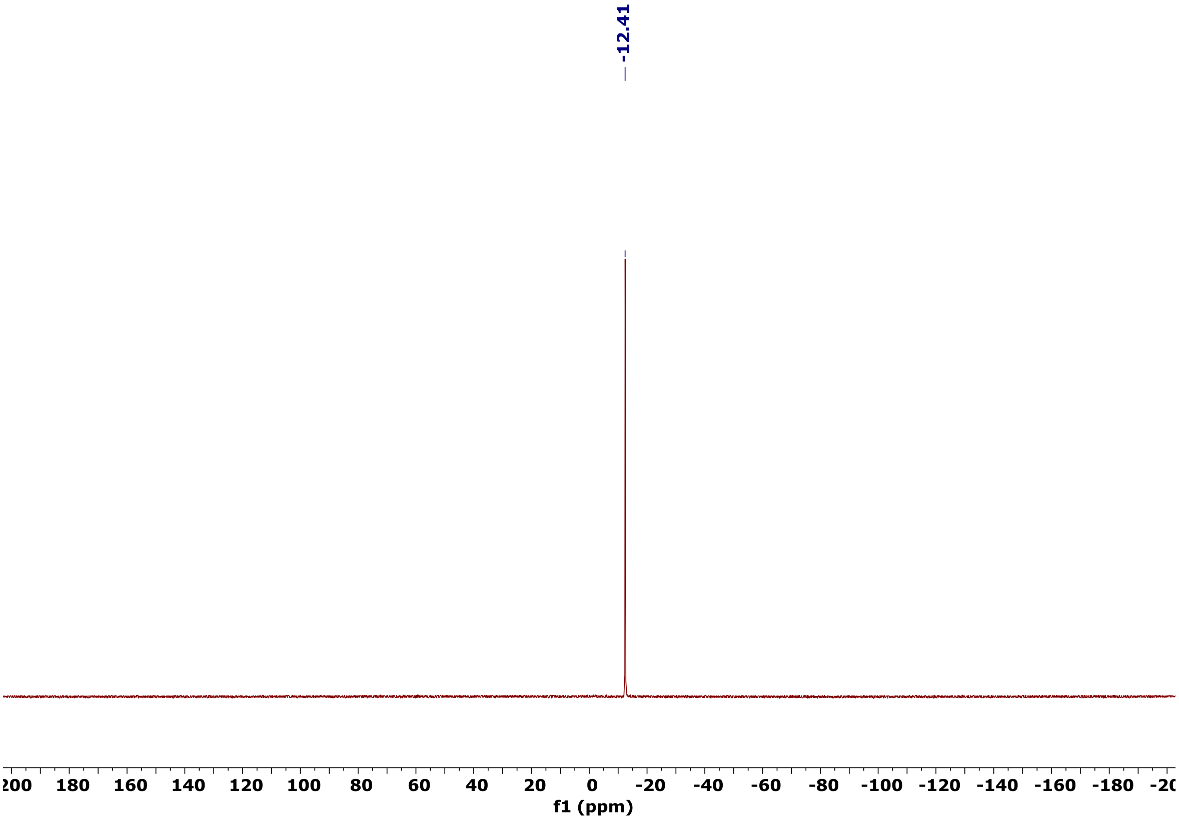

Boc-FNP ^{1}H, 300 MHz, DMSO-d_6

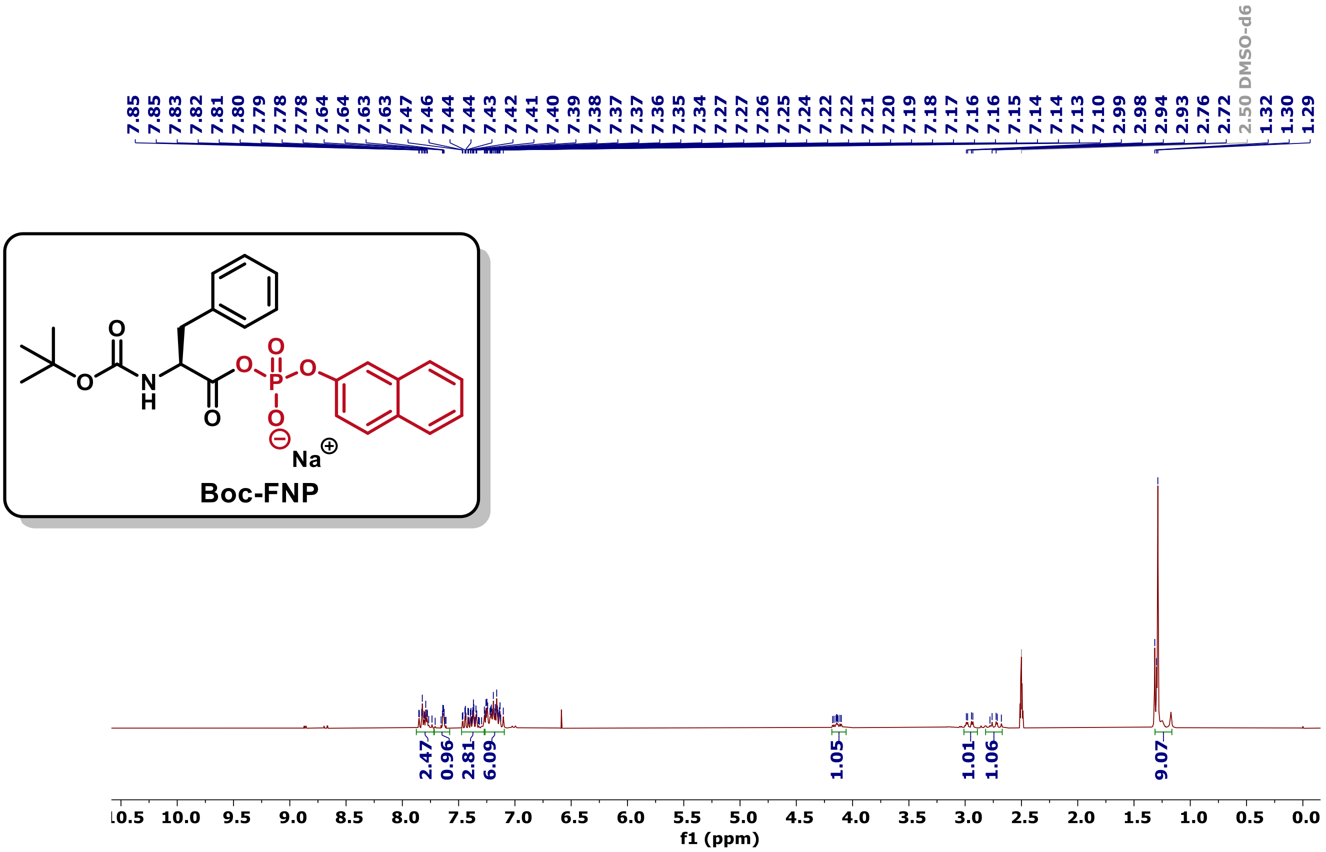

Boc-FNP ^{31}P, 162 MHz, $CDCl_3$

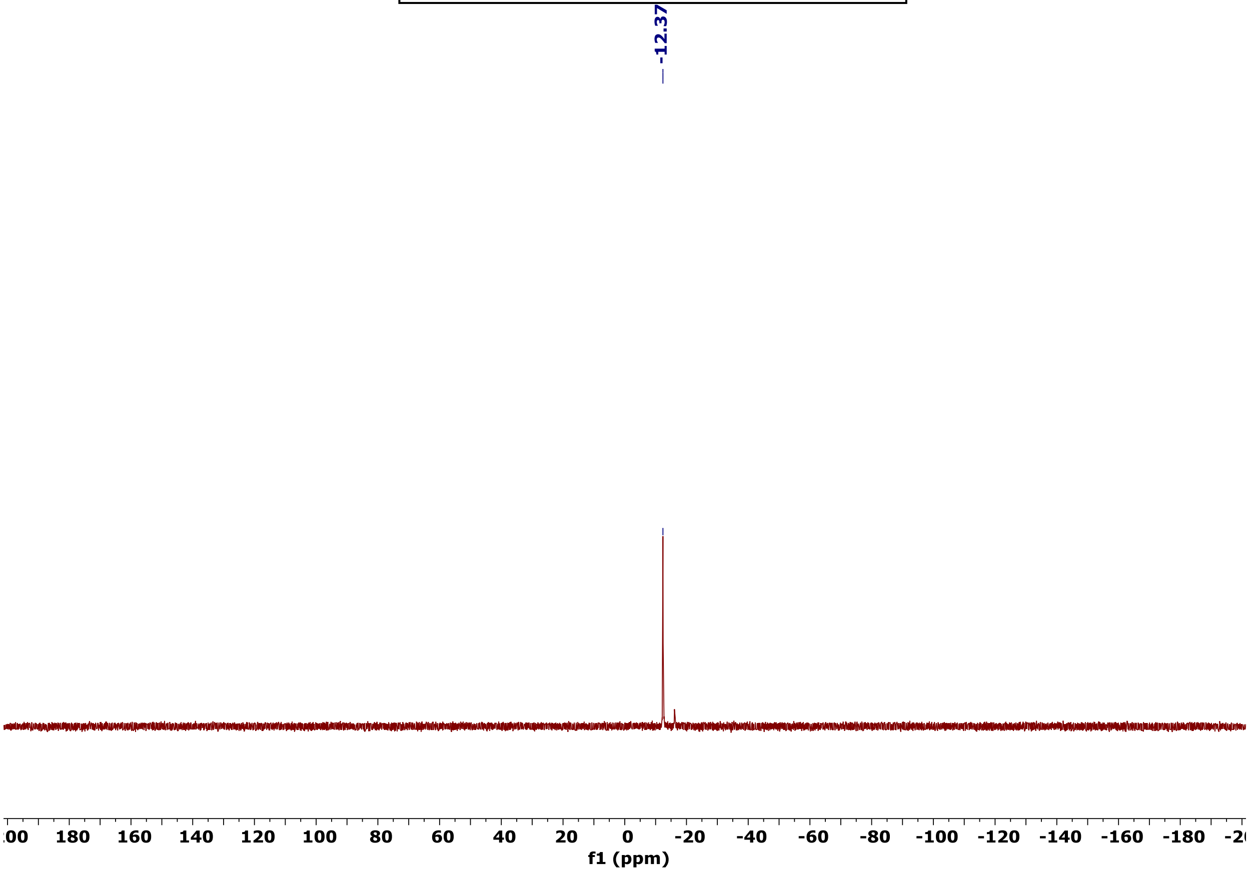

Boc-FDDP ^{1}H, 300 MHz, DMSO-d_6

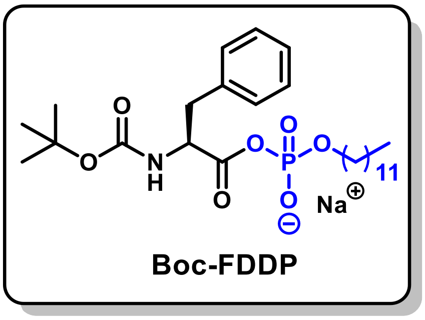

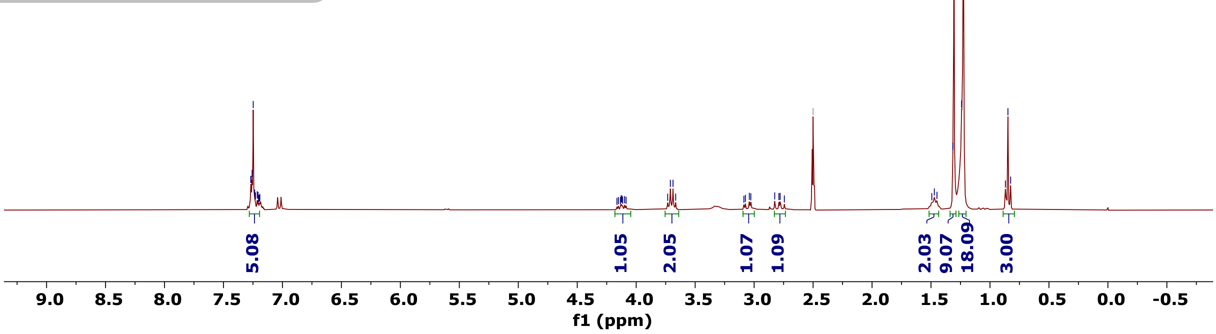

Boc-FDDP ^{31}P, 122 MHz, DMSO-d_6

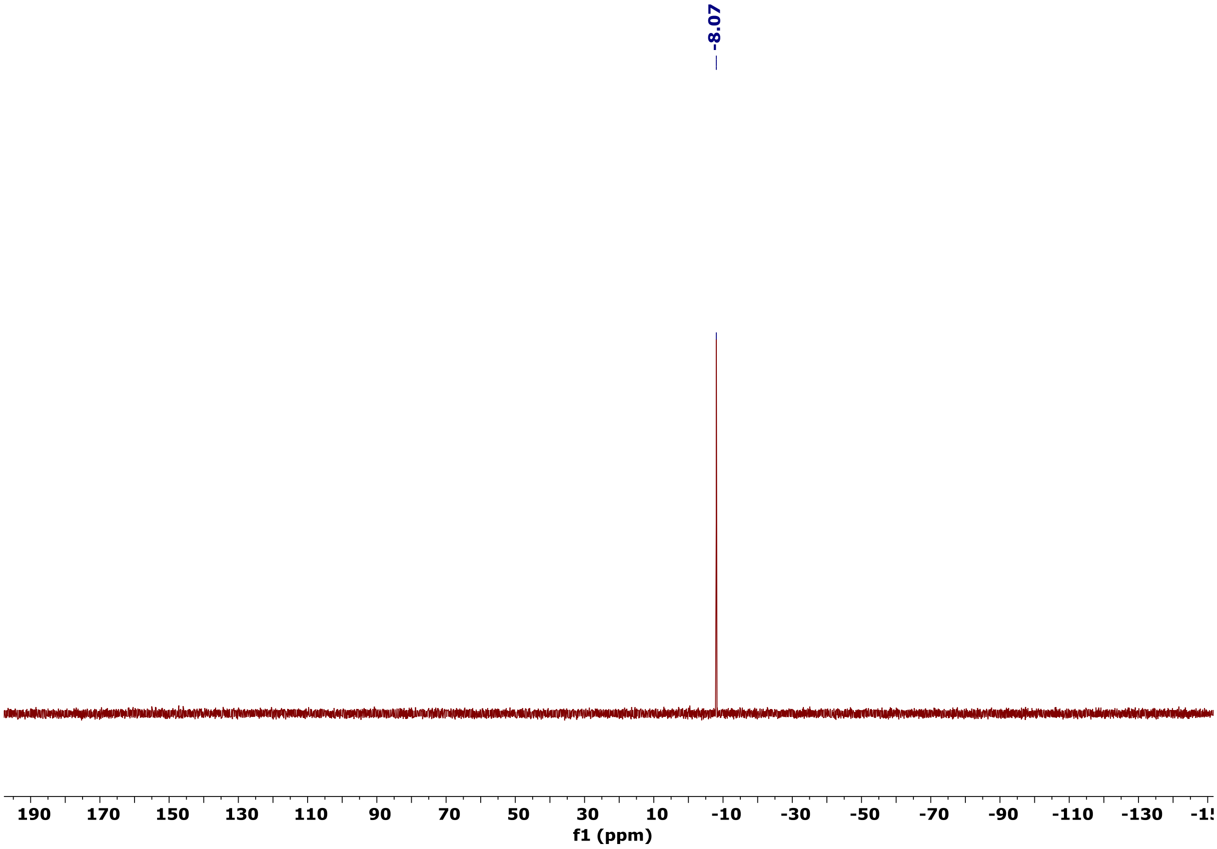

Z-FEP ^{1}H, 400 MHz, DMSO-d_6

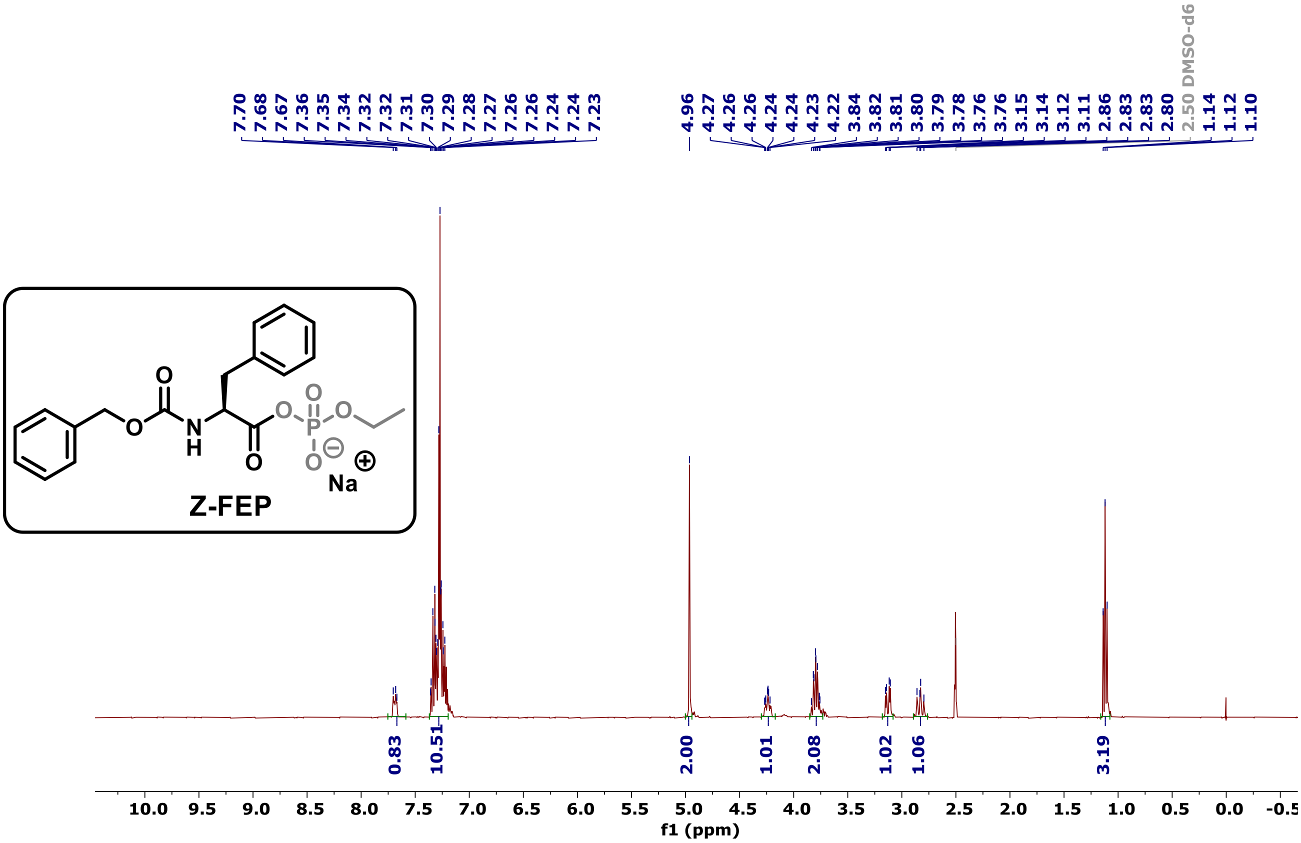

Z-FEP ^{31P}, 162 MHz, DMSO-d_6

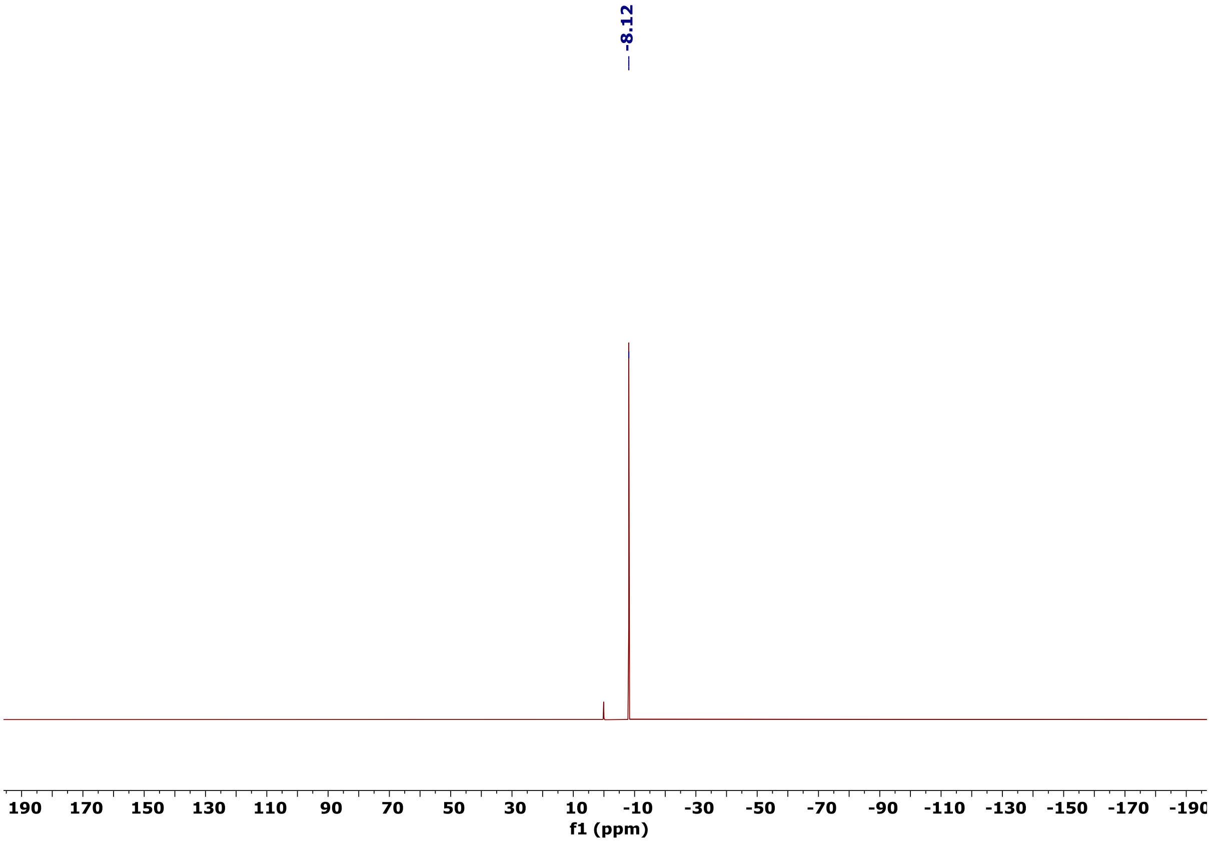

Z-FPP ^{1}H, 300 MHz, DMSO-d_6

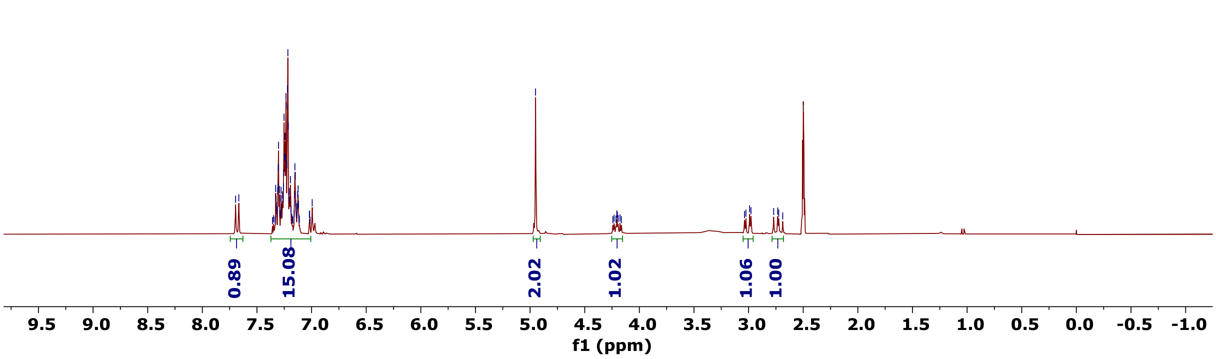

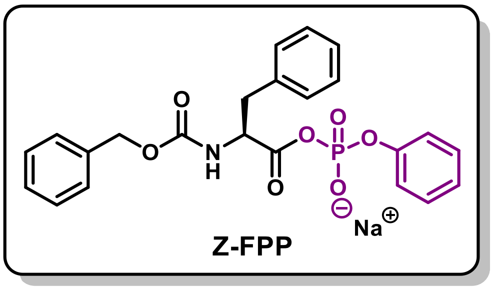

Z-FPP ^{31}P, 162 MHz, $CDCl_3$

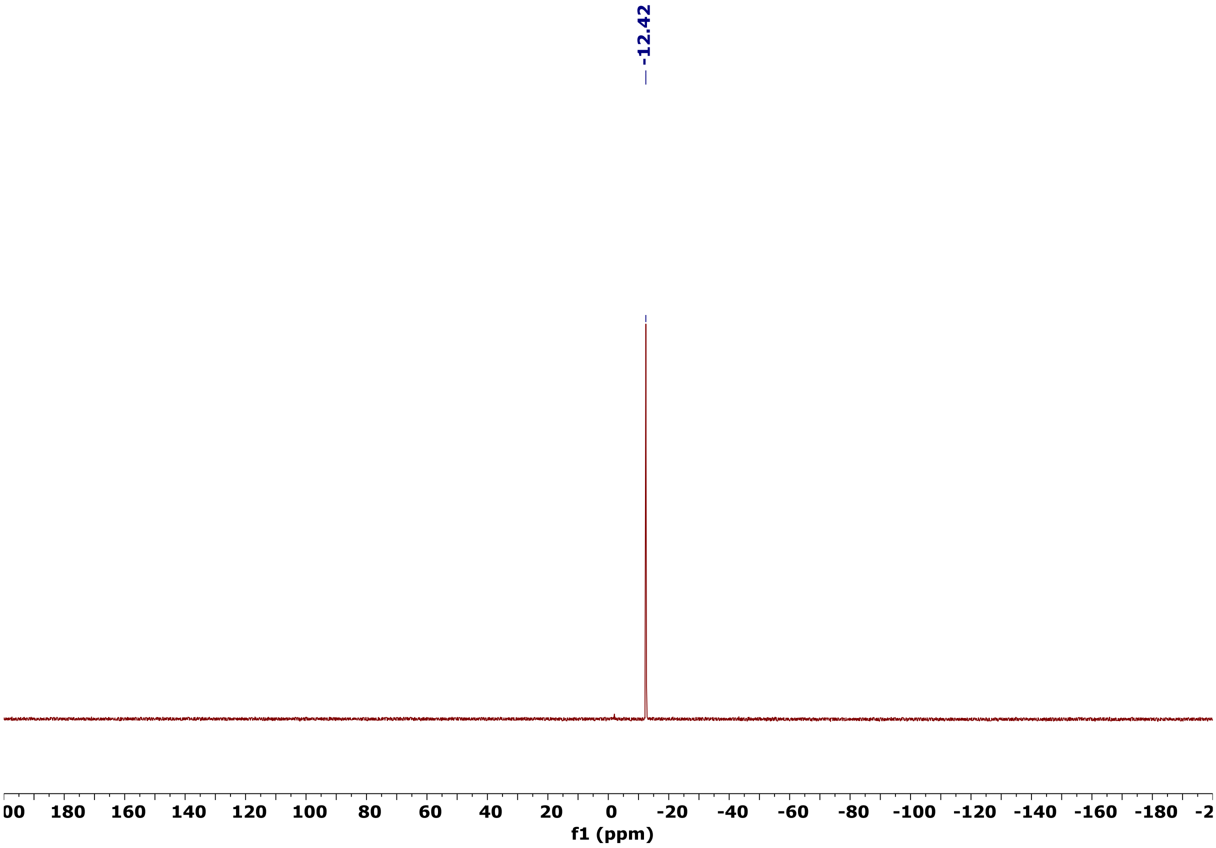

Z-FNP ^{1}H, 300 MHz, DMSO-d_6

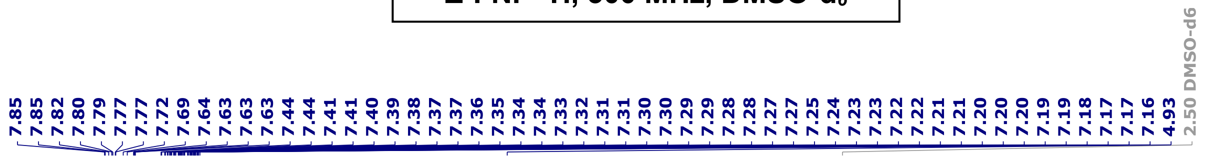

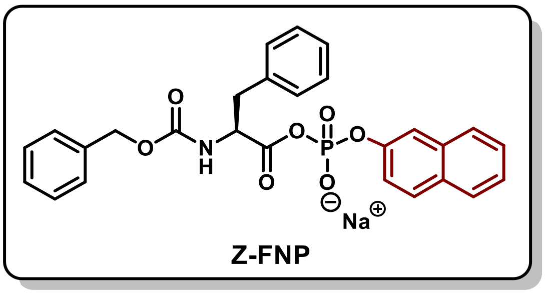

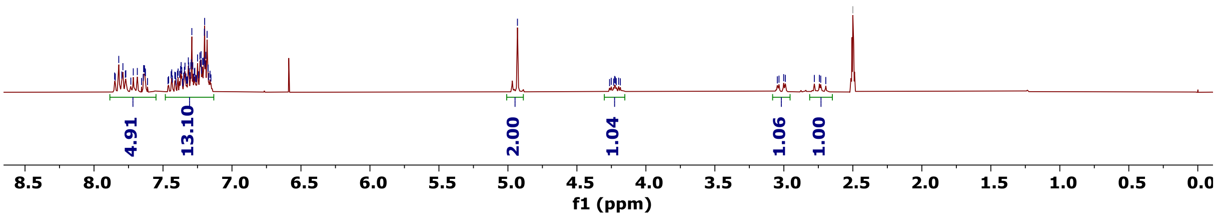

Z-FNP ^{31}P, 162 MHz, $CDCl_3$

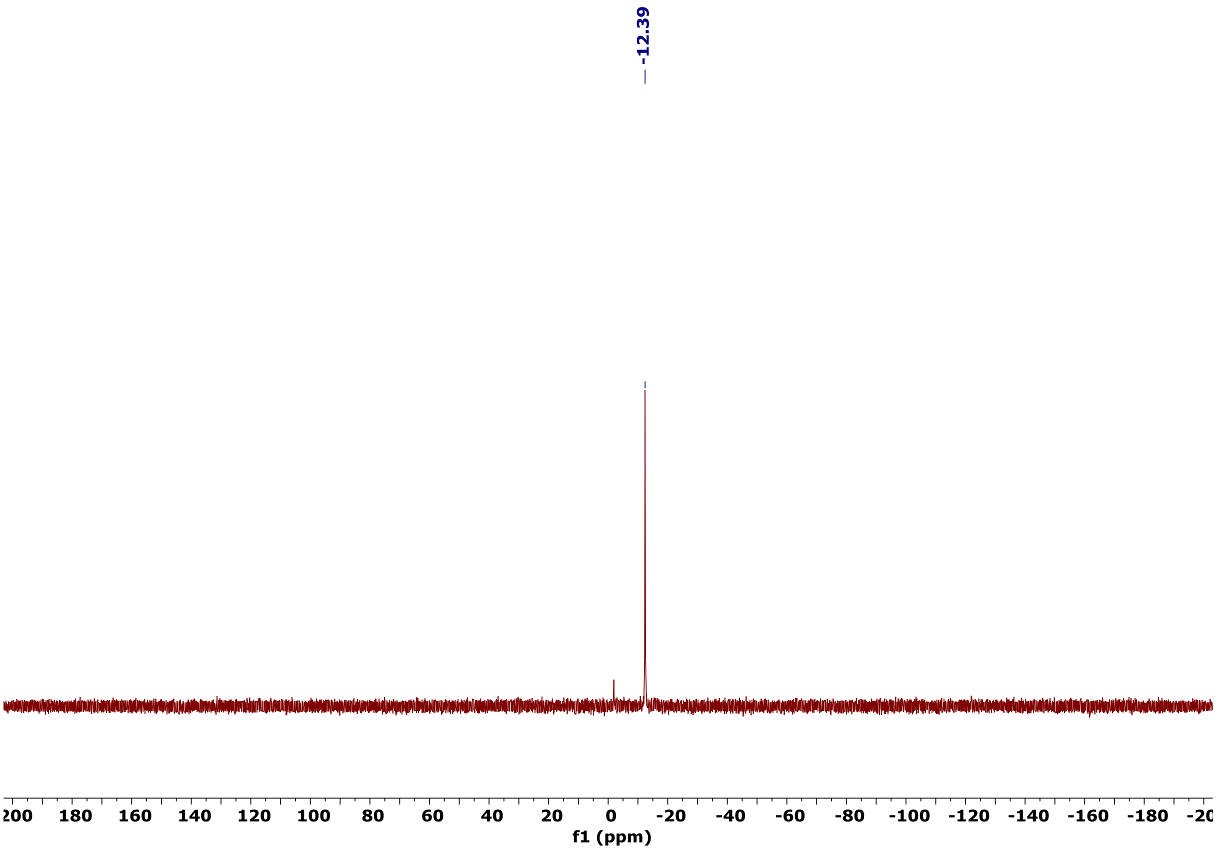

Z-FDDP ^{1}H, 300 MHz, DMSO-d_6

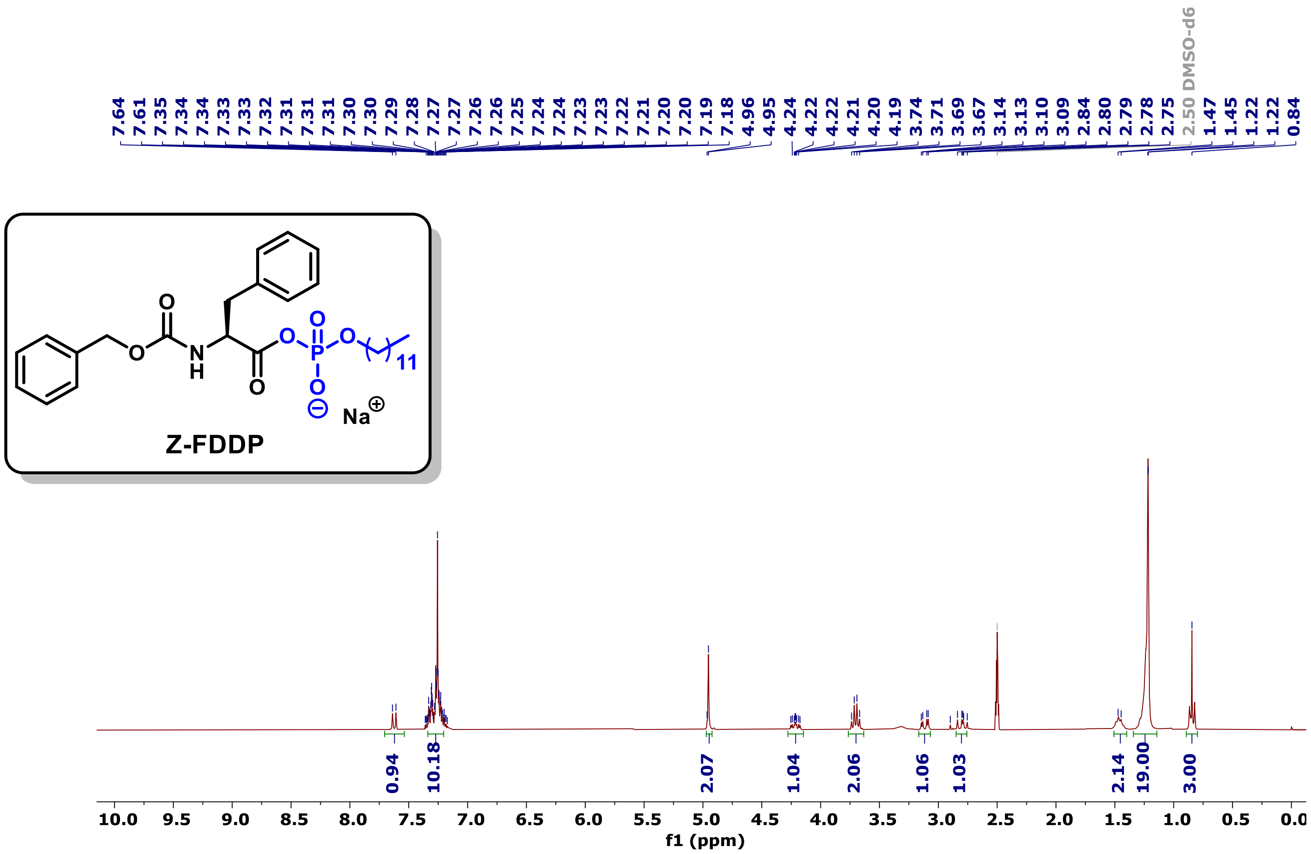

Z-FDDP ^{31}P, 122 MHz, DMSO-d_6

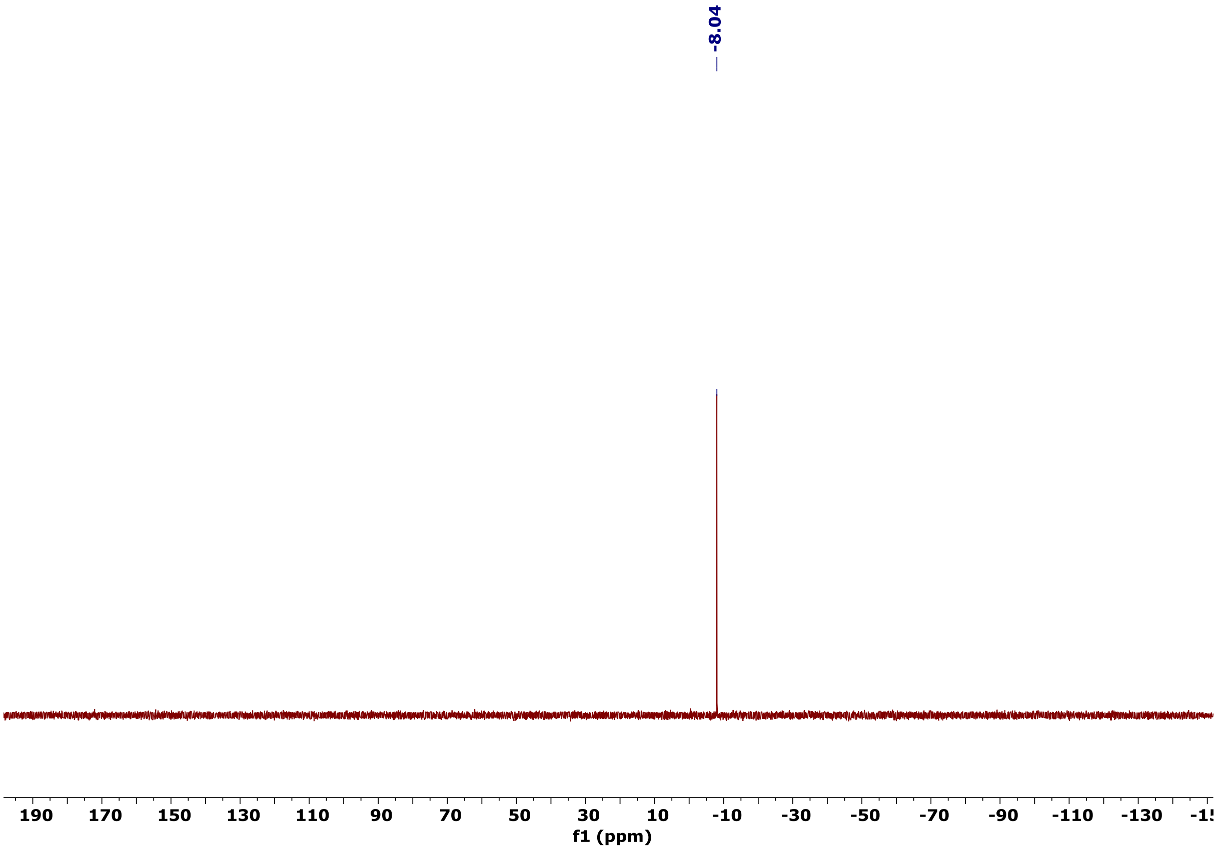

Fmoc-FEP ^{1}H, 300 MHz, DMSO-d_6

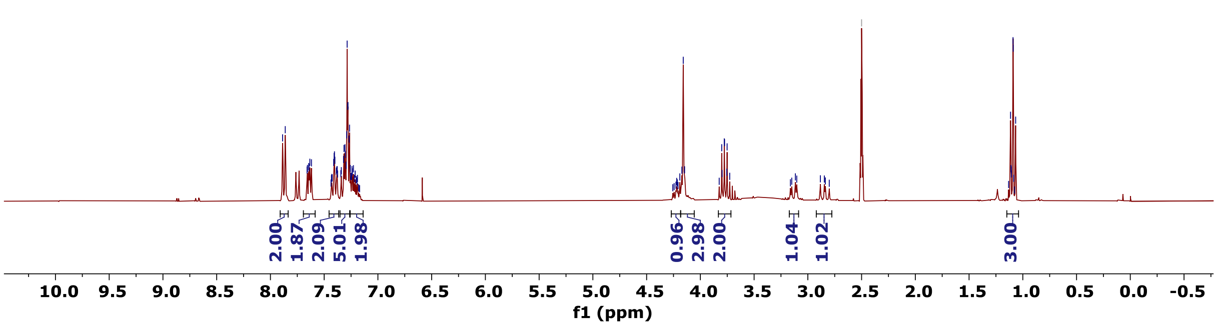

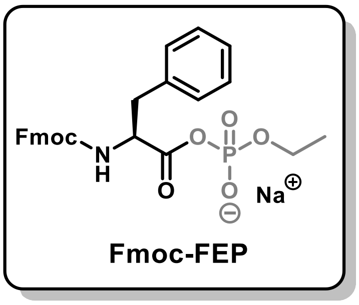

Fmoc-FEP ^{31}P, 162 MHz, $CDCl_3$

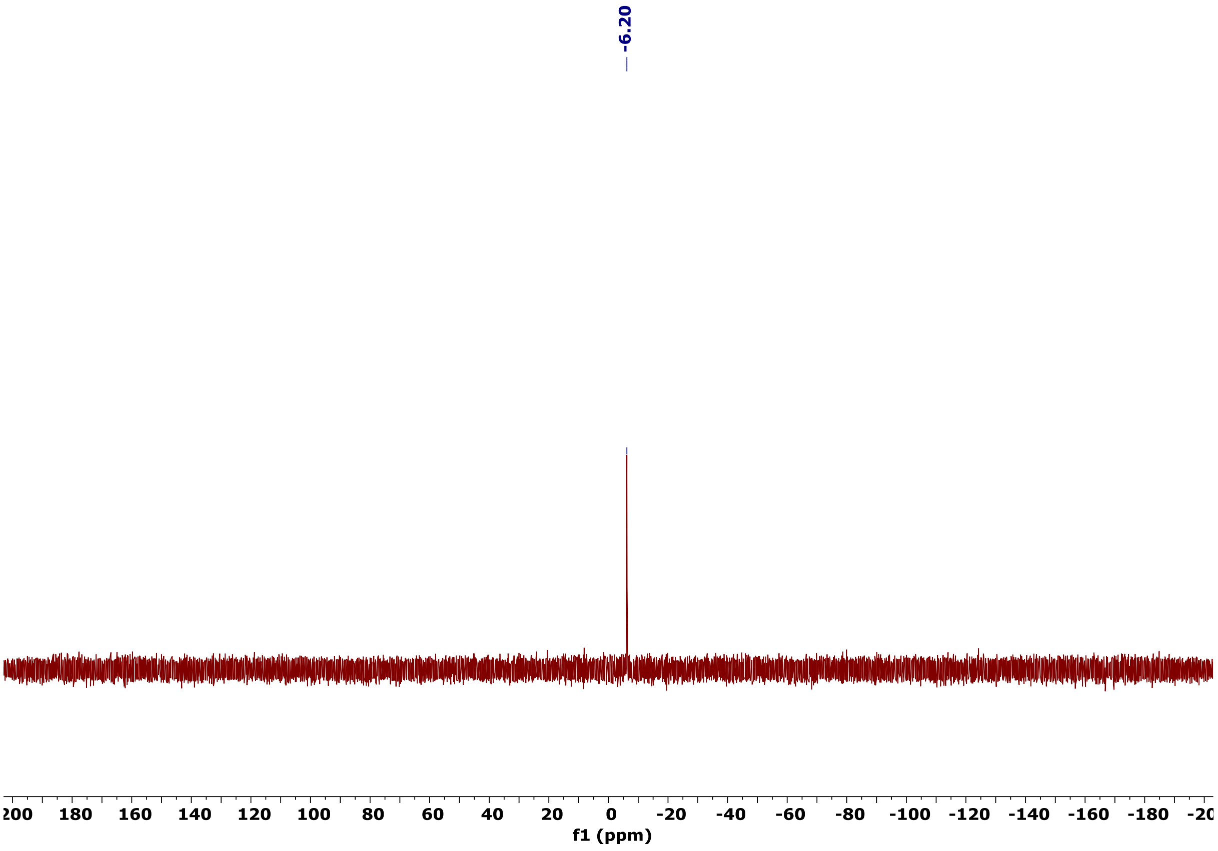

Fmoc-FPP ^{1}H, 300 MHz, DMSO-d$_6$

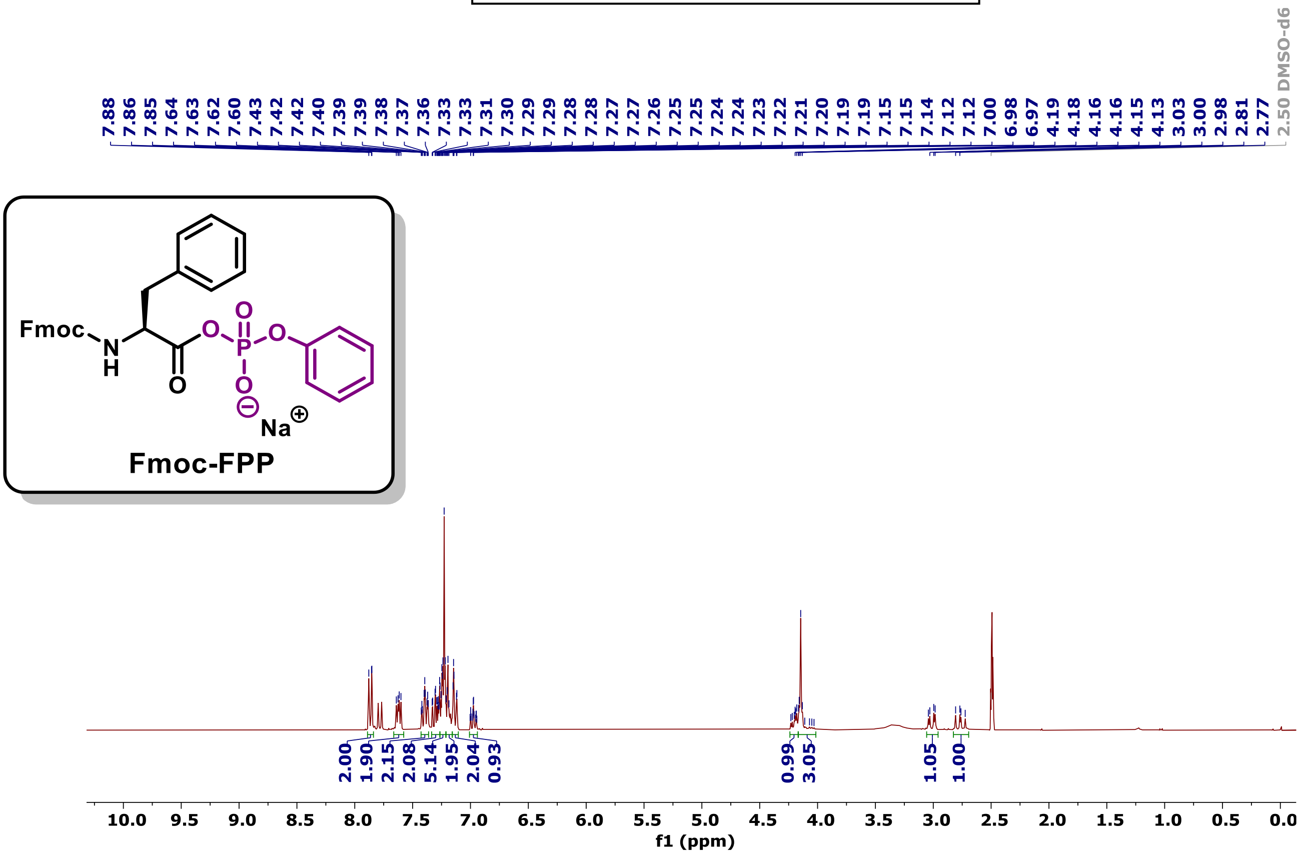

Fmoc-FPP ^{31}P, 162 MHz, CDCl$_3$

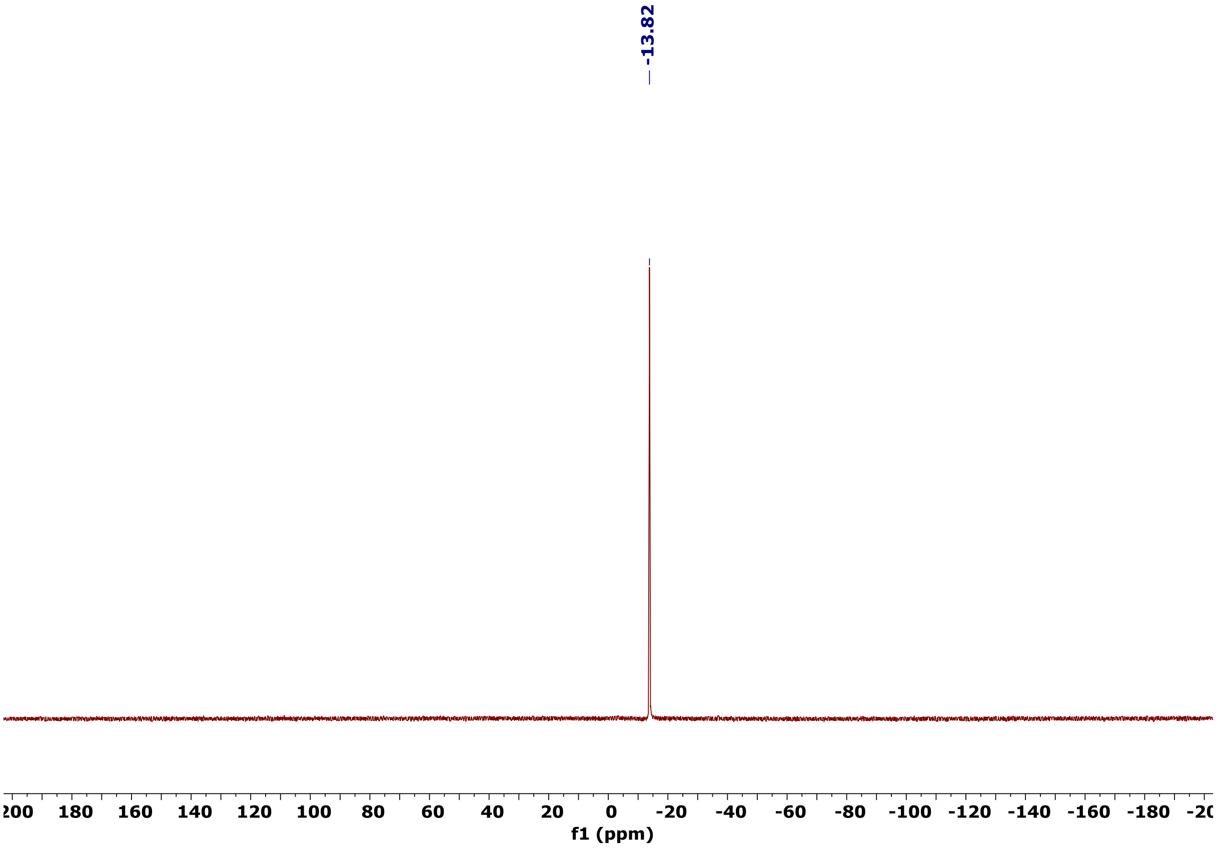

FDDP ^{1}H, 300 MHz, D_2O

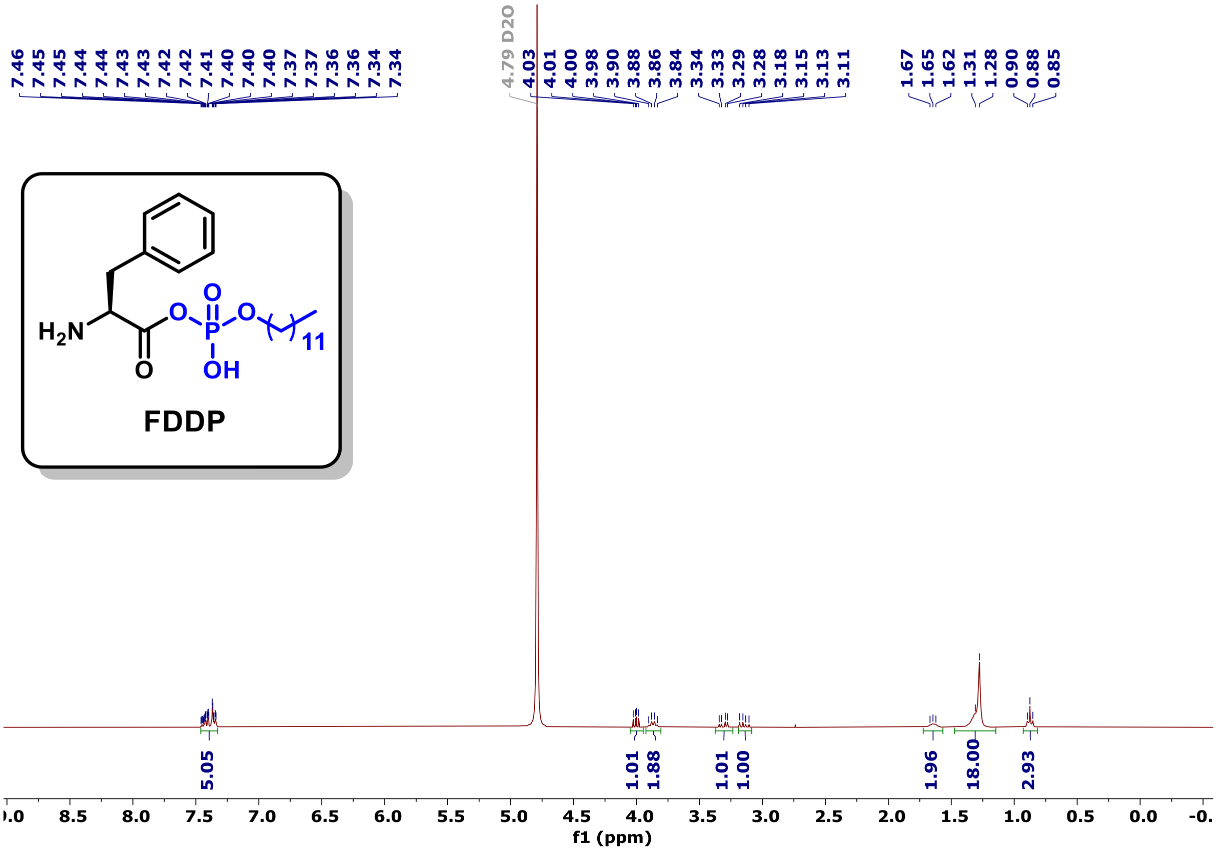

FDDP ^{31}P, 122 MHz, D_2O

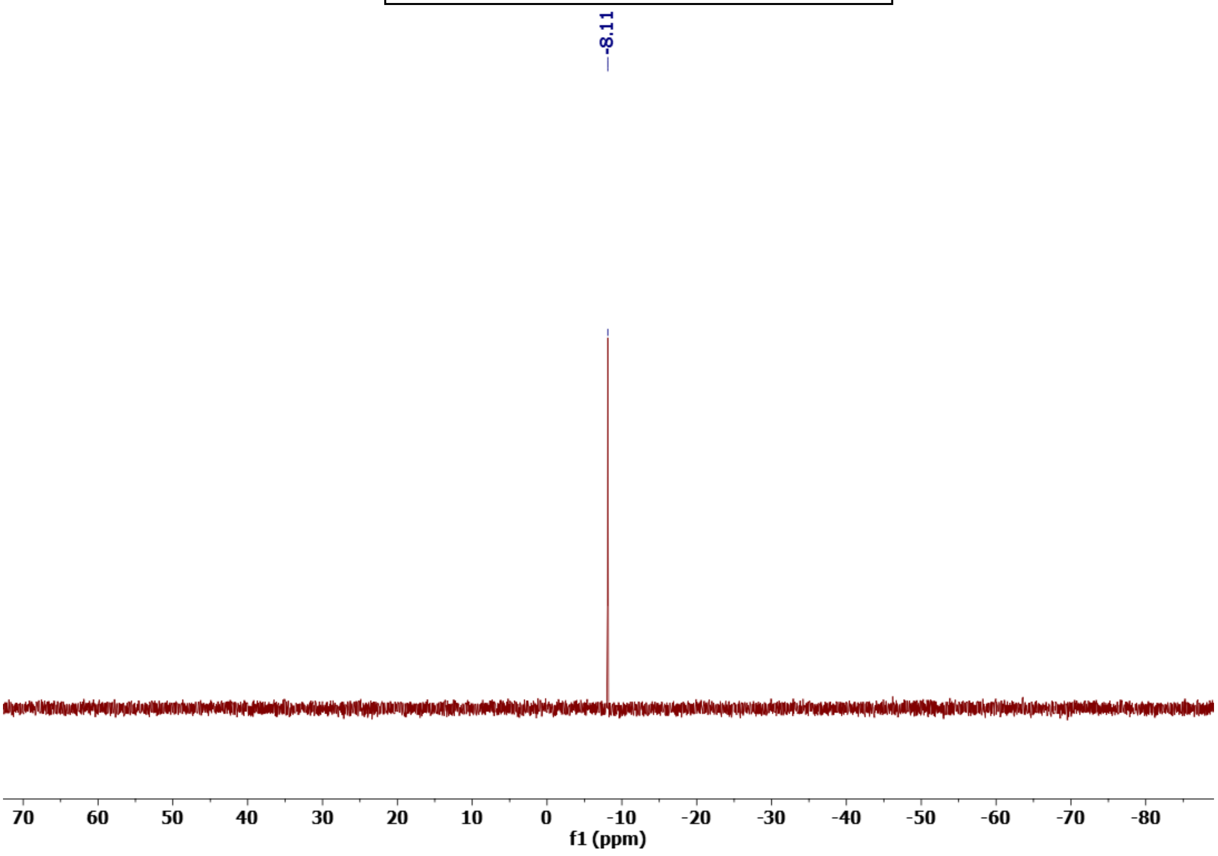

Boc-FL-NH$_2$ ^{1}H, 500 MHz, DMSO-d$_6$

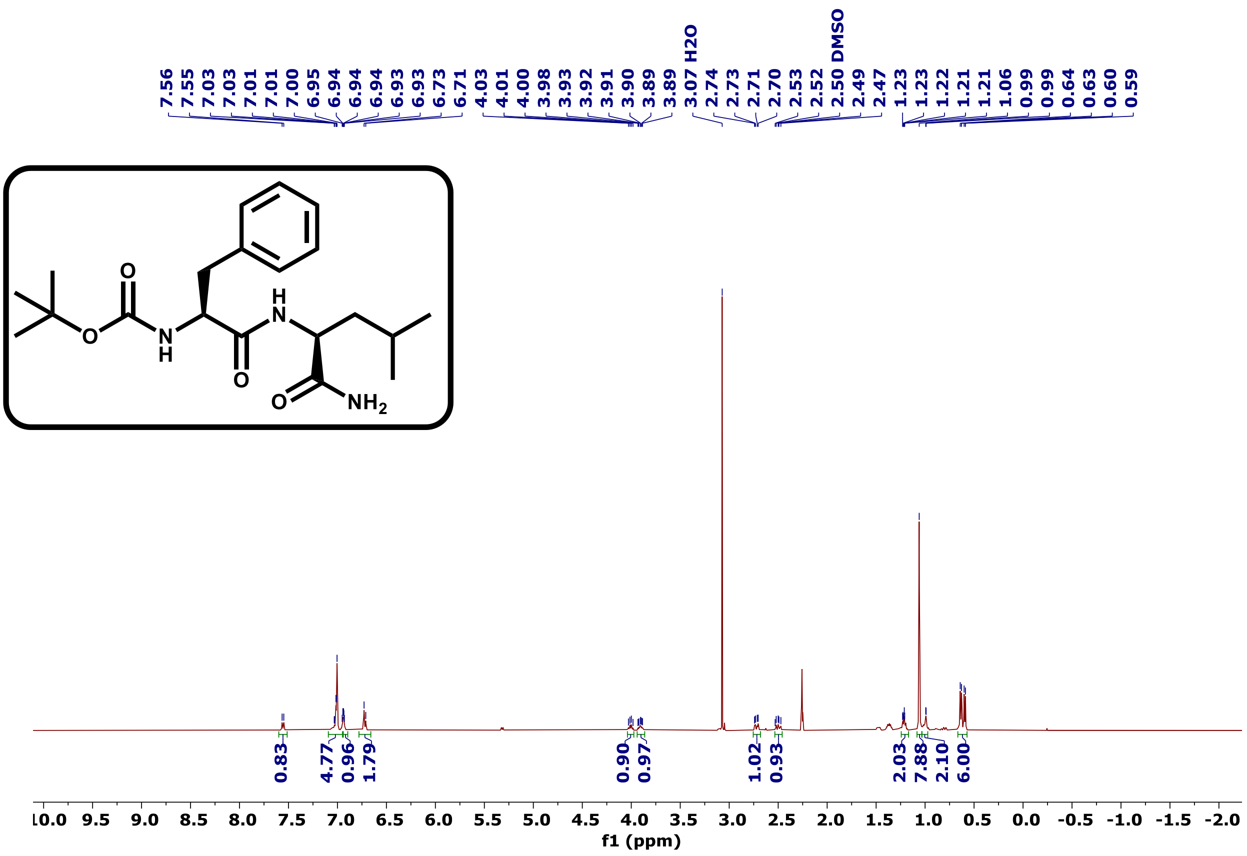

Boc-FL-NH$_2$ ^{13}C, 126 MHz, DMSO-d$_6$

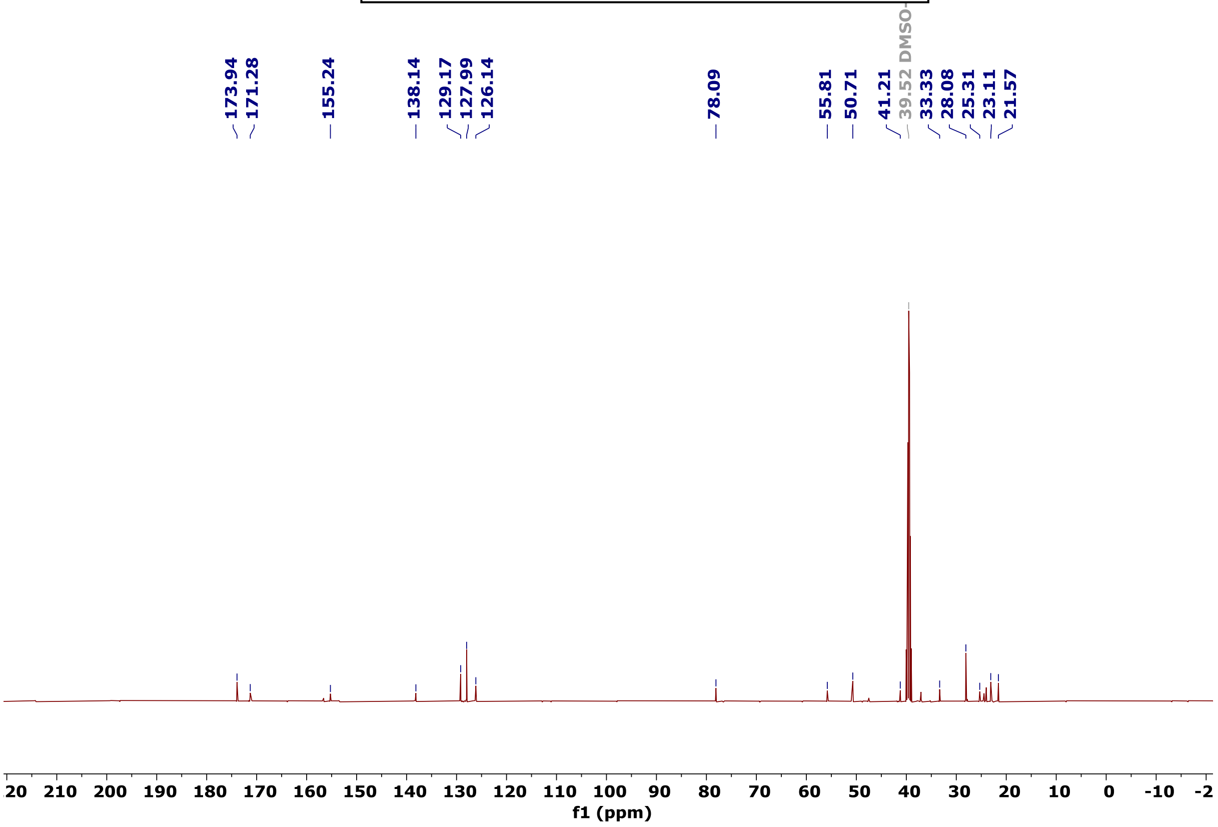

4.2 Characterization of Boc/Cbz/Fmoc-aminoacyl phosphate esters by mass

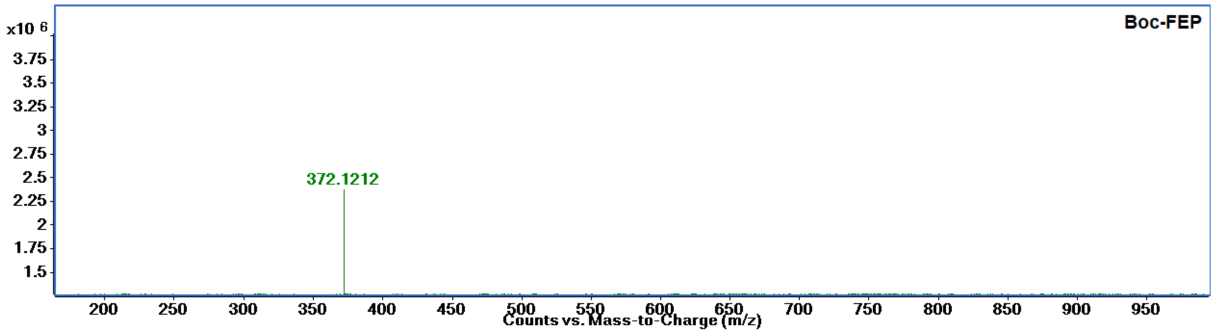

Mass spectra of **Boc-FEP**: Calculated m/z $[M-H]^-$: 372.1218, Observed m/z $[M-H]^-$: 372.1212.

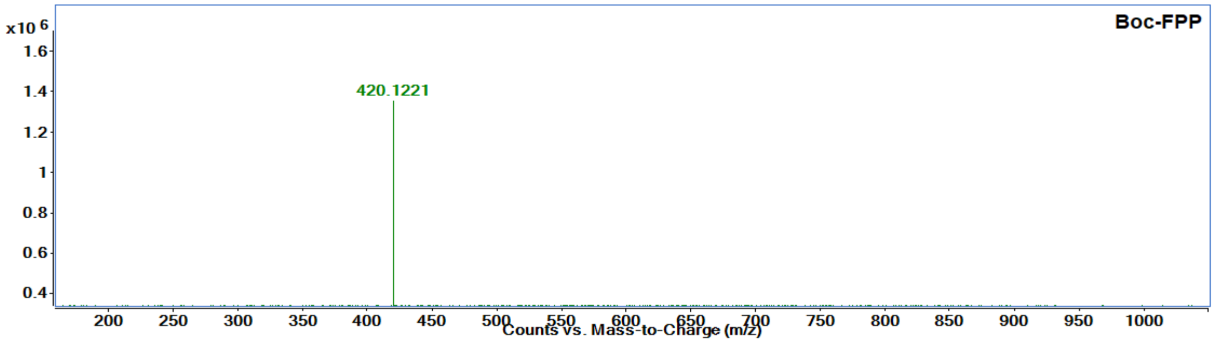

Mass spectra of **Boc-FPP**: Calculated m/z $[M-H]^-$: 420.1218, Observed m/z $[M-H]^-$: 420.1221.

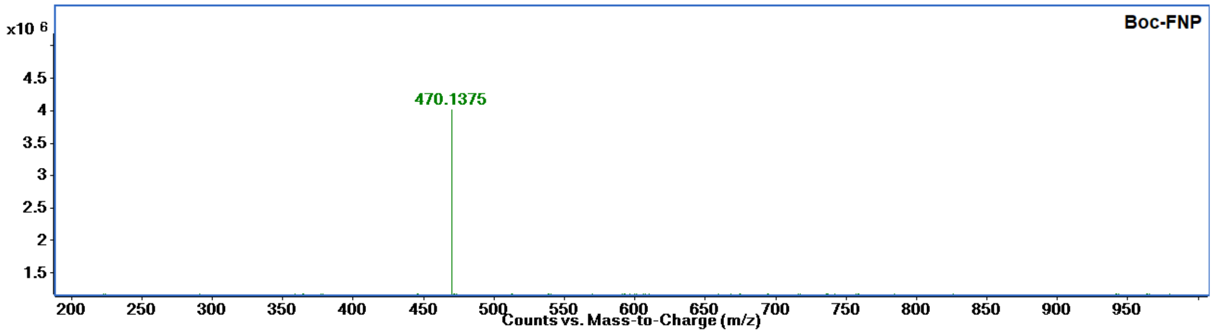

Mass spectra of **Boc-FNP**: Calculated m/z $[M-H]^-$: 470.1374, Observed m/z $[M-H]^-$: 470.1375.

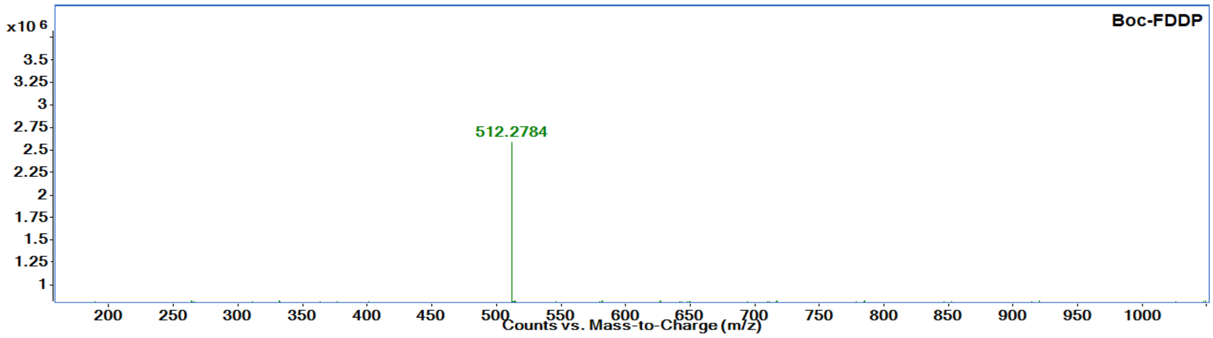

Mass spectra of **Boc-FDDP**: Calculated m/z $[M-H]^-$: 512.2783, Observed m/z $[M-H]^-$: 512.2784.

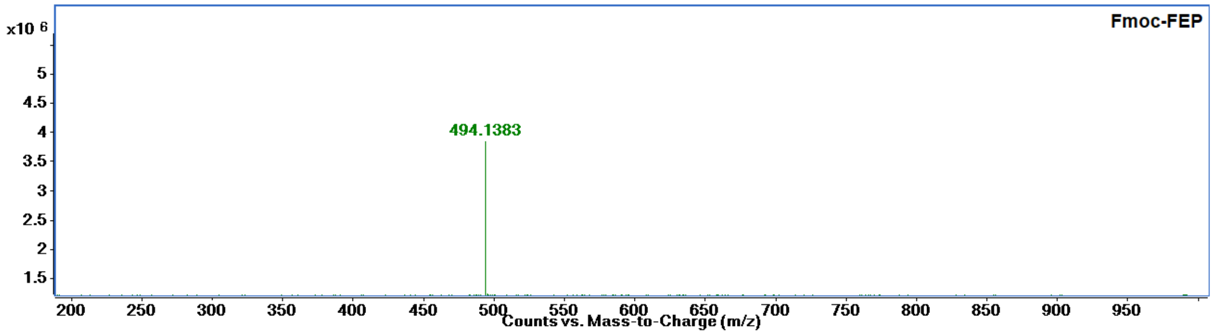

Mass spectra of **Fmoc-FEP**: Calculated m/z [M-H]$^-$: 494.1374, Observed m/z [M-H]$^-$: 494.1383.

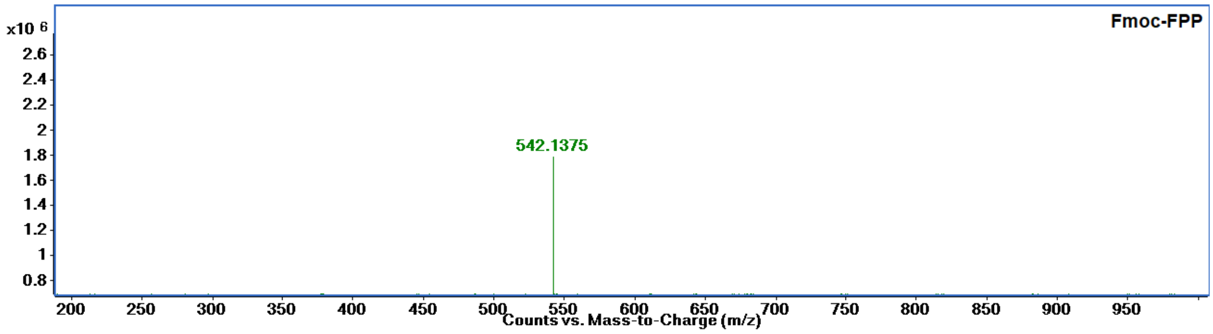

Mass spectra of **Fmoc-FPP**: Calculated m/z [M-H]$^-$: 542.1374, Observed m/z [M-H]$^-$: 542.1375.

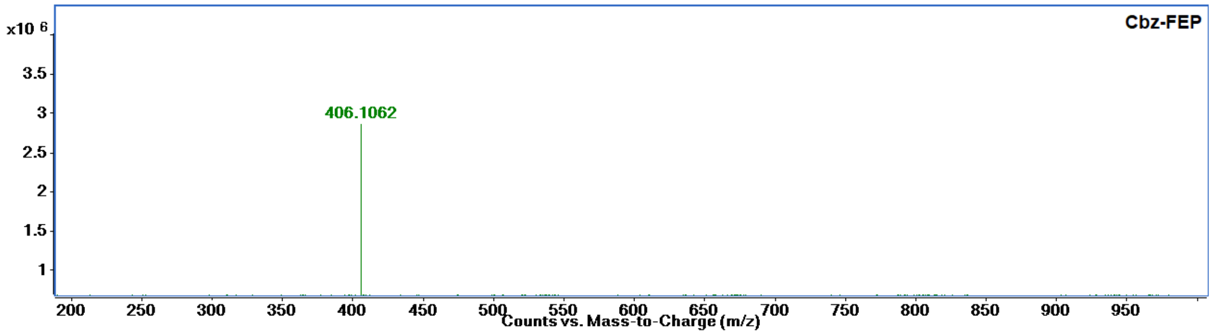

Mass spectra of **Z-FEP**: Calculated m/z [M-H]$^-$: 406.1061, Observed m/z [M-H]$^-$: 406.1062.

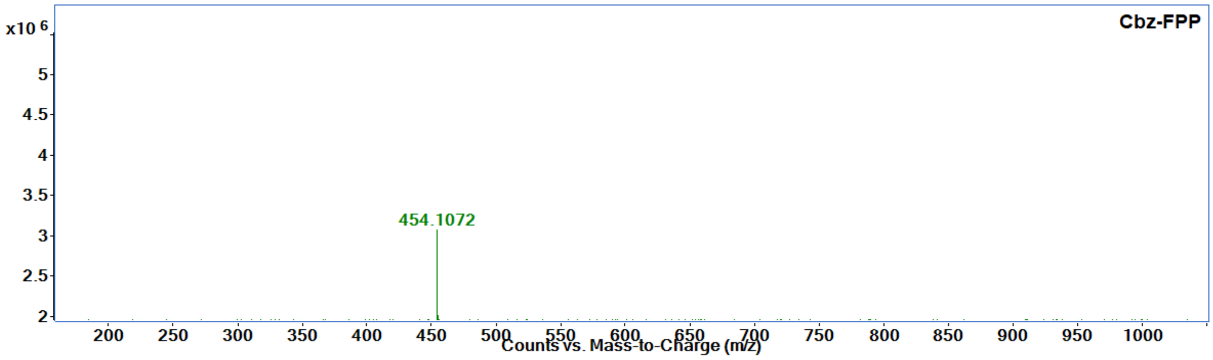

Mass spectra of **Z-FPP**: Calculated m/z [M-H]$^-$: 454.1061, Observed m/z [M-H]$^-$: 454.1072.

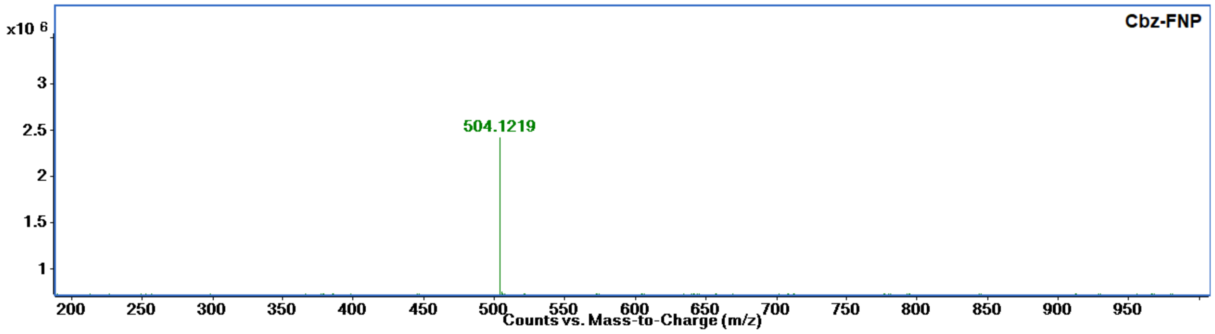

Mass spectra of **Z-FNP**: Calculated m/z [M-H]⁻: 504.1218, Observed m/z [M-H]⁻: 504.1219.

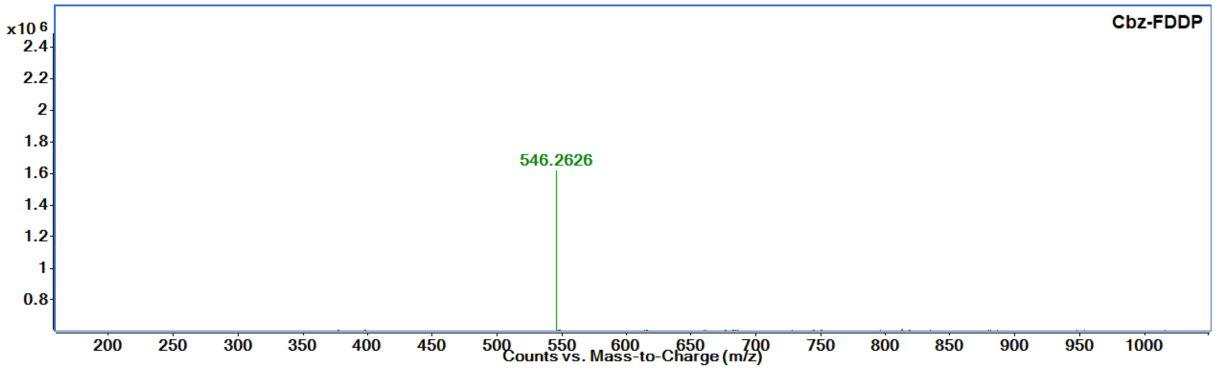

Mass spectra of **Z-FDDP**: Calculated m/z [M-H]⁻: 546.2626, Observed m/z [M-H]⁻: 546.2626.

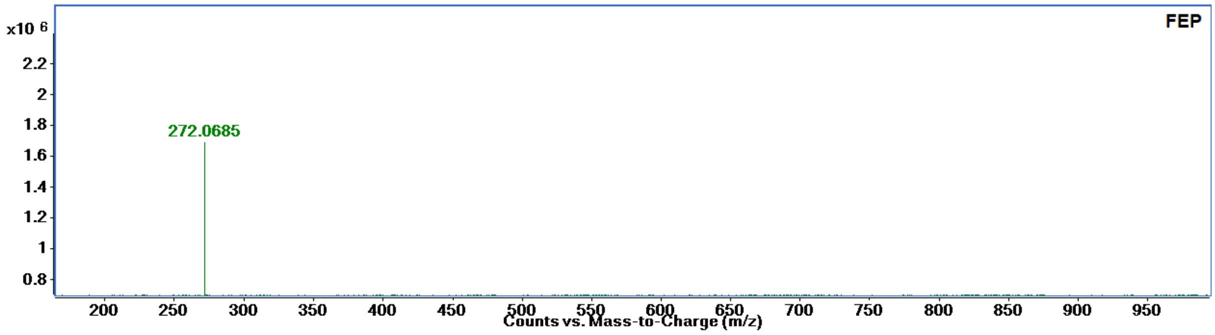

Mass spectra of **FEP**: Calculated m/z [M-H]⁻: 272.0693, Observed m/z [M-H]⁻: 272.0685.

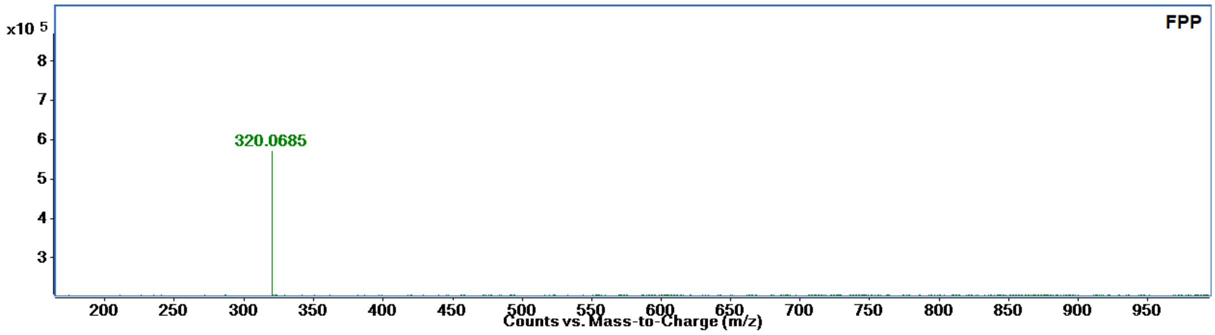

Mass spectra of **FPP**: Calculated m/z [M-H]⁻: 320.0693, Observed m/z [M-H]⁻: 320.0685.

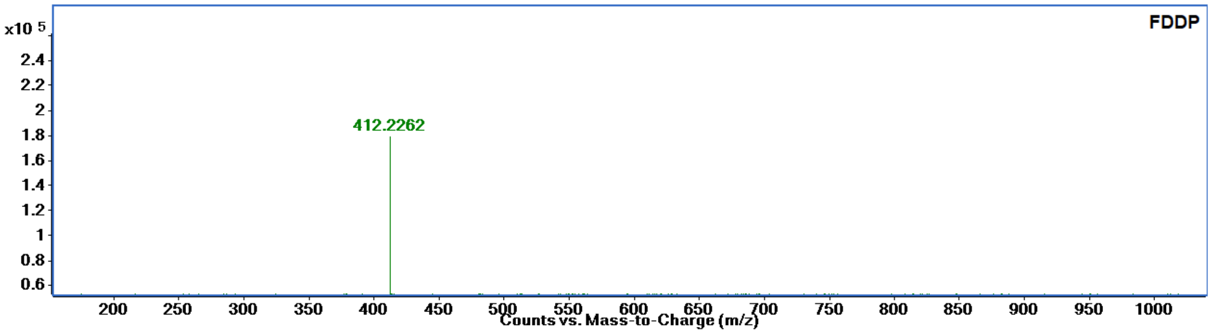

Mass spectra of **FDDP**: Calculated m/z [M-H]$^-$: 412.2258, Observed m/z [M-H]$^-$: 412.2262.

5 References

1. Dai, K.; Pol, M. D.; Saile, L.; Sharma, A.; Liu, B.; Thomann, R.; Trefs, J. L.; Qiu, D.; Moser, S.; Wiesler, S.; Balzer, B. N.; Hugel, T.; Jessen, H. J.; Pappas, C. G., Spontaneous and Selective Peptide Elongation in Water Driven by Aminoacyl Phosphate Esters and Phase Changes. *J. Am. Chem. Soc.* **2023**, *145* (48), 26086-26094

2. Sharma, A.; Dai, K.; Pol, M. D.; Thomann, R.; Thomann, Y.; Roy, S. K.; Pappas, C. G. Selective Peptide Bond Formation via Side Chain Reactivity and Self-Assembly of Abiotic Phosphates. *Nat. Commun.* **2025**, *16* (1), 1306

3. Pol, M. D.; Dai, K.; Thomann, R.; Moser, S.; Kanti Roy, S.; Pappas, C. G. Guiding Transient Peptide Assemblies with Structural Elements Embedded in Abiotic Phosphate Fuels. *Angew. Chem.* **2024**, *136* (28), e202404360

4. Leman, L. J.; Orgel, L. E.; Ghadiri, M. R. Amino acid dependent formation of phosphate anhydrides in water mediated by carbonyl sulfide. *J. Am. Chem. Soc.* **2006**, *128* (1), 20-21Sheehan,

5. J. C.; Hess, G. P. A New Method of Forming Peptide Bonds. *J. Am. Chem. Soc.* **1955**, *77*, 1067-1068
